# Binary and Halide-free Catalyst Systems Based on Al/Ga/In Aminopyridylbisphenolate Complexes for the Cycloaddition of Epoxides and CO_2_

**DOI:** 10.1021/acs.inorgchem.4c02352

**Published:** 2024-08-02

**Authors:** Jesús Damián Burgoa, Lucía Álvarez-Miguel, Marta E. G. Mosquera, Alex Hamilton, Christopher J. Whiteoak

**Affiliations:** † Departamento de Química Orgánica y Química Inorgánica, Facultad de Farmacia and Instituto de Investigación Química Andrés M. del Río (IQAR), Universidad de Alcalá, Grupo SOSCATCOM, Campus Universitario, Ctra. Madrid-Barcelona Km. 33,600, Alcalá de Henares 28871, Madrid, Spain; ‡ Biomolecular Sciences Research Centre (BMRC) and Department of Biosciences and Chemistry, College of Health, Wellbeing and Life Sciences Howard Street, Sheffield Hallam University, Sheffield S1 1WB, U.K.

## Abstract

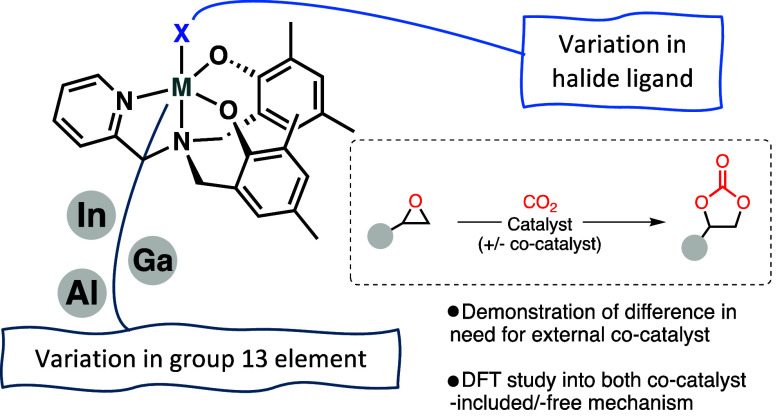

Group 13 complexes bearing an aminopyridylbisphenol ligand have been prepared [**ML-X**; L = ligand, M = Al (X = Cl and Br), Ga (X = Cl, Br, and I), or In (X = Cl)]. The structures of the complexes containing the chloride ligand (**ML-Cl**; M = Al, Ga, and In) have been directly compared through an X-ray crystallography study, with differences in the monomeric or dimeric nature of their structures observed. All of the complexes obtained have been studied as potential catalysts for the synthesis of cyclic carbonates from epoxides and CO_2_. It has been found that the indium complex, as part of a traditional binary catalyst system (catalyst + *tetra*-butylammonium halide cocatalyst), displays the highest catalytic activity and is active under rather mild reaction conditions (balloon pressure of CO_2_). Meanwhile, it has been found that the **GaL-I** complex is a competent single-component catalyst (no need for addition of a cocatalyst) at more elevated reaction temperatures and pressures. A full substrate scope has been performed with both developed catalyst systems to demonstrate their applicability. In addition to the experimental results, a density functional theory study was performed on both catalyst systems. These results explain both why the indium catalyst is the most active under binary catalyst system conditions and how the gallium catalyst with an iodide (**GaL-I**) is able to act as a single-component catalyst in contrast to the indium-based complex.

## Abstract 2

Aluminum, gallium, and indium complexes bearing an aminopyridylbisphenolate ligand have been prepared. These complexes have been applied as catalysts for the synthesis of cyclic carbonates from epoxides and CO_2_. It has been found that the most efficient catalyst is based on indium, as part of a binary catalyst system. Meanwhile, it has been possible to apply a gallium complex as part of a single-component catalyst system (no requirement for addition of an external cocatalyst). A DFT study compliments the obtained experimental results and explains the observed reactivities.

## Introduction

**Figure 1:**
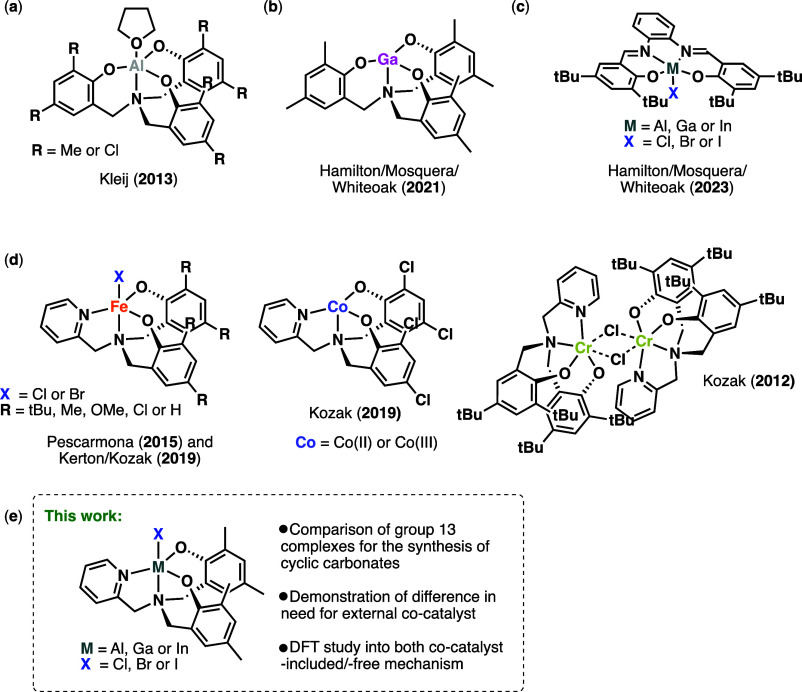
(a,b) Examples of group 13 aminotrisphenolate complexes which have been shown to be active for the cycloaddition reaction.^4a,5^ (c) A recent report comparing the activities of the catalysts based on the heavier group 13 elements against those of aluminum.^7^ (d) Examples of aminopyridylbisphenolate complexes which have been successfully applied for cycloaddition/copolymerization of CO_2_ and epoxides.^11−14^ (e) An overview of the work described in this study.

The synthesis of cyclic carbonates through the atom-efficient cycloaddition of carbon dioxide (CO_2_) and epoxides is attracting a lot of attention. The likely main reasons for this are that it provides a nonreductive sustainable approach to the utilization of CO_2_ and the cyclic carbonate products have found wide utility (e.g., electrolytes in Li-ion batteries, polar aprotic solvents, and reactive intermediates en route to more valuable compounds).^1^ While this reaction can be carried out with only the addition of a strong nucleophile, in this case, it requires unfavorably high temperatures. In response to this, many research groups have developed catalyst systems capable of using milder reaction conditions and/or lower nucleophile loadings, thus further enhancing the overall sustainability of a highly attractive atom-efficient CO_2_ utilizing reaction. Two main catalytic approaches can be identified; organo^2^ and Lewis acid catalysis.^3^ Through both of these approaches, an ever increasing number of catalyst systems has been reported. Of particular relevance to this work are the reports related to group 13-based catalyst systems. Indeed, some of the most active catalysts have been based on aluminum.^4^ Within these examples, in 2013, Kleij and co-workers reported on a highly active aluminum aminotrisphenolate binary catalysts system (catalyst/*tetra*-butylammonium halide) (Figure 1a).^4a^ More recently, in 2021, Whiteoak and co-workers further advanced the field reporting on the first highly active gallium-based binary catalyst system for the synthesis of cyclic carbonates from CO_2_ and epoxides (Figure 1b).^5,6^ Notably, this catalyst was found in general to be more active than its aluminum congener. With an interest in exploiting the potential catalytic properties of the heavier group 13 elements for the cycloaddition reaction, the same authors explored a range of group 13 complexes bearing a salphen ligand (Figure 1c).^7^ This work demonstrates that the indium complexes were more active than those based on gallium/aluminum.

Although the corresponding indium aminotrisphenolate complex has been prepared,^8^ it is highly unstable and requires strong donor ligands to stabilize it (e.g., DMAP or pyridine). As these ancillary ligands are strongly bound, they inhibit catalytic potential.^9^ With this issue identified and with a further recent report on the interesting chemistry of the heavier group 13 elements in the context of Lewis acid catalysis,^10^ we proposed the use of a related aminopyridylbisphenol ligand. In the case of metal(III) complexes of this aminopyridylbisphenol ligand, in contrast to the complexes of aminotrisphenolate, there will be a negatively charged ligand present (e.g., a halide) which helps to stabilize the complex in the same manner as the strongly coordinating ligands of the aforementioned aminotrisphenolate complex. Importantly, complexes with this aminopyridylbisphenol ligand and the halide will still have a vacant coordination site, where catalysis can occur.

In the context of cyclic and polycarbonate synthesis from the cycloaddition/copolymerization of CO_2_ and epoxides, several examples of catalysts based on the aminopyridylbisphenol ligand have been reported (Figure 1d), particularly from the groups of Kozak, Kerton, and Pescarmona.^11−15^ With these precedents, we decided to explore the synthesis and comparative catalytic activities of group 13 aminopyridylbisphenolate complexes. It should be noted that Mountford and co-workers have previously described the use of indium aminopyridylbisphenolate complexes for the ring-opening polymerization of *rac*-Lactide^16^ and that Briand and co-workers have prepared the methylindium aminopyridylbisphenolate complex, although no catalytic results were reported.^17^ Herein, we describe the results obtained from this study and demonstrate that through the variation of the metal and halide, a single-component catalyst system (no need for the addition of the external nucleophile) could also be unexpectedly developed. Both the binary and single-component mechanisms have also been fully elucidated using a density functional theory (DFT) study in order to fully understand the critical differences in the catalytic systems.

## Results and Discussion

### Synthesis and Characterization of the Complexes

**Scheme 1:**
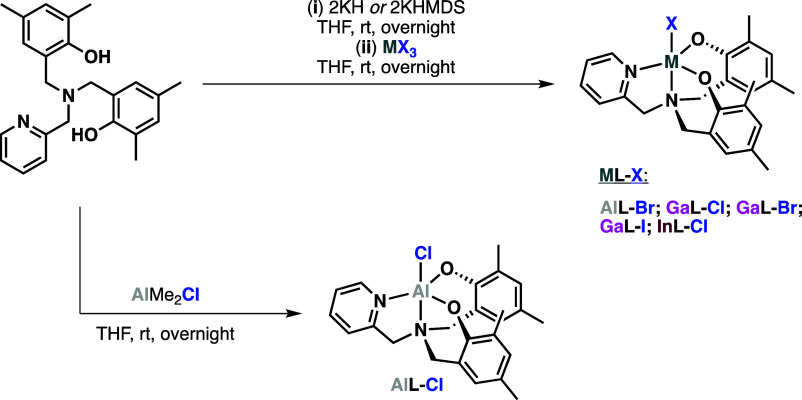
Synthetic Approaches for the Preparation of the Complexes (**ML-X**; **M** = Metal, **L** = Ligand, and **X** = Halide) Used in This Work The structures presented are simplified and do not consider the possibility of dimerization or solvent coordination (e.g. THF).

The approach to the synthesis of the group 13 complexes (aluminum, gallium, and indium) was similar to that of our previous report on group 13 salphen complexes.^7^ Two approaches were used (Scheme 1); to obtain the aluminum compound with a chloride ligand, dimethyl aluminum chloride (AlMe_2_Cl) was directly reacted with the ligand (H_2_L) in an equimolar ratio, furnishing **AlL-Cl**. Meanwhile, all other complexes, **ML-X** (**M** = Al; **X** = Br. **M** = Ga; **X** = Cl, Br, or I. **M** = In; and **X** = Cl), were obtained through salt metathesis of the potassium salt of the ligand (K_2_L) and the metal trihalide (MX_3_). The potassium salt of the ligand was formed by reaction of the ligand with either potassium hydride (KH) or potassium *bis*(trimethylsilyl)amide (KHMDS). In some cases, the KH approach provided better yields, while in others improved yields were obtained using KHMDS (see the Experimental Section for the base used in each case). While most of the group 13 complexes could be prepared using one or other of the synthetic approaches, despite vigorous attempts and alternative approaches, neither **AlL-I**, **InL-Br**, nor **InL-I** could be obtained.

### Solid State Structures

**Figure 2:**
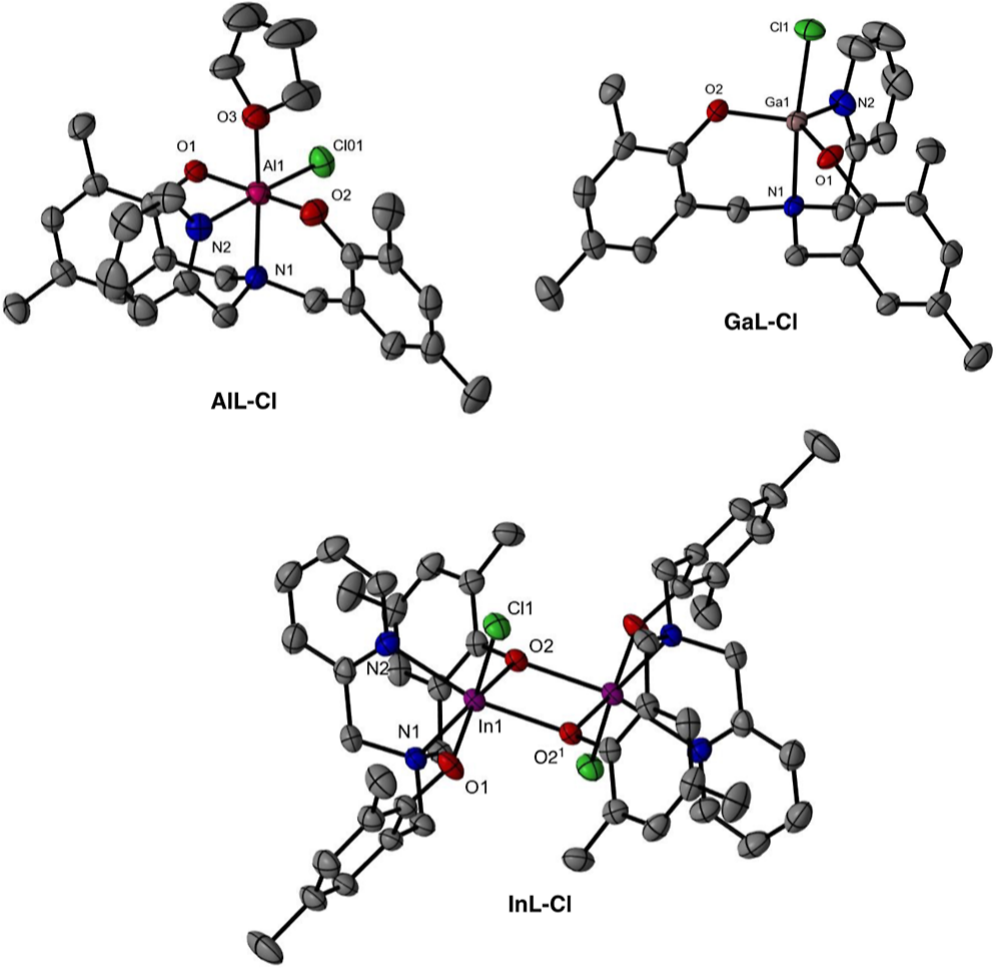
X-ray crystal structures obtained for the **ML-Cl** complexes (M = Al, Ga, and In). Note: after refinement of the **Al–Cl** complex, some alerts remain in the checkCIF file and are attributed to disorder in the free-THF molecule.

Single crystals of **ML-Cl** (M = Al, Ga, or In) suitable for X-ray diffraction studies were isolated and the X-ray crystal structures of the three complexes were obtained (see Figure 2 and selected bond distances and angles, Table 1). The data for the aluminum complex, **AlL-Cl**, were sufficient to obtain the structure and connectivity. The molecule presents the aminopyridylbisphenolate ligand in a *tetra*-coordinated environment, with both a coordinated THF molecule and a chloride ligand providing the octahedral geometry. The coordinated THF molecule is included during the crystal growth. It should be noted that the ^1^H NMR for characterization purposes had to be obtained in deuterated DMSO as the NMR was interpretable. Indeed, the compound does dissolve in deuterated chloroform, but NMR is rather complicated and cannot be suitably interpreted. This suggests that in the bulk solution, the DMSO is acting in the same way as the THF molecule in the solid state, allowing for a discrete monomeric species. Meanwhile, in the related gallium complex, **GaL-Cl**, the gallium atom exhibits a pentacoordinate environment. The τ^5^ value^18^ of this complex, 0.84, demonstrates that the complex has a distorted trigonal bipyramidal geometry which also shows a hydrogen bonding interaction between the chloride ligand and the aromatic proton of the pyridyl moiety [Cl(1)–H(24) bond distance 2.536 Å]. In stark contrast, the indium complex, **InL-Cl**, reveals a dinuclear structure, which is formed by bridging phenoxides of the ligand between two indium atoms. Each indium atom is coordinated both to the aminopyridylbisphenolate ligand and a chloride ligand, with two unsymmetrical phenoxy-bridges [In(1)–O(2) of 2.229(2) and 2.178(2) Å]. Indium complexes containing phenoxy-bridges are scarce and not many structures have been described.^19^ Additionally, this structure also displays a hydrogen bond interaction between the chloride ligand and one proton of the methylene connecting the amine and phenolate moiety [Cl(1)–H(10B) = 2.476 Å]. As expected, as the group 13 element increases in size, the Cl, N, and O bond distances also increase. However, in the case of the Ga(1)–N(2) bond distance, the observed value is shorter than the expected one when considering the expected trend of increasing elongation when going down in group 13 [Al/Ga/In, 2.079(3)/2.028(3)/2.311(3) Å]. Indeed, this shortening of the Ga–N bond may be a result of the five-coordinate gallium center versus the six-coordinate aluminum and indium centers.

**Table 1:** Selected Bond Distances (Å) and Bond Angles (deg) for the **ML-Cl** Complexes (M = Al, Ga, and In)^a^

**AlL-Cl**		**GaL-Cl**		**InL-Cl**	
Selected Bond Distances (Å)					
Al(1)–Cl(1)	2.282(1)	Ga(1)–Cl(1)	2.2829(9)	In(1)–Cl(1)	2.429(1)
Al(1)–N(1)	2.084(3)	Ga(1)–N(1)	2.196(3)	In(1)–N(1)	2.352(2)
Al(1)–N(2)	2.079(3)	Ga(1)–N(2)	2.028(3)	In(1)–N(2)	2.311(3)
Al(1)–O(1)	1.809(2)	Ga(1)–O(1)	1.836(2)	In(1)–O(1)	2.071(2)
Al(1)–O(2)	1.791(2)	Ga(1)–O(2)	1.836(2)	In(1)–O(2)	2.178(2)
Al(1)–O(3)	1.973(2)			In(1)–O(2)^1^	2.229(2)
Selected Bond Angles (deg)					
N(1)–Al(1)–N(2)	81.60(11)	N(1)–Ga(1)–N(2)	78.95(11)	N(1)–In(1)–N(2)	73.45(9)
O(1)–Al(1)–Cl(01)	93.10(8)	O(1)–Ga(1)–Cl(01)	92.71(8)	O(1)–In(1)–Cl(1)	91.05(7)
O(2)–Al(1)–Cl(01)	94.29(9)	O(2)–Ga(1)–Cl(01)	92.54(8)	O(2)–In(1)–Cl(1)	104.22(5)
N(1)–Al(1)–Cl(01)	94.02(8)	N(1)–Ga(1)–Cl(01)	172.72(7)	N(1)–In(1)–Cl(1)	158.48(6)
O(1)–Al(1)–O(2)	171.33(12)	O(1)–Ga(1)–O(2)	122.35(12)	O(1)–In(1)–O(2)	158.87(9)
				O(2)–In(1)–O(2)	70.15(8)

a After refinement of the **Al–Cl** complex, some alerts remain in the checkCIF file and are attributed to the disorder in the free-THF molecule.

### Catalytic Studies for the Cycloaddition of Epoxides and Carbon Dioxide

With a selection of complexes prepared, we sought to compare their efficiency as catalysts for the cycloaddition of epoxides and CO_2_. Styrene oxide was selected as substrate and *tetra*-butylammonium iodide (TBAI) as the cocatalyst (Table 2). Initially, all six of the available compounds were studied at 8.0 bar of CO_2_ pressure, 0.25 mol % of catalyst, and 1.25 mol % of TBAI, at room temperature. We chose a molar ratio of 1:5 (catalyst/cocatalyst) as this has previously been found to be optimal with the gallium aminotrisphenolate catalysts.^5^ It was found that both aluminum complexes (Cl and Br) provided very similar results, 13 and 14%, respectively (Table 2, entries 1 and 2). There was an increase in activity when moving from aluminum to gallium, in the order of a doubling of the activity compared to aluminum (Table 2, entries 3, 4, and 5). Again, there was little difference between the three distinct halides. Finally, moving to indium provided a catalyst which was more active than the aluminum and gallium counterparts (Table 2, entries 1, 3, and 5). This increase in activity going down the group is similar to what was previously observed in our previous study with group 13 salphen catalysts.^7^

**Table 2:**
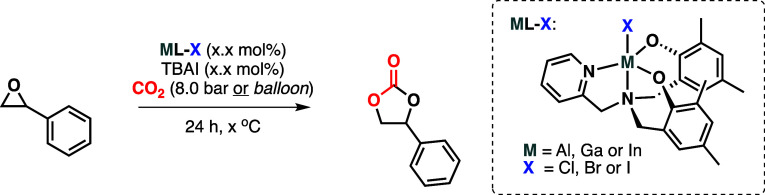
Initial Comparative Screening of Group 13 Aminopyridylbisphenolate Complexes as Catalysts for the Conversion of Styrene Oxide and CO_2_ into Styrene Carbonate and Subsequent Reaction Condition Optimization of the **InL-Cl**/TBAI Binary Catalyst System^a^,^e^

entry	catalyst	catalyst [mol %]	TBAI [mol %]	temperature [°C]	yield [%]^b^
8.0 bar CO_2_ (Time 18 h)					
1	**AlL-Cl**	0.25	1.25	25	13
2	**AlL-Br**				14
3	**GaL-Cl**				26
4	**GaL-Br**				30
5	**GaL-I**				26
6	**InL-Cl**				49
Balloon of CO_2_ (Time 24 h)					
7	**InL-Cl**	0.4	1.0	25	34
8		0.4	1.0	40	74
9^c^		0.4	1.0	40	74
10^d^		0.4	1.0	40	23
11		0.8	2.0	40	90
12		1.0	2.5	40	97
**13**		**0.8**	**2.0**	**60**	**>99**
14		0.6	1.5	60	97
15			2.0	60	39
16		0.8		60	trace

a General reaction conditions: 10.0 mmol styrene oxide, **ML-X** (*x*.x mol %), TBAI (*x*.x mol %), 8.0 bar or balloon pressure of CO_2_, 18 or 24 h.

b Calculated from analysis of the ^1^H NMR spectra of the crude reaction mixture; in all cases, the selectivity to the styrene carbonate product was >99%.

c *Tetra*-butylammonium bromide (TBAB) was used as a cocatalyst.

d *Tetra*-butylammonium chloride (TBACl) was used as a cocatalyst.

e mol % of the indium catalyst is based on the mol % of indium (the monomeric unit).

With the knowledge that the indium compound, **InL-Cl**, was the most active catalyst under the initial screening conditions, a further and more in-depth optimization was embarked upon. At present, the state-of-the-art is moving toward the use of low-pressure sources of CO_2_ and in this context, the next steps in the optimization were carried out using a balloon pressure of CO_2_ (1.0 atm) and attempting to reduce the catalyst/cocatalyst (down to 1:2.5). Initially, with the knowledge that a reduction in both CO_2_ pressure and catalyst/cocatalyst ratio would likely lead to a reduction in activity, the catalyst loading was increased to 0.4 mol % and the reaction time to 24 h while maintaining the reaction temperature at 25 °C (Table 2, entry 7). This indeed provided a lower yield of 34%, but gave evidence that the binary catalyst system based on indium was able to catalyze the reaction under these remarkably mild reaction conditions. Upon increasing the reaction temperature to 40 °C, using the same binary catalyst system loadings, a yield of 74% was obtained (Table 2, entry 8). The use of *tetra*-butylammonium bromide as cocatalyst provided the same yield as the TBAI; meanwhile, the change to *tetra*-butylammonium chloride (TBACl) gave a significantly reduced yield of 23% (Table 2, entries 9 and 10). As such, all further optimization was performed using TBAI. Increasing the catalyst loading to 0.8 and then 1.0 mol % resulted in an increase in the yield to 90 and then 97% (Table 2, entries 11 and 12). With almost quantitative yield obtained, a rise in the temperature using 0.8 mol % catalyst with 2.0 mol % TBAI was trialed (Table 2, entry 13). This experiment provided a quantitative yield and was thereafter considered to be the optimal reaction conditions for this indium-based binary catalyst system. A reduction in the catalyst loading to 0.6 mol % (and corresponding reduction of cocatalyst to 1.5 mol %) at 60 °C resulted in a slight decrease in yield to 97% (Table 2, entry 14). The cocatalyst alone was also studied and provided a yield of 39% under these reaction conditions; meanwhile, the presence of only the group 13 catalyst led to trace amounts of styrene carbonate (Table 2, entries 15 and 16, respectively).

**Scheme 2:**
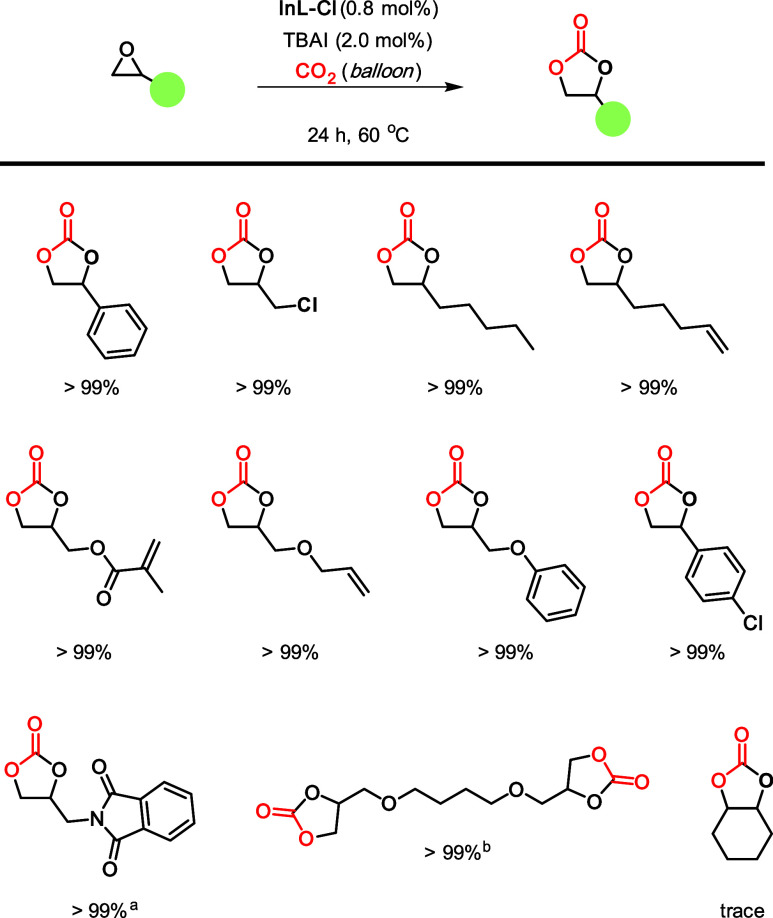
Substrate Scope Using the **InL-Cl**/TBAI Binary Catalyst System General conditions: 10.0 mmol substrate, 0.8 mol % **InL-Cl**, 2.0 mol % TBAI, 60 °C, 24 h, balloon pressure of CO_2_. 1 mL of methylethylketone (MEK) was used as the solvent. 5.0 mmol of substrate used. Note: mol % of the indium catalyst is based on the mol % of indium (the monomeric unit).

With the catalyst system based on **InL-Cl** optimized, a substrate scope was studied (Scheme 2). It was observed that a range of terminal epoxides could be successfully converted under these relatively mild reaction conditions. This also included a *bis*-epoxide which could be readily and quantitatively converted into the *bis*-cyclic carbonate product. However, the conversion of internal epoxides was found to be rather challenging, and it was not possible to convert cyclohexene oxide. This result demonstrates that although active, this catalyst system still presents some limitations. Indeed, there are some indium-based catalysts reported in the literature which can form either cyclic- or polycarbonates,^7,20^ while others produce low yields, or this challenging substrate has not been studied.^21^

The observation of traces of cyclic carbonate product in the absence of cocatalyst (Table 2, entry 16) aroused our interest. It was hypothesized that the halide ligand could possibly participate as the nucleophile cocatalyst. In order to study this proposal, a cocatalyst-free screening of all the catalysts was attempted (Table 3). Initially, all the catalysts were studied at 80 °C and 0.25 mol %, again using styrene oxide as substrate (Table 3, entries 1–6). Most of the complexes gave trace or very low yields of the styrene carbonate product, except for the aluminum complex with a bromide ligand, **AlL-Br**, and gallium complex which has an iodide ligand, **GaL-I**. In the former case, in the reaction crude, it was possible to see traces of the ligand and not the starting aluminum complex. This suggests that this compound is not stable under the reaction conditions. This instability has also been observed during characterization of this complex; when the ^1^H NMR spectra were recorded in wet DMSO-*d*_6_, a significant proportion of ligand was observed (see the Supporting Information for spectra), indicating that it is easily hydrolyzed. Thus, in this case, it is proposed that the activity arises from the traces of ligand, which is able to catalyze the reaction through a hydrogen-bonding mechanism, similar to that reported by North and co-workers using a metal-free salphen catalyst.^22^ In this context, we have also performed the experiment with our ligand alone and found a 2% conversion. This indicates some activity but less than that reported by North. It should be noted that the hydrolysis of **AlL-Br** will also release aluminum and bromide, which may also combine to enhance this reaction, although we are unable to propose the structure of this compound at this time.

**Table 3:**
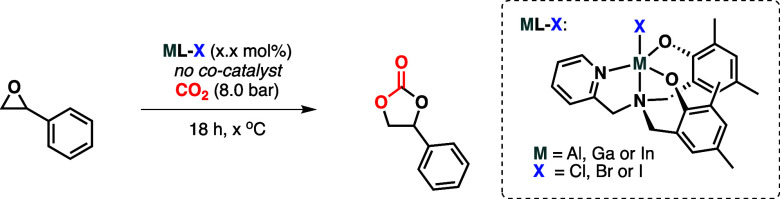
Comparative Screening of Group 13 Aminopyridylbisphenolate Complexes as Single-Component Catalysts (without Additional Cocatalysts) for the Conversion of Styrene Oxide and CO_2_ to Styrene Carbonate and Subsequent Reaction Condition Optimization with the **GaL-I** Catalyst^a^

entry	catalyst	catalyst [mol %]	temperature [°C]	yield [%]^b^
1	**AlL-Cl**	0.25	80	1
2	**AlL-Br**			12
3	**GaL-Cl**			2
4	**GaL-Br**			3
5	**GaL-I**			32
6	**InL-Cl**			traces
7	**GaL-I**	0.25	90	67
8		0.25	110	91
9		0.4	110	95
**10**		**0.6**	**110**	**>99**

a Ge0 mmol styrene oxide, **ML-X** (*x*.x mol %), 8.0 bar of CO_2_, 18 h.

b Calculated from analysis of the ^1^H NMR spectra of the crude reaction mixture; in all cases, the selectivity to the styrene carbonate product was >99%. Note: mol % of the indium catalyst is based on the mol % of indium (the monomeric unit).

In the case of **GaL-I**, a 32% yield was obtained. This result was repeated several times to confirm that this result did not arise from either adventitious impurities or traces of ligand (the starting complex could be observed in the ^1^H NMR of the crude reaction mixture) in the reactor and was found to be very reproducible. With this interesting result in hand, further optimization of the reaction conditions was carried out. First, the reaction temperature was raised from 80 to 90 °C, which resulted in an increase of the yield from 32 to 67% (Table 3, entries 5 and 7). With the observation of this increase in yield, a further increase to 110 °C was studied, and this indeed furnished an increase in styrene carbonate product, resulting in a 91% yield (Table 3, entry 8). Thereafter, raising the catalyst loading to 0.4 and then 0.8 mol % provided an increase in the yield, with the latter producing a quantitative conversion of the substrate in a >99% selectivity (Table 3, entries 9 and 10).

**Scheme 3:**
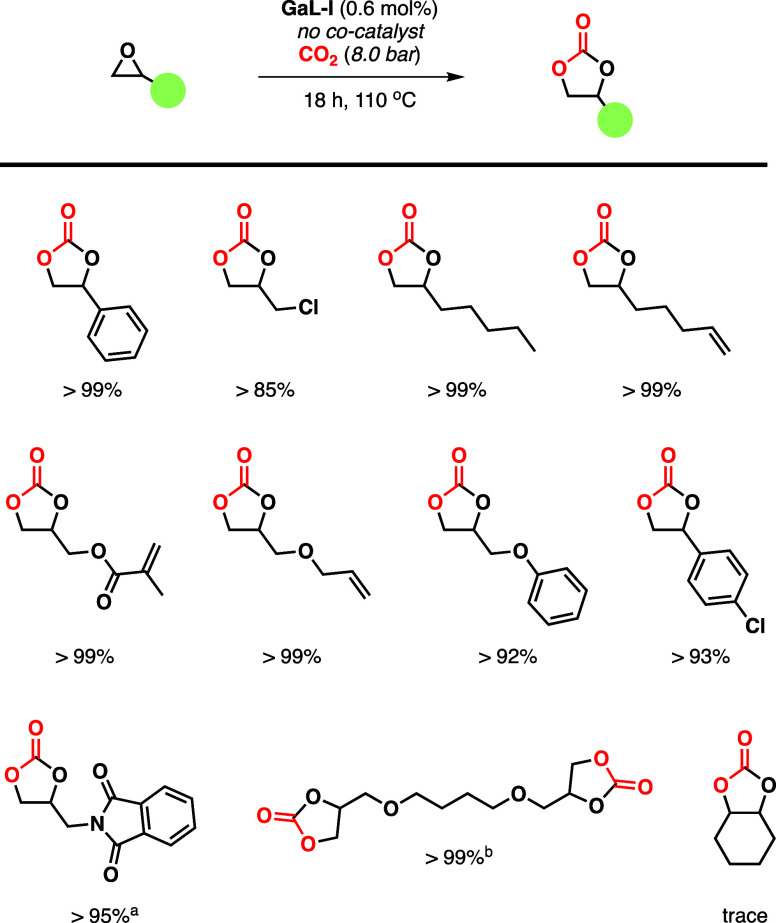
Substrate Scope Using the **GaL-I** Single-Component Catalyst System General conditions: 10.0 mmol substrate, 0.6 mol % **GaL-I**, 110 °C, 18 h, 8.0 bar of CO_2_. 1 mL of methylethylketone (MEK) was used as the solvent. 5.0 mmol of substrate used.

As with the binary catalyst system based on indium, the substrate scope of this single-component gallium-based catalyst system was studied (Scheme 3). Again, it was possible to convert a range of differently functionalized epoxides into excellent to quantitative yields. However, it was not possible to convert the internal epoxide of the cyclohexene oxide.

### Computational Study

With the experimental results in hand, we turned to DFT calculations to elucidate the underlying operative mechanisms in order to understand the distinct activities shown by the gallium and indium catalysts (**GaL-I** vs **InL-Cl**). Given the similarities to the aminotrisphenolate catalysts previously studied,^5^ we expected to be able to directly translate the mechanism to this new binary catalyst system, and in part, this was the case.

**Figure 3:**
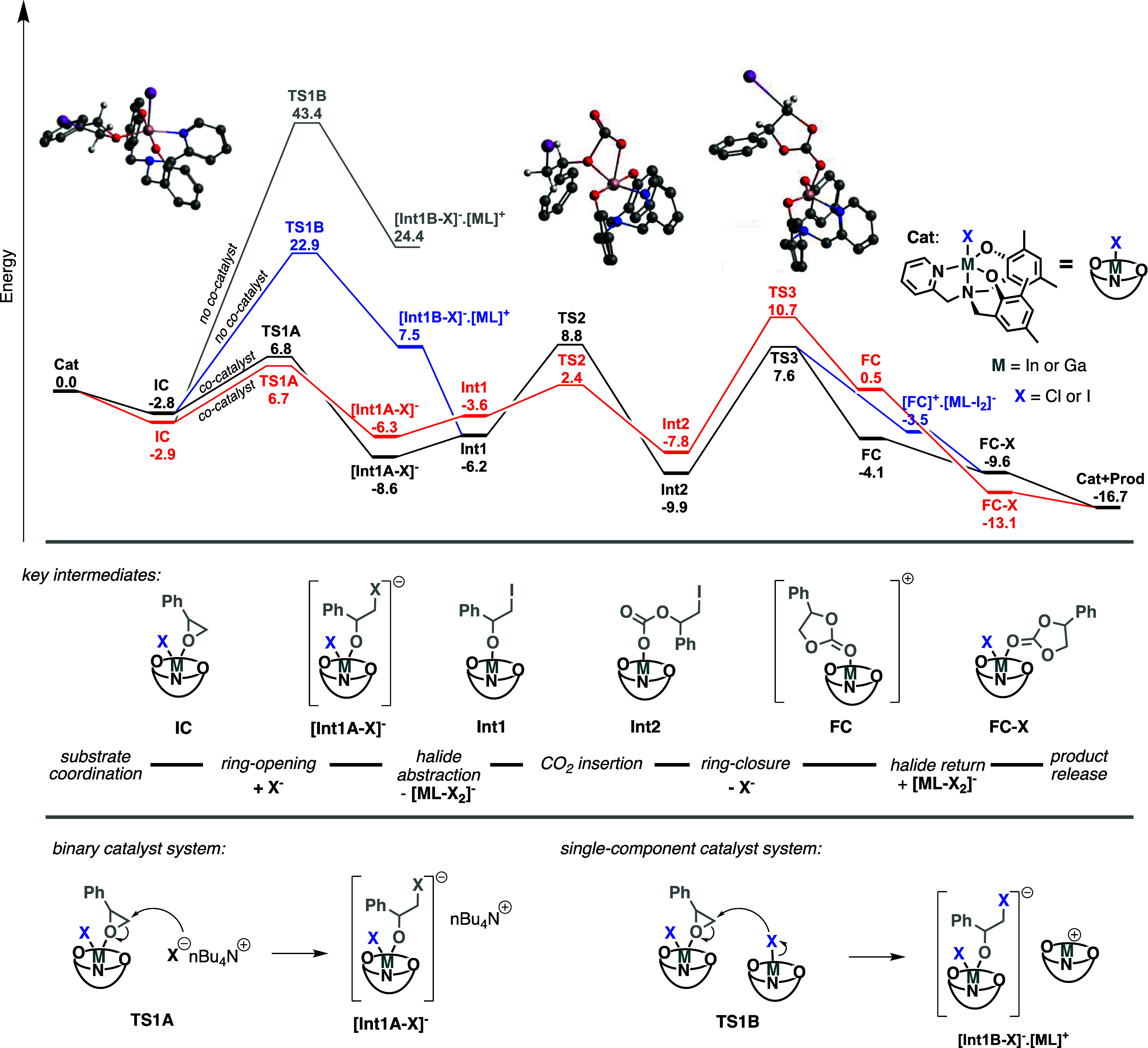
Calculated free energy surface (Δ*G*_298 K_ kcal mol^–1^), ωB97M-V/def2-tzvpp, for cyclic carbonate synthesis using styrene oxide as the substrate, with **GaL-I**/TBAI (black line) and **GaL-I** (cocatalyst free; blue line), **InL-Cl**/TBAI (red line), and **InL-Cl** (cocatalyst free; gray line). Drawn structures relate to the binary catalyst system pathway. Calculated geometries shown relate to **TS1B**, **TS2**, and **TS3**, respectively, along the **GaL-I** cocatalyst free pathway.

Initially focusing on the **GaL-I** catalyst (Figure 3, black line), the addition of the epoxide substrate to the catalyst forms **IC**, which is 2.8 kcal mol^–1^ more stable than the free components. With inclusion of the TBAI cocatalyst in the system, the reaction follows the expected iodide-assisted epoxide ring opening reaction to the coordinated epoxide, though **TS1A** via a barrier of 9.6 kcal mol^–1^ from **IC**. The ring opening step leads to **[Int1A-X]**^**–**^, and this stable intermediate consists of the **GaL-I** catalyst and the newly formed alkoxide ligand which arises from the ring-opening of the epoxide. From **[Int1A-X]**^**–**^, the iodide ligand is abstracted by a second gallium catalyst, resulting in neutral **Int1** and separate anionic **[GaL-I**_**2**_**]**^**–**^ species. From **Int1**, the CO_2_ then inserts into the Ga–O bond, forming linear carbonate intermediate **Int2**. This requirement for halide abstraction prior to CO_2_ insertion is similar to the previously reported reaction with group 13 salphen catalysts.^7^ The overall barrier for CO_2_ insertion (including the iodide abstraction step) is 17.4 kcal mol^–1^. From **Int2**, the cyclic carbonate is then formed with the associated loss of the iodide (regenerating the TABI cocatalyst), forming the cationic **[FC]**^**+**^ complex. Reassociation of the iodide from **[GaL-I**_**2**_**]**^**–**^ forms the thermodynamically more stable **FC-X** complex. Thereafter, the loss of the cyclic carbonate product results in the regeneration of the initial catalyst species. It could be envisioned that the halide abstraction from **[Int1A-X]**^**–**^ could also be facilitated by the **TBA**^**+**^ ion, and likewise the reassociation at **[FC]**^**+**^, by **TBAI**. The relative energy difference for these competing processes (**TBA**^**+**^/**GaL** and **TBAI**/**[GaL-I**_**2**_**]**^**–**^) is only 0.6 kcal mol^–1^ and therefore within the error of the calculations. The important result is the requirement that the halide abstraction/reassociation processes are needed to occur to facilitate the reaction.

As has also been presented in this study, the **GaL-I** complex shows substantial activity without the requirement for the external TBAI cocatalyst, albeit less than the corresponding binary catalyst system (higher catalyst system loading/increased temperature). The mechanism for this cocatalyst-free pathway is also shown in Figure 3 (blue line). From the initially catalyst/epoxide species, **IC**, a second **GaL-I** catalyst provides the iodide for the ring-opening of the epoxide. This process is energetically less favorable compared to the binary catalyst system TBAI route, with a barrier of 22.9 kcal mol^–1^, but is still energetically accessible at moderate temperatures. The equivalent **[Int1B-X]**^**–**^**.[GaL]**^**+**^ ion pair is 13.8 kcal mol^–1^ less stable compared to **[Int1A-X]**^**–**^ on the binary catalyst system approach (Figure 3, black vs blue lines). Thereafter, transfer of the halide from **[Int1B-X]**^**–**^ to **[GaL]**^**+**^ allows for rejoining of the original (binary) catalyst system pathway. The proceeding CO_2_ insertion occurs as previously described, via **TS2**. The formation of the cyclic carbonate product, via **TS3**, is assisted by a second catalyst species, transiently forming the **[FC]**^**+**^**·[GaL-I**_**2**_**]**^**–**^ species, before transferring the halide and forming the **FC-X** intermediate. The loss of the cyclic carbonate product regenerates the active catalyst. The overall energy span for the binary catalyst system pathway is 17.5 kcal mol^–1^ compared to 25.7 kcal mol^–1^ for the cocatalyst-free approach, agreeing with the higher experimentally required temperatures for the latter. A ring-opening mechanism using the halide bound directly to the catalyst was also explored but was found to be energetically inaccessible and this is in line with the experimental results.

Moving on to consider the **InL-Cl** catalyst, the reaction for the binary catalyst system pathway (Figure 3, red line) is very similar to that of the **GaL-I** catalyst. Iodide-assisted ring-opening of the epoxide leads to the alkoxide intermediate requiring abstraction of the metal chloride, forming **[InL-Cl**_**2**_**]**^**–**^. Interestingly and rather notable is that the energy requirement for the CO_2_ insertion is significantly lower for the **InL-Cl** catalyst compared to that of the **GaL-I** catalyst (8.7 kcal mol^–1^ compared to 17.4 kcal mol^–1^, respectively). This can be explained by the weaker In–O interaction compared to that of the corresponding Ga–O. From the linear carbonate intermediate, ring-closure occurs with loss of the iodide via **TS3**, reforming the TBAI cocatalyst. This is the energy span determining step of the mechanism, with a barrier of 18.5 kcal mol^–1^ from **Int2**. Thereafter, transfer of a chloride from **[InL-Cl**_**2**_**]**^**–**^ forms the neutral **FC-X** intermediate, with the loss of the product allowing for the continuation of the catalytic cycle. As presented, the **InL-Cl** catalyst does not show cocatalyst free activity. Shown in gray in Figure 3 is the barrier for chloride-assisted ring opening, utilizing **InL-Cl** as the chloride source, subsequently forming a cationic **[InL]**^**+**^ species. This barrier is 46.3 kcal mol^–1^ and hence inaccessible under the experimental conditions in this study; therefore, these DFT study results are in full agreement with the experimental observations. This increased activation barrier comes from the formation of the unstable **[InL]**^**+**^ species, not the reduced nucleophile strength in moving from iodide to chloride, as the barrier to chloride-assisted epoxide ring-opening with TBACl is energetically equivalent to that of TBAI, 9.8 and 9.6 kcal mol^–1^, respectively.

## Conclusions

In conclusion, a series of complexes based on the heavier group 13 elements (Al, Ga, and In) and an aminopyridylbisphenol ligand have been prepared (**ML-X**; M = Al, Ga, or In; L = ligand; and X = Cl, Br, or I). In the cases of complexes **AlL-Cl**, **GaL-Cl**, and **InL-Cl**, their X-ray crystal structures have been obtained, allowing for direct comparison of the effect of changing the element on their structures. It has been observed that the aluminum and gallium compounds are monomeric; meanwhile, the analogous indium complex has been found to be dinuclear in the solid state. All of the complexes have been studied as catalysts for the conversion of epoxides and CO_2_ to cyclic carbonates under extremely low pressures of CO_2_ (balloon pressure). It has been found that the indium complex is more active than the gallium complexes, and these in turn are more active than the aluminum complexes. This result is similar to our previous study using group 13 salphen complexes^7^ and continues to identify the heavier group 13 elements as an interesting alternative to well-studied aluminum compounds in this field. In addition, we have demonstrated that the **GaL-I** complex is also active in the absence of an external cocatalyst. Both the binary catalyst and single-component catalyst systems have been explored through a DFT study, whereby the experimental results are fully in alignment with these computational results. In the case of the single-component catalyst system, the energy barriers for the indium-based catalysts have been found to be much higher than those for the corresponding gallium catalyst. In summary, this work provides (i) an interesting example of the higher activities of the heavier group 13 elements as the basis for catalysts for the synthesis of cyclic carbonates from epoxides and CO_2_, (ii) an example of a catalyst system able to operate under very low pressures of CO_2_ (balloon pressure), and (iii) how subtle changes in metal and halide combinations can allow for the development of single-component catalyst systems. This work is intended to inspire further research into Lewis acid catalysis using the heavier group 13 elements as a result of the complementary experimental and the in-depth mechanistic understanding provided.

## Experimental Section

### General Considerations

All solvents and reagents were purchased from Fisher Scientific or CymitQuimica and were used without further purification. The ligand, **H**_**2**_**L**, was prepared according to a previously published procedure.^23^ All synthetic manipulations were performed in the absence of air and moisture by the use of standard Schlenk techniques and an MBraun glovebox with an argon atmosphere. ^1^H, ^13^C{^1^H}, and COSY and ^1^H–^13^C{^1^H} HSQC NMR spectra were recorded on a Bruker AV400 spectrometer in CDCl_3_ or DMSO-*d*_6_ and referenced to the residual solvent peak at 7.26/2.50 ppm (^1^H) or 77.16/39.52 ppm (^13^C), respectively. High-resolution mass spectrometry analysis was performed by the Laboratorio de Técnicas Instrumentales (LTI) at the Universidad de Valladolid. High-pressure catalytic reactions (8.0 bar) were performed in Berghof High-pressure reactors (BR-40, PTFE liner, 70 mL volume) using high-purity carbon dioxide (>99.995%) purchased from Linde (no further purification) with an initial starting pressure of 8.0 bar. Low-pressure catalytic reactions (balloon pressure) were performed using a balloon and a 25 mL Schlenk tube.

### Synthesis of AlL-Cl

The ligand, **H**_**2**_**L** (500 mg, 1.33 mmol), was dissolved in 40 mL of THF in a Schlenk tube under argon. To the solution, 1.5 mL (1.35 mol, 1.0 equiv, 0.9 M in heptane) of dimethylaluminum chloride (AlMe_2_Cl) was slowly added. The reaction was stirred overnight. The reaction mixture was then concentrated to a volume of approximately 6.0 mL under reduced pressure. To this reaction mixture was then added 20 mL of hexane, and the precipitate formed was filtered and dried under vacuum to provide an analytically pure compound as a white powder (555 mg, 96%). Crystals suitable for X-ray crystallography studies were obtained by the slow diffusion of hexane into a concentrated THF solution of the complex. ^1^H NMR (400 MHz, 298 K, DMSO-*d*_6_) δ 8.39 (d, 1H, *J* = 6.5 Hz, C*H*^Py6^), 7.71 (ddd, 1H, *J* = 6.5, 6.5, and 1.2 Hz, C*H*^Py4^), 7.27 (dd, 1H, *J* = 6.5 and 6.5 Hz, C*H*^Py5^), 7.06 (d, 1H, *J* = 6.5 Hz, C*H*^Py3^), 6.60–6.54 (m, 4H, Ar*H*^3,5^), 4.14–4.00 (m, 2H, C*H*_2_^Ar^), 3.97 (s, 2H, C*H*_2_^Py^), 3.62 (d, 2H, *J* = 12.5 Hz, C*H*_2_^Ar^), 2.03 (s, 6H, Me^*ortho*^), 1.96 (s, 6H, Me^*para*^) ppm. ^13^C{^1^H} NMR (101 MHz, 298 K, DMSO-*d*_6_) δ 157.05 (*C*^Ar1^), 152.91 (*C*^Py2^), 144.24 (*C*^Py6^), 139.74 (*C*^Py4^), 130.57 (*C*^Ar5^), 127.04 (*C*^Ar3^), 125.54 (*C*^Ar4^), 123.50 (*C*^Py5^), 122.69 (*C*^Ar6^), 121.04 (*C*^Py3^), 120.59 (*C*^Ar2^), 61.26 (*C*H_2_^Ar^), 58.30 (*C*H_2_^Py^), 19.98 (Me^*ortho*^), 16.76 (Me^*para*^) ppm. HRMS (ESI+) *m*/*z* calc. for C_23_H_26_AlN_2_O_2_ ([M-Cl]^+^): 401.1804, obtained = 401.1817. Elem. Anal. Calcd (%) for C_24_H_26_AlClN_2_O_2_: C, 65.98; H, 6.00; N, 6.41. Found: C, 65.64; H, 6.23; N, 6.04.

### General Synthesis of all Other ML-X Complexes

The ligand, **H**_**2**_**L** (1.0 equiv), was dissolved in 20 mL of THF in a Schlenk tube under argon. Potassium *bis*(trimethylsilyl)amide (KHMDS; 2.0 equiv) or potassium hydride (KH; 2.2 equiv) was dissolved/suspended in 20 mL of THF in another Schlenk tube, and to this solution was added the solution of the ligand. The reaction was then stirred overnight. To the solution was added the group 13 trihalide (MX_3_; 1.0 equiv) previously dissolved in 10 mL of THF in another Schlenk tube, and the reaction was then stirred overnight. The precipitate which formed was filtered and dried under vacuum. The resulting solid was dissolved in DCM and filtered to remove any salts arising from the salt metathesis reaction. The dichloromethane was then removed, and the resulting solid was washed with hexane (15 mL), providing the final analytically pure compound as a white powder.

### AlL-Br

Following the general procedure above starting from 300 mg (0.79 mmol) of **H**_**2**_**L**, using 70.6 mg (1.76 mmol, 2.2 equiv) of KH and 213 mg (0.79 mmol, 1.0 equiv) of aluminum bromide (AlBr_3_), yielded **AlL-Br** as a white powder (120 mg, 32%). Note: this compound readily decomposed in deuterated solvent, which was not completely anhydrous. ^1^H NMR (400 MHz, 298 K, DMSO-*d*_6_) δ 8.39 (d, 1H, *J* = 6.5 Hz, C*H*^Py6^), 7.71 (dd, 1H, *J* = 6.5 and 1.2 Hz, C*H*^Py4^), 7.28 (dd, 1H, *J* = 6.5 and 6.5 Hz, C*H*^Py5^), 7.06 (d, 1H, *J* = 6.5 Hz, C*H*^Py3^), 6.62–6.52 (m, 4H, Ar*H*^3,5^), 4.18–4.01 (m, 2H, C*H*_2_^Ar^), 3.97 (s, 2H, C*H*_2_^Py^), 3.63 (d, 2H, *J* = 12.5 Hz, C*H*_2_^Ar^), 2.03 (s, 6H, Me^*ortho*^), 1.96 (s, 6H, Me^*para*^) ppm. ^13^C{^1^H} NMR (101 MHz, 298 K, DMSO-*d*_6_) δ 157.01 (*C*^Ar1^), 152.88 (*C*^Py2^), 144.19 (*C*^Py6^), 139.68 (*C*^Py4^), 130.53 (*C*^Ar5^), 126.99 (*C*^Ar3^), 125.50 (*C*^Ar4^), 123.46 (*C*^Py5^), 122.66 (*C*^Ar6^), 121.00 (*C*^Py3^), 120.55 (*C*^Ar2^), 61.23 (*C*H_2_^Ar^), 58.24 (*C*H_2_^Py^), 19.92 (Me^*ortho*^), 16.69 (Me^*para*^) ppm. HRMS (ESI+) *m*/*z* calc. for C_23_H_26_AlN_2_O_2_ ([M-Br]^+^): 401.1804, obtained = 401.1813. Elem. Anal. Calcd (%) for C_24_H_26_AlBrN_2_O_2_: C, 59.88; H, 5.44; N, 5.82. Found: C, 59.46; H, 5.86; N, 5.67.

### GaL-Cl

Following the general procedure above starting from 500 mg (1.33 mmol) of **H**_**2**_**L**, using 558 mg (2.66 mmol, 2.0 equiv) of KHMDS and 234 mg (1.33 mmol, 1.0 equiv) of gallium chloride (GaCl_3_), yielded **GaL-Cl** as a white powder (395 mg, 62%). Crystals suitable for X-ray crystallography studies were obtained by the slow diffusion of hexane into a concentrated THF solution of the complex. ^1^H NMR (400 MHz, 298 K, DMSO-*d*_6_) δ 8.40 (d, 1H, *J* = 6.5 Hz, C*H*^Py6^), 7.78 (dd, 1H, *J* = 6.5 and 1.2 Hz, C*H*^Py4^), 7.33 (dd, 1H, *J* = 6.5 and 6.5 Hz, C*H*^Py5^), 7.14 (d, 1H, *J* = 6.5 Hz, C*H*^Py3^), 6.62–6.52 (m, 4H, Ar*H*^3,5^), 4.08 (s, 2H, C*H*_2_^Py^), 4.07–4.01 (m, 2H, C*H*_2_^Ar^), 3.80 (d, 2H, *J* = 12.5 Hz, C*H*_2_^Ar^), 2.02 (s, 6H, Me^*ortho*^), 1.98 (s, 6H, Me^*para*^) ppm. ^13^C{^1^H} NMR (101 MHz, 298 K, DMSO-*d*_6_) δ 158.27 (*C*^Ar1^), 151.67 (*C*^Py2^), 143.91 (*C*^Py6^), 140.38 (*C*^Py4^), 130.81 (*C*^Ar5^), 127.76 (*C*^Ar3^), 126.23 (*C*^Ar4^), 124.07 (*C*^Py5^), 123.08 (*C*^Ar6^), 121.73 (*C*^Py3^), 120.03 (*C*^Ar2^), 60.44 (*C*H_2_^Ar^), 57.87 (*C*H_2_^Py^), 19.93 (Me^*ortho*^), 16.77 (Me^*para*^) ppm. HRMS (ESI+) *m*/*z* calc. for C_23_H_26_GaN_2_O_2_ ([M-Cl]^+^): 443.1245, obtained = 443.1260. Elem. Anal. Calcd (%) for C_24_H_26_GaClN_2_O_2_: C, 60.10; H, 5.46; N, 5.84. Found: C, 60.19; H, 5.26; N, 5.92.

### GaL-Br

Following the general procedure above starting from 300 mg (0.79 mmol) of **H**_**2**_**L**, using 319 mg (1.59 mmol, 2.0 equiv) of KHMDS and 247 mg (0.80 mmol, 1.0 equiv) of gallium bromide (GaBr_3_), yielded **GaL-Br** as a white powder (395 mg, 64%). ^1^H NMR (400 MHz, 298 K, DMSO-*d*_6_) δ 8.39 (d, 1H, *J* = 6.5 Hz, C*H*^Py6^), 7.79 (ddd, 1H, *J* = 6.5, 6.5, and 1.2 Hz, C*H*^Py4^), 7.34 (dd, 1H, *J* = 6.5 and 6.5 Hz, C*H*^Py5^), 7.16 (d, 1H, *J* = 6.5 Hz, C*H*^Py3^), 6.63–6.53 (m, 4H, Ar*H*^3,5^), 4.08 (s, 2H, C*H*_2_^Py^), 4.07–4.00 (m, 2H, C*H*_2_^Ar^), 3.80 (d, 2H, *J* = 12.5 Hz, C*H*_2_^Ar^), 2.02 (s, 6H, Me^*ortho*^), 1.99 (s, 6H, Me^*ortho*^) ppm. ^13^C{^1^H} NMR (101 MHz, 298 K, DMSO-*d*_6_) δ 158.20 (*C*^Ar1^), 151.65 (*C*^Py2^), 143.86(*C*^Py6^), 140.44 (*C*^Py4^), 130.85 (*C*^Ar5^), 127.79 (*C*^Ar3^), 126.23 (*C*^Ar4^), 124.12 (*C*^Py5^), 123.16 (*C*^Ar6^), 121.75 (*C*^Py3^), 120.02 (*C*^Ar2^), 60.41 (*C*H_2_^Ar^), 57.92 (*C*H_2_^Py^), 19.93 (Me^*ortho*^), 16.79 (Me^*para*^) ppm. HRMS (ESI+) *m*/*z* calc. for C_23_H_26_GaN_2_O_2_ ([M-Br^+^): 443.1245, obtained = 443.1257. Elem. Anal. Calcd (%) for C_24_H_26_GaBrN_2_O_2_: C, 55.00; H, 5.00; N, 5.35. Found: C, 54.81; H, 5.24; N, 5.56.

### GaL-I

Following the general procedure above starting from 500 mg (1.33 mmol) of **H**_**2**_**L**, using 117 mg (2.92 mmol, 2.2 equiv) of KH and 598 mg (0.79 mmol, 1.0 equiv) of gallium iodide (GaI_3_), yielded **GaL-I** as a white powder (446 mg, 59%). ^1^H NMR (400 MHz, 298 K, DMSO-*d*_6_) δ 8.38 (d, 1H, *J* = 6.5 Hz, C*H*^Py6^), 7.79 (dd, 1H, *J* = 6.5 and 1.2 Hz, C*H*^Py4^), 7.34 (dd, 1H, *J* = 6.5 and 6.5 Hz, C*H*^Py5^), 7.16 (d, 1H, *J* = 6.5 Hz, C*H*^Py3^), 6.62–6.52 (m, 4H, Ar*H*^3,5^), 4.09 (s, 2H, C*H*_2_^Py^), 4.08–4.00 (m, 2H, C*H*_2_^Ar^), 3.81 (d, 2H, *J* = 12.5 Hz, C*H*_2_^Ar^), 2.02 (s, 6H, Me^*ortho*^), 1.99 (s, 6H, Me^*para*^) ppm. ^13^C{^1^H} NMR (101 MHz, 298 K, DMSO-*d*_6_) δ 158.17 (*C*^Ar1^), 151.60 (*C*^Py2^), 143.84 (*C*^Py6^), 140.39 (*C*^Py4^), 130.81 (*C*^Ar5^), 127.75 (*C*^Ar3^), 126.19 (*C*^Ar4^), 124.09 (*C*^Py5^), 123.14 (*C*^Ar6^), 121.71 (*C*^Py3^), 119.98 (*C*^Ar2^), 60.37 (*C*H_2_^Ar^), 57.93 (*C*H_2_^Py^), 19.87 (Me^*ortho*^), 16.74 (Me^*para*^) ppm. HRMS (ESI+) *m*/*z* calc. for C_23_H_26_GaN_2_O_2_ ([M-I]^+^): 443.1245, obtained = 443.1259. Elem. Anal. Calcd (%) for C_24_H_26_GaIN_2_O_2_: C, 50.47; H, 4.59; N, 4.91. Found: C, 50.26; H, 4.38; N, 4.91.

### InL-Cl

Following the general procedure above starting from 500 mg (1.33 mmol) of **H**_**2**_**L**, using 117 mg (2.92 mmol, 2.2 equiv) of KH and 300 mg (1.33 mmol, 1.0 equiv) of indium chloride (InCl_3_), yielded **InL-Cl** as a white powder (369 mg, 53%). Crystals suitable for X-ray crystallography studies were obtained by the storage of a concentrated THF solution of the complex at −20 °C. ^1^H NMR (400 MHz, 298 K, DMSO-*d*_6_) δ 8.48 (d, 1H, *J* = 6.5 Hz, C*H*^Py6^), 7.70 (dd, 1H, *J* = 6.5 and 1.2 Hz, C*H*^Py4^), 7.27 (dd, 1H, *J* = 6.5 and 6.5 Hz, C*H*^Py5^), 6.98 (d, 1H, *J* = 6.5 Hz, C*H*^Py3^), 6.58–6.45 (m, 4H, Ar*H*^3,5^), 4.28–4.06 (m, 2H, C*H*_2_^Ar^), 3.85 (s, 2H, C*H*_2_^Py^), 3.71–3.48 (m, 2H, C*H*_2_^Ar^), 2.01 (s, 6H, Me^*ortho*^), 1.88 (s, 6H, Me^*para*^) ppm. ^13^C{^1^H} NMR (101 MHz, 298 K, DMSO-*d*_6_) δ 160.65 (*C*^Ar1^), 153.33 (*C*^Py2^), 145.02 (*C*^Py6^), 139.71 (*C*^Py4^), 130.68 (*C*^Ar5^), 128.59 (*C*^Ar3^), 126.79 (*C*^Ar4^), 123.49 (*C*^Py5^), 122.44 (*C*^Ar6^), 121.68 (*C*^Py3^), 120.58 (*C*^Ar2^), 60.81 (*C*H_2_^Ar^), 57.05 (*C*H_2_^Py^), 19.96 (Me^*ortho*^), 16.56 (Me^*para*^) ppm. HRMS (ESI+) *m*/*z* calc. for C_23_H_26_InN_2_O_2_ ([M-Cl]^+^): 489.1028, obtained = 489.1044. Elem. Anal. Calcd (%) for C_24_H_26_InClN_2_O_2_: C, 54.93; H, 4.99; N, 5.34. Found: C, 54.73; H, 4.78; N, 5.45.

### Cycloaddition of Epoxides and Carbon Dioxide

A high-pressure reactor or Schlenk tube, equipped with a stirrer bar, was charged with epoxide, catalyst, and, where necessary, *tetra*-butylammonium halide cocatalyst. In the case of the high-pressure reactions, the reactor was then filled with CO_2_ to 2.0 bar and partially vented, a procedure that was repeated 3 times, before being finally filled with CO_2_ to a pressure of 8.0 bar. Meanwhile, for the low-pressure reactions, the Schlenk tube was sealed with a septum, and a balloon of CO_2_ was introduced through the septum using a syringe and needle. The reactors/Schlenk tubes were left stirring for the required time and at the required temperature. At the end of the reaction, the reactors/Schlenk tubes were slowly vented. An aliquot of the crude reaction mixture was added to CDCl_3_, and the yield and conversion were calculated by integration of the known peaks. In all cases, only the epoxide and cyclic carbonate products were observed with no evidence of other side products.

### Single-Crystal X-ray Diffraction

Diffraction data were collected using an Oxford Diffraction Supernova diffractometer equipped with an Atlas CCD area detector and a four-circle kappa goniometer. For the data collection, a Mo source with multilayer optics was used. Data integration, scaling, and empirical absorption correction were carried out using the CrysAlis Program package.^24^ The structures were solved using direct methods and refined by Full-Matrix-Least-Squares against F2 with SHELX^25^ under OLEX2.^26^ The non-hydrogen atoms were refined anisotropically, and hydrogen atoms were placed at idealized positions and refined using the riding model. Full-matrix least-squares refinements were carried out by minimizing Σ*w*(*Fo*2 – *Fc*2)2 with the SHELXL weighting scheme and stopped at shift/err <0.001. The final residual electron density maps showed no remarkable features. Graphics were made with OLEX2 and MERCURY.^27^ The structures reported in this paper have been deposited with the Cambridge Crystallographic Data Centre (CCDC) as supplementary publication numbers: 2345917 (**AlL-Cl**), 2338178 (**GaL-Cl**), and 2345889 (**InL-Cl**). It should be noted that the structure of complex **AlL-Cl** is affected by a disorder which could not be satisfactorily modeled. However, despite Alert A, the refinement parameters are of a high enough quality to demonstrate without any doubt the connectivity of the molecule concerned, which was the purpose of the determination. Some of the alerts arise from disorder in the free THF molecule present in the unit cell.

### Computational Study Information

All DFT calculations were performed using ORCA 4.2.1 computational software.^28^ Solvation optimizations and analytical frequency calculations were performed at the RI-B97-D3/def2-TZVP level of theory.^29−31^ Final single-point energies and solvation corrections were calculated at the RIJCOSX-ωB97M-V/def2-TZVPP level of theory.^31,32^ All solvation corrections were calculated using the SMD model with a parameters for hexan-1-ol,^33^ which has previously been shown to be a good solvation approximation for an epoxide solvent environment.^34^ Analytical frequencies were calculated for inclusion of the zero point energy correction and entropic contributions to the free energy term as well as confirming all intermediates were true with no imaginary modes and all transition states had the correct critical frequency of decomposition. Numerical precision integration grids were increased beyond the default settings to Grid4 for the SCF step and Grid5 for the final energy evaluation. Concentration correction, to account for the low catalyst loading and substrate/solvent environment, was applied as a free energy correction based on the Van’t Hoff reaction quotient equation *RT* ln (*Q*),^35^ where *Q* accounts for the concentration gradient between the substrate and the catalyst. Graphical visualization and structural analysis performed from the DFT calculations using Avogadro 1.2.0.^36^

## Supporting Information Available

Original spectroscopic data (NMR and IR spectra), detailed crystallographic data, and computational study information and data (PDF)
