# Supplementary material for: Binary and Halide-free Catalyst Systems Based on Al/Ga/In Aminopyridylbisphenolate Complexes for the Cycloaddition of Epoxides and CO2
Source: Inorg Chem. 2024 Aug 2;63(33):15376–87. doi: 10.1021/acs.inorgchem.4c02352 (PMC11337169; doi:10.1021/acs.inorgchem.4c02352)
Supplement: Supplementary file 1 — ic4c02352_si_001.pdf [file ic4c02352_si_001.pdf]

Supporting Information for:

# Binary and Halide-free Catalyst Systems Based on Al/Ga/In Aminopyridylbisphenolate Complexes for the Cycloaddition of Epoxides and $CO_2$

*Jesús Damián Burgoa,*[†] *Lucía Álvarez-Miguel,*[†] *Marta E. G. Mosquera,*[†*] *Alex Hamilton,*[‡*] *Christopher J. Whiteoak*[†*]

†Universidad de Alcalá, Grupo SOSCATCOM, Departamento de Química Orgánica y Química Inorgánica, Facultad de Farmacia and Instituto de Investigación Química Andrés M. del Río (IQAR), Campus Universitario, Ctra. Madrid-Barcelona Km. 33,600, 28871 Alcalá de Henares, Madrid, Spain.

‡Sheffield Hallam University, Biomolecular Sciences Research Centre (BMRC) and Department of Biosciences and Chemistry, College of Health, Wellbeing and Life Sciences Howard Street, Sheffield, S1 1WB, United Kingdom.

# Contents:

| | *Page* |
|---|---|
| **[1]** General experimental considerations | S1 |
| **[2]** Original NMR and IR spectra for the complexes | S1 |
| **[3]** Instability of compound **AlL-Br** | S19 |
| **[4]** X-ray crystallography data for **AlL-Cl**, **GaL-Cl** and **InL-Cl** | S20 |
| **[5]** Computational study details | S21 |
| **[6]** Computational study data | S22 |
| **[7]** References | S69 |

## [1] General experimental considerations

All solvents and reagents were purchased from Fisher Scientific or Cymit Quimica and were used without further purification. The ligand, **$H_2$L**, was prepared according to a previously published procedure.[1] All synthetic manipulations were performed in the absence of air and moisture by the use of standard Schlenk techniques and an MBraun glove box with an argon atmosphere. $^1$H, $^{13}$C{$^1$H}, COSY and $^1$H-$^{13}$C{$^1$H} HSQC NMR spectra were recorded on a Bruker AV400 spectrometer in $CDCl_3$ or DMSO-d$^6$ and referenced to the residual solvent peak at 7.26/2.50 ppm ($^1$H) or 77.16/39.52 ppm ($^{13}$C), respectively. High resolution mass spectrometry analysis was performed by the Laboratorio de Técnicas Instrumentales (LTI) at the Universidad de Valladolid. High-pressure catalytic reactions (8.0 bar) were performed in Berghof High-pressure reactors (BR-40, PTFE liner, 70 mL volume) using high-purity carbon dioxide (>99.995 %) purchased from Linde (no further purification), with an initial starting pressure of 8.0 bar. Low-pressure catalytic reactions (balloon pressure) were performed using a balloon and a 25 mL Schlenk tube.

## [2] Original NMR and IR spectra for the complexes

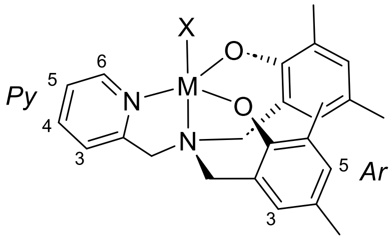


Spectra for **AlL-Cl**

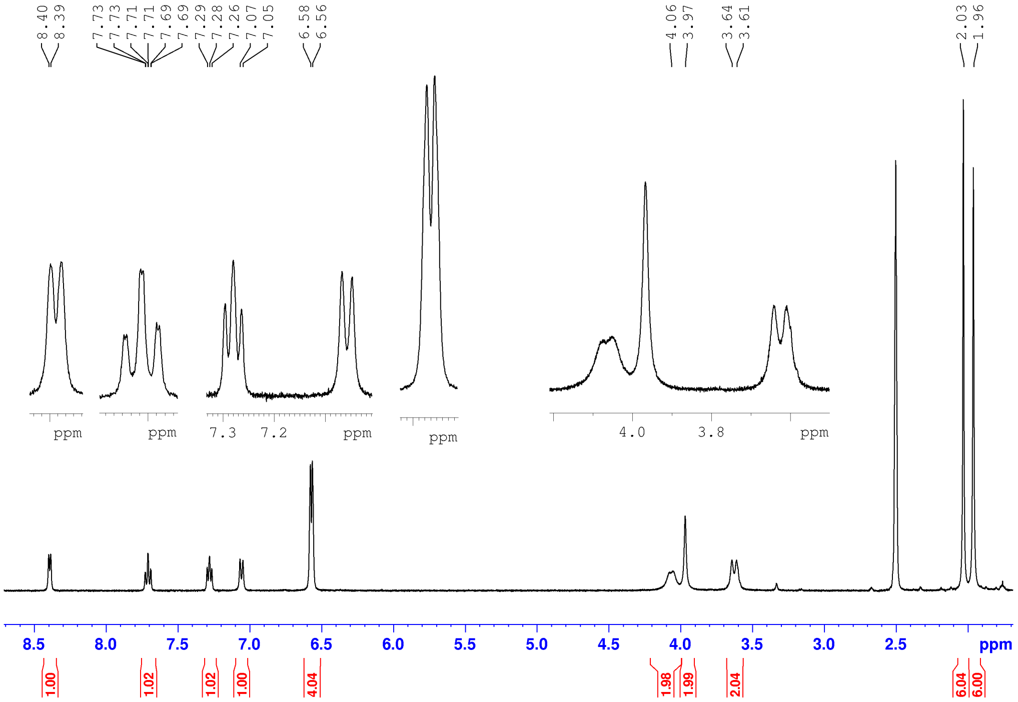


**Figure S1.** $^1$H NMR spectrum of compound **AlL-Cl** in DMSO-d$^6$ at 298K.

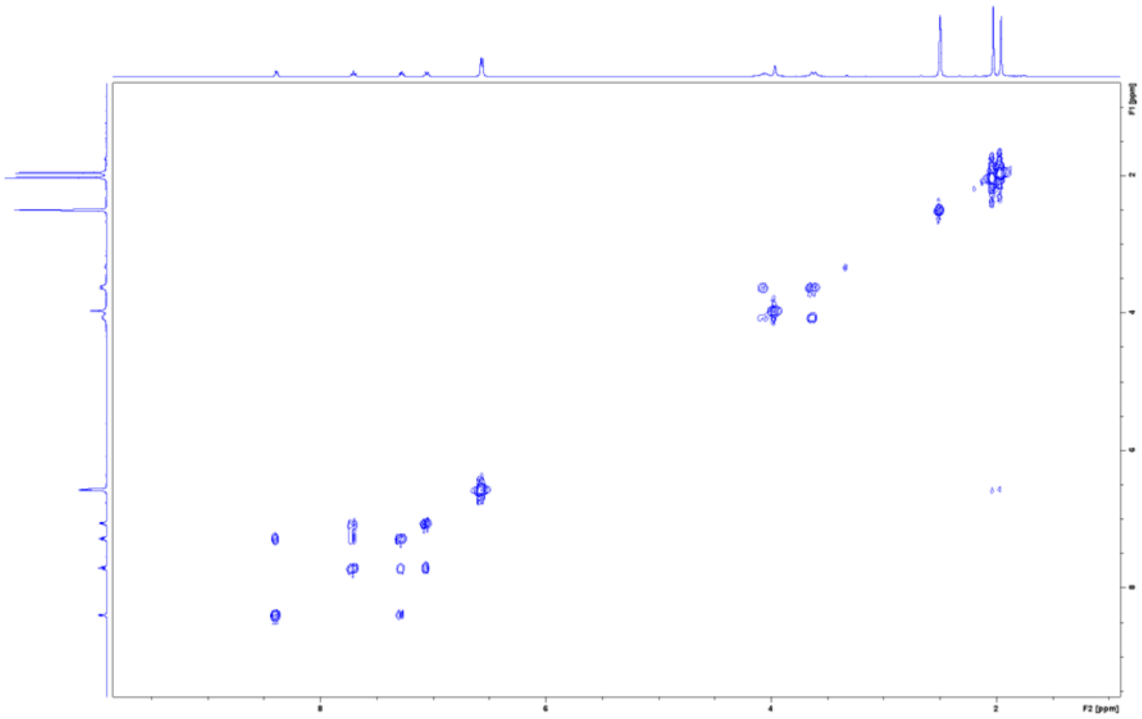

**Figure S2.** COSY NMR spectrum of compound **AlL-Cl** in DMSO-d$^6$ at 298K.

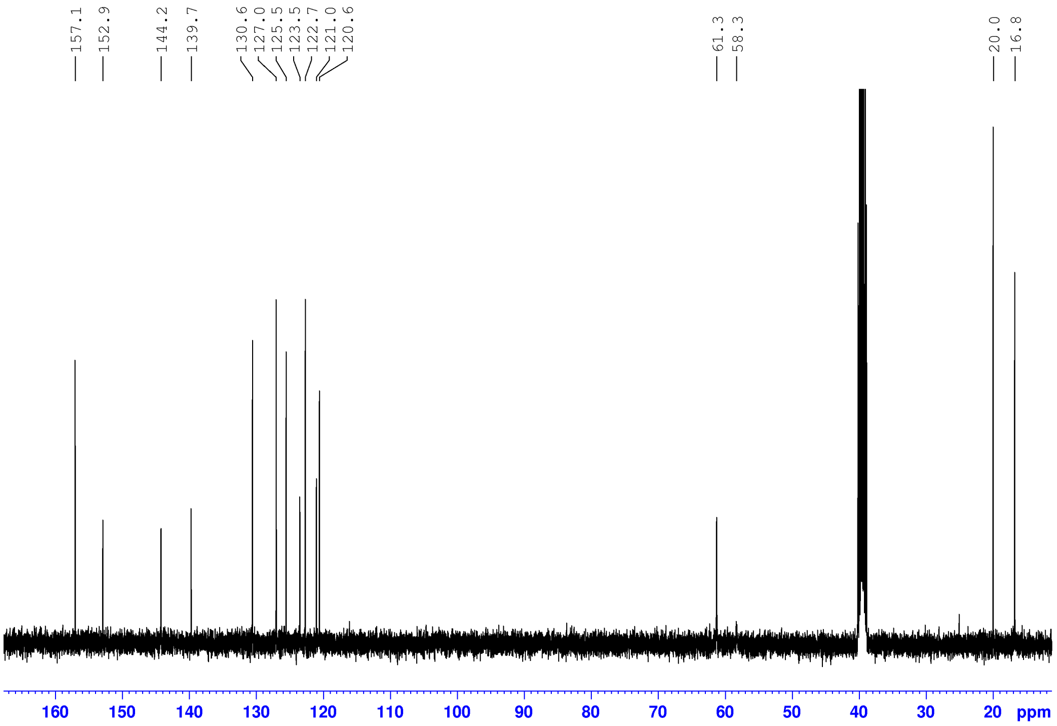


**Figure S3.** $^{13}C\{^1H\}$ NMR spectrum of compound **AlL-Cl** in DMSO-d$^6$ at 298K.

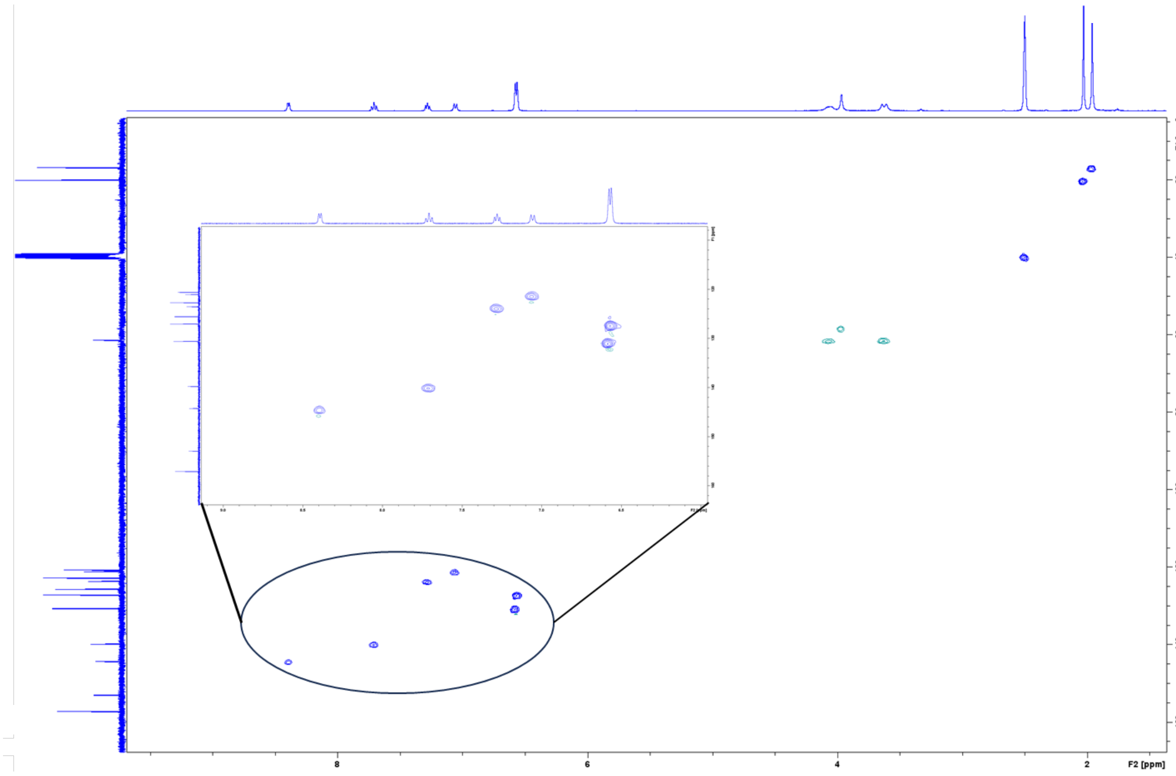


**Figure S4.** $^{1}H$-$^{13}C\{^{1}H\}$ HSQC NMR spectrum of compound **AlL-Cl** in DMSO-d$^{6}$ at 298K.

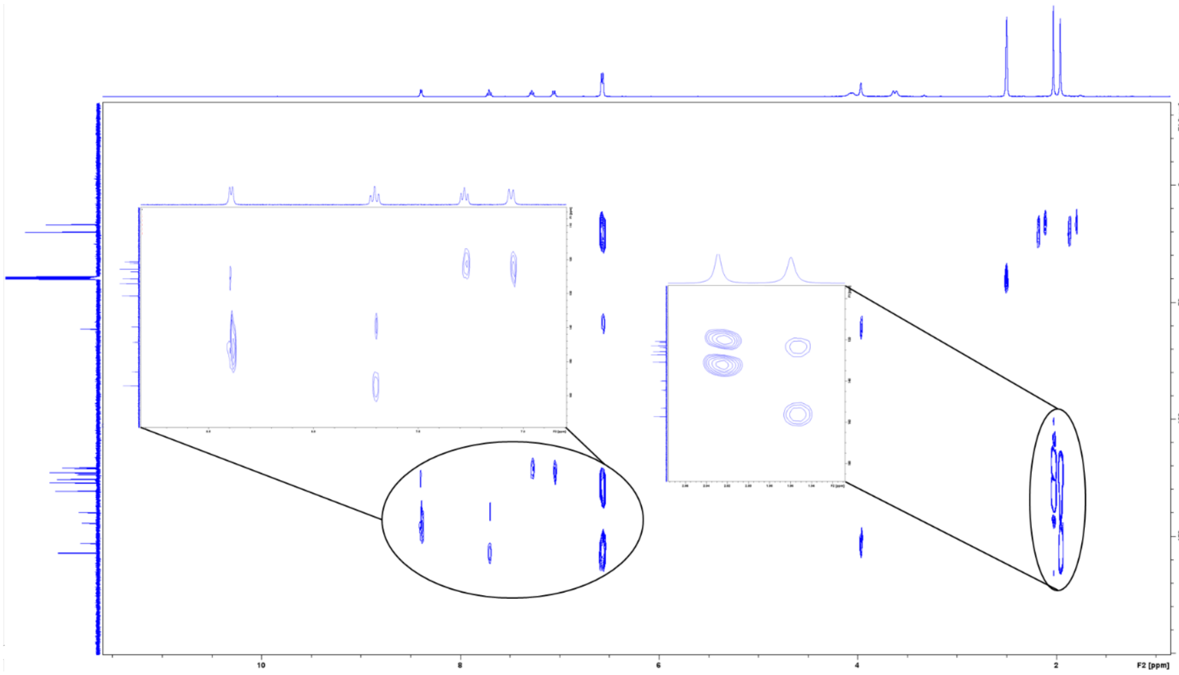


**Figure S5.** $^{1}H$-$^{13}C\{^{1}H\}$ HMBC NMR spectrum of compound **AlL-Cl** in DMSO-d$^{6}$ at 298K.

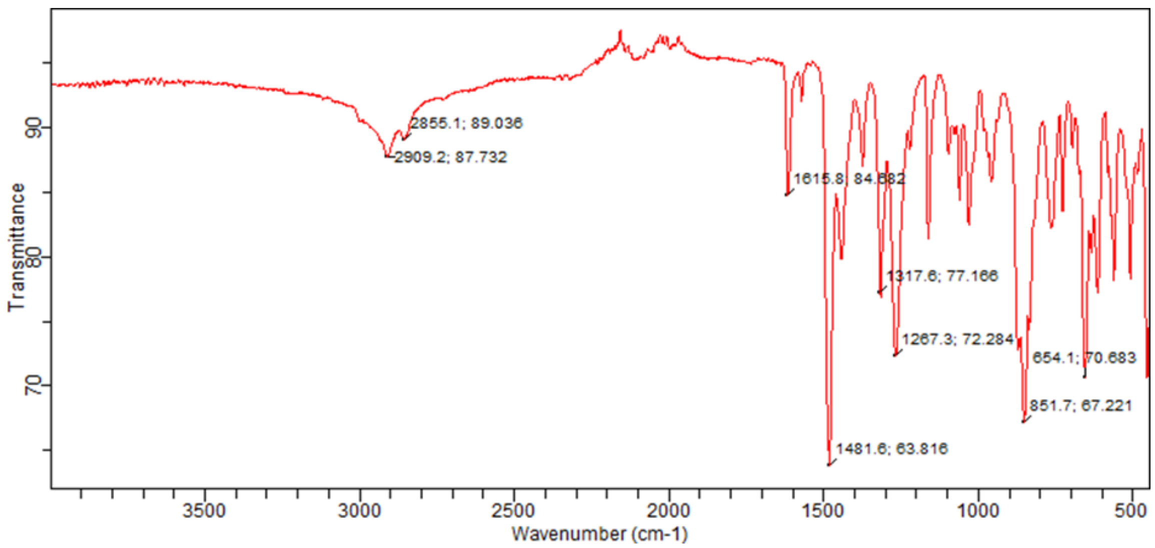


**Figure S6.** IR spectrum of compound **AlL-Cl** at 298K.

Spectra for **AlL-Br**

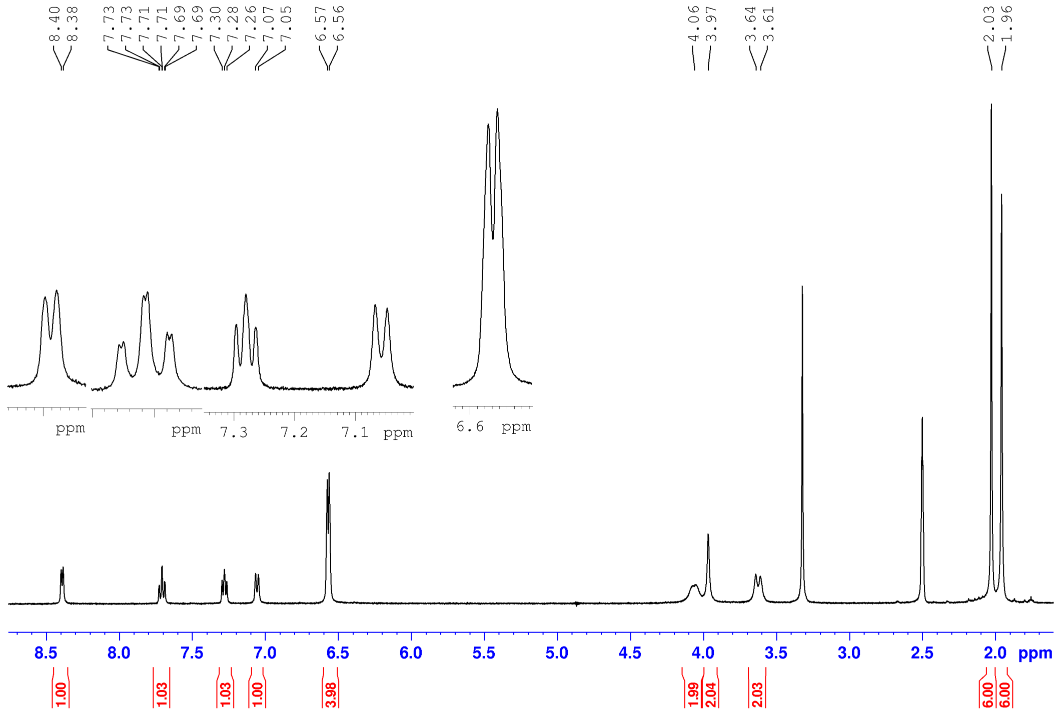

**Figure S7.** $^{1}H$ NMR spectrum of compound **AlL-Br** in DMSO-$d^6$ at 298K.

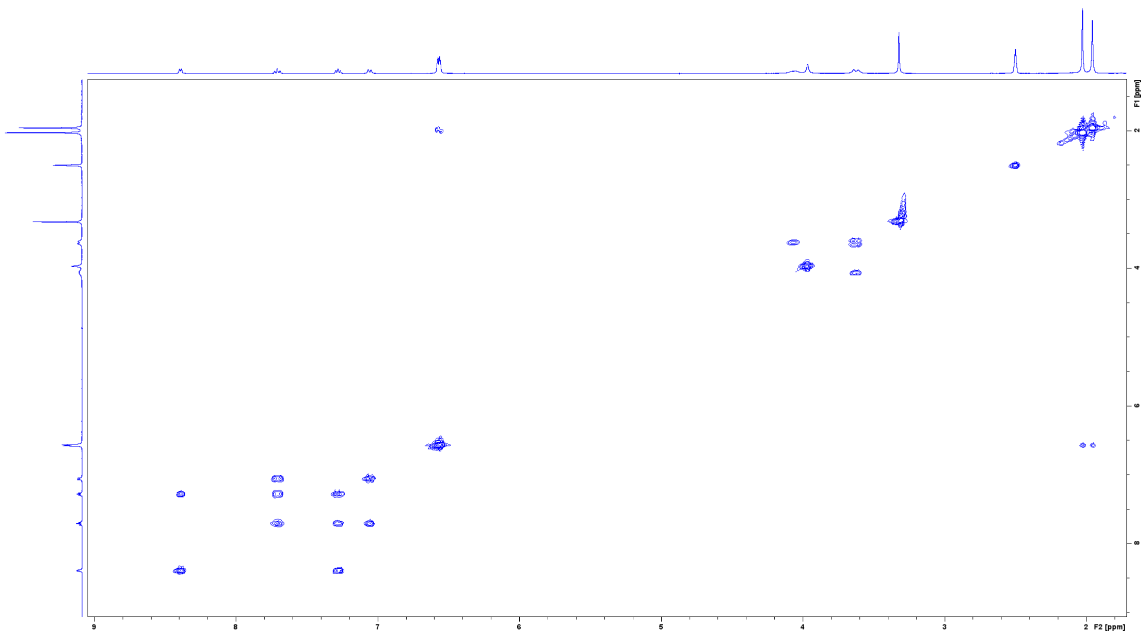

**Figure S8.** COSY NMR spectrum of compound **AlL-Br** in DMSO-d6 at 298K.

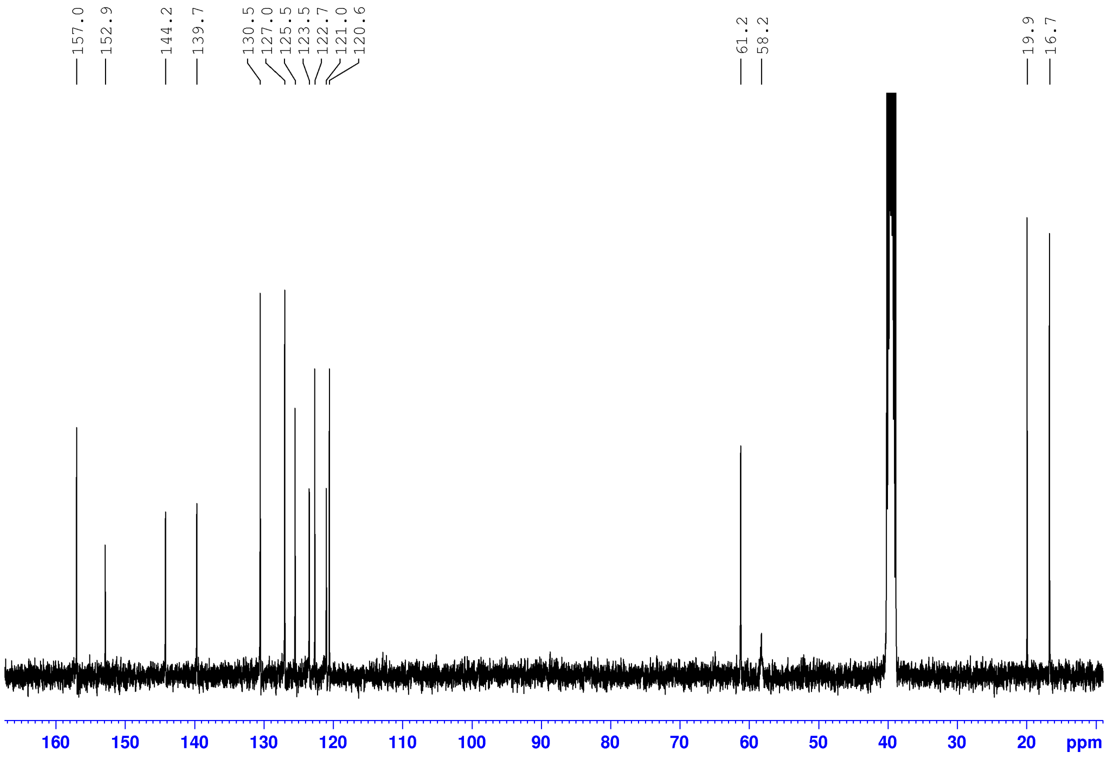


**Figure S9.** $^{13}C\{^{1}H\}$ NMR spectrum of compound **AlL-Br** in DMSO-d6 at 298K.

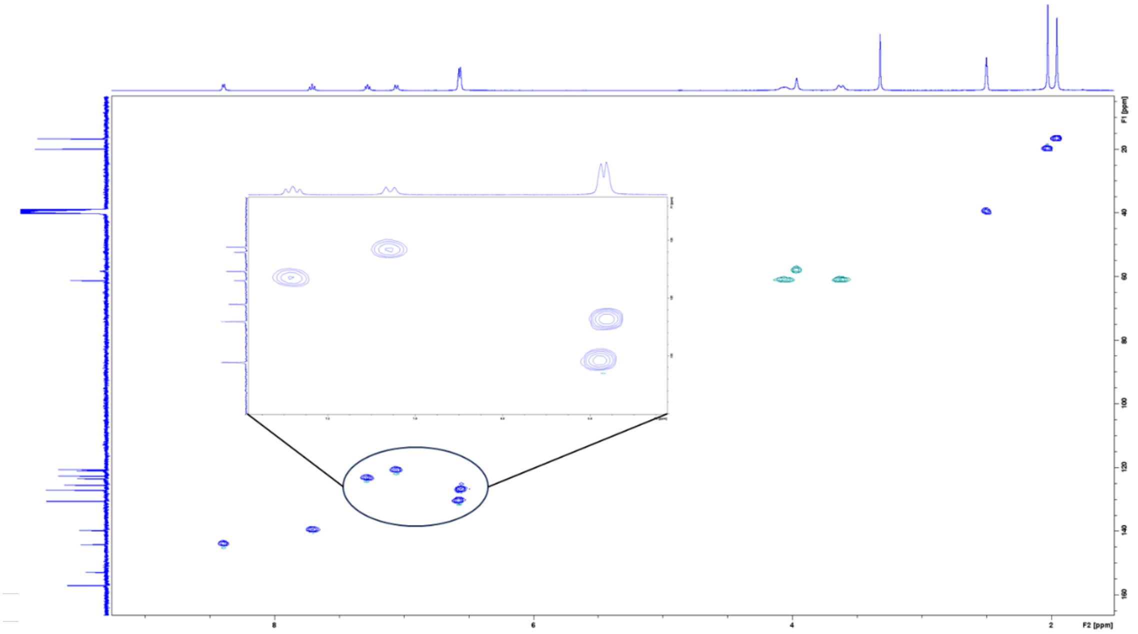


**Figure S10.** $^{1}H$-$^{13}C\{^{1}H\}$ HSQC NMR spectrum of compound **AlL-Br** in DMSO-d$^{6}$ at 298K.

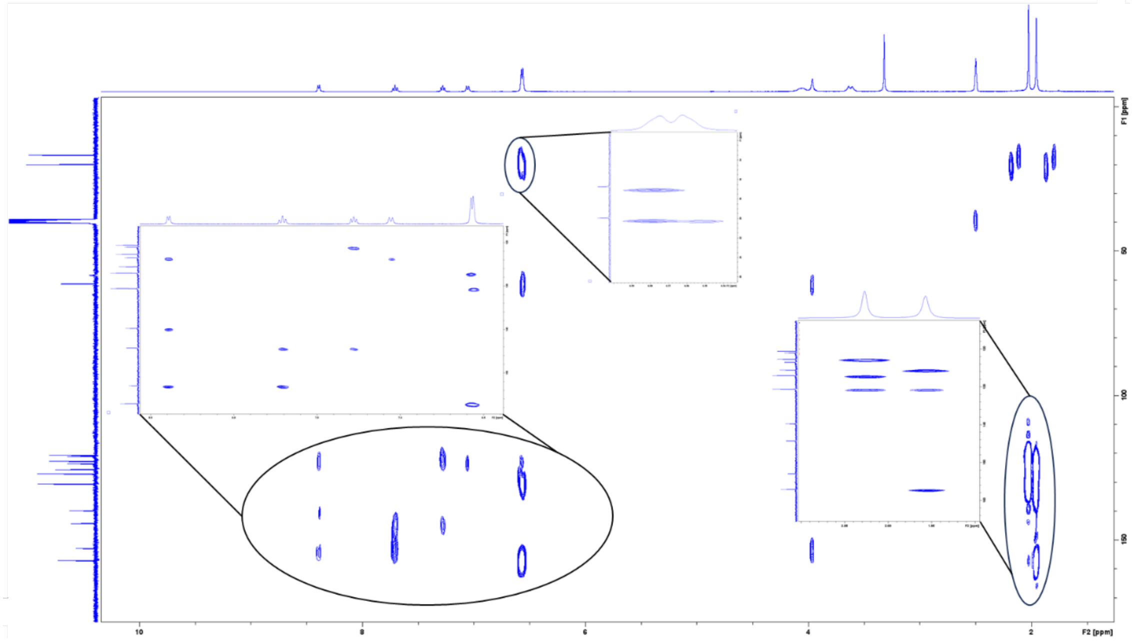


**Figure S11.** $^{1}H$-$^{13}C\{^{1}H\}$ HMBC NMR spectrum of compound **AlL-Br** in DMSO-d$^{6}$ at 298K.

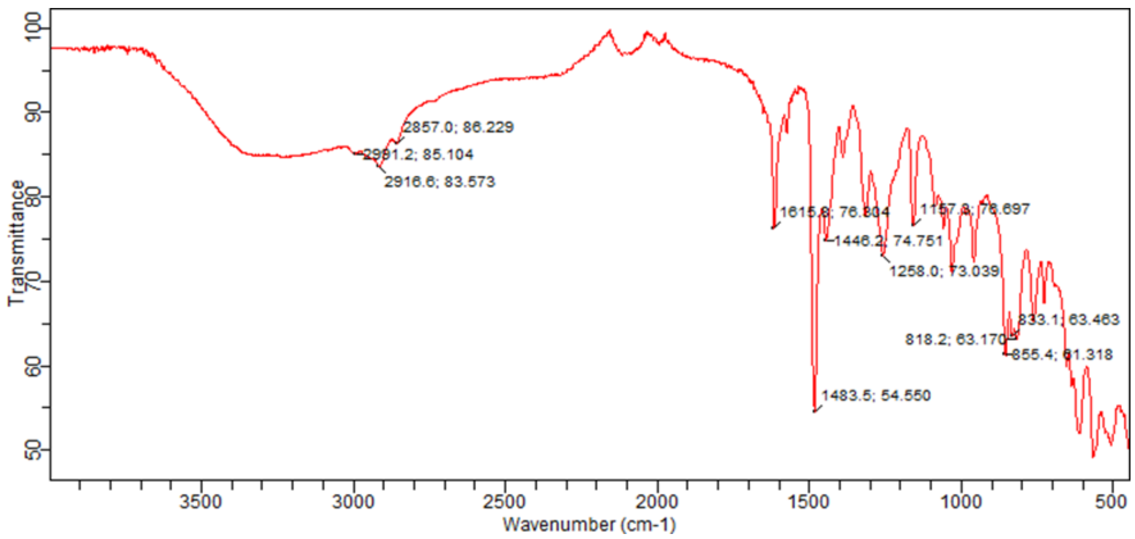


**Figure S12.** IR spectrum of compound **AlL-Br** at 298K.

Spectra for **GaL-Cl**

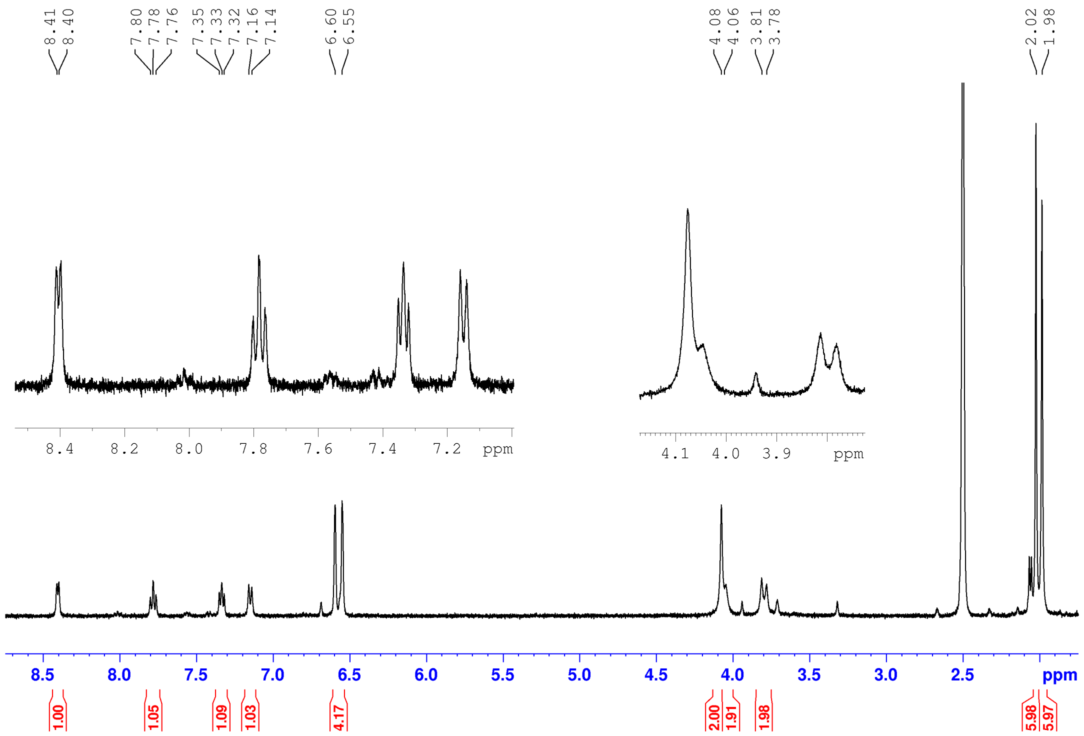

**Figure S13.** $^{1}H$ NMR spectrum of compound **GaL-Cl** in DMSO-$d^6$ at 298K.

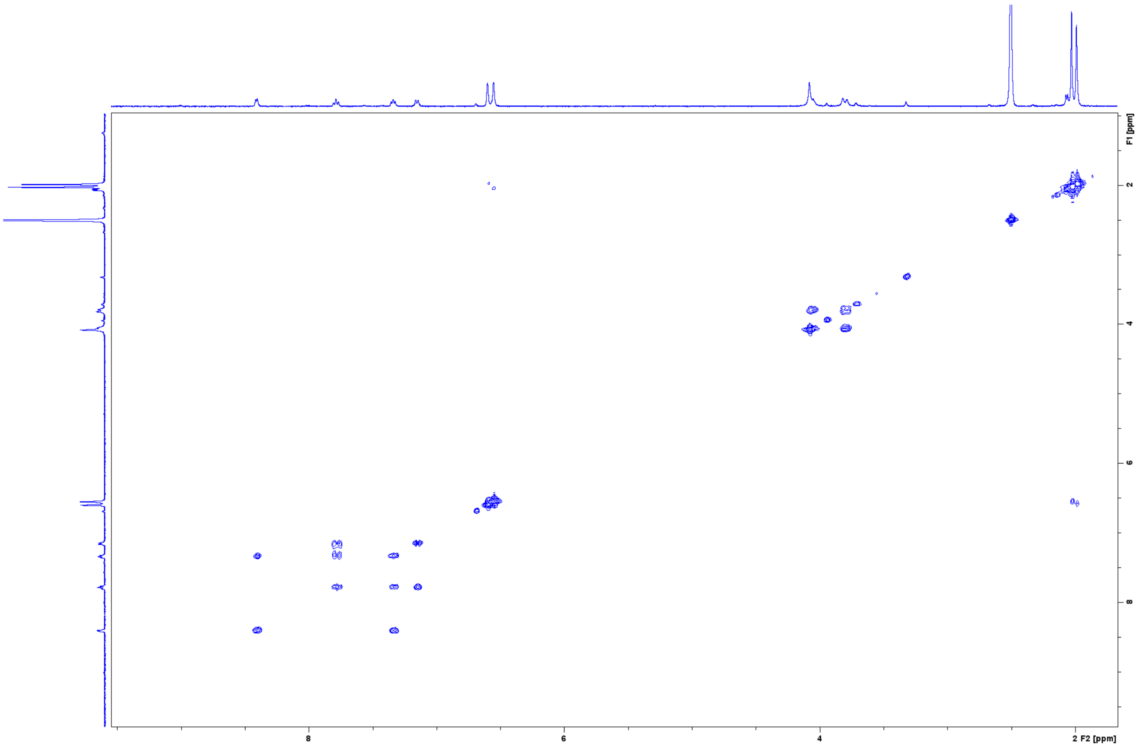


**Figure S14.** COSY NMR spectrum of compound **GaL-Cl** in DMSO-d[6] at 298K.

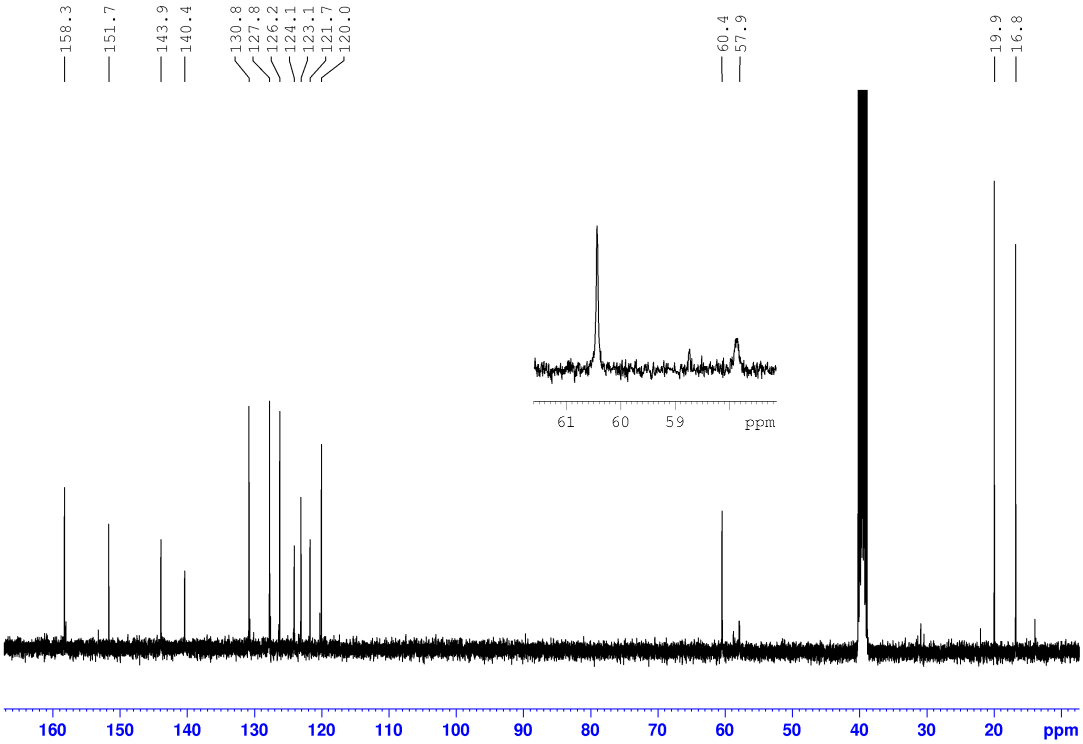


**Figure S15.** $^{13}C\{^1H\}$ NMR spectrum of compound **GaL-Cl** in DMSO-d[6] at 298K.

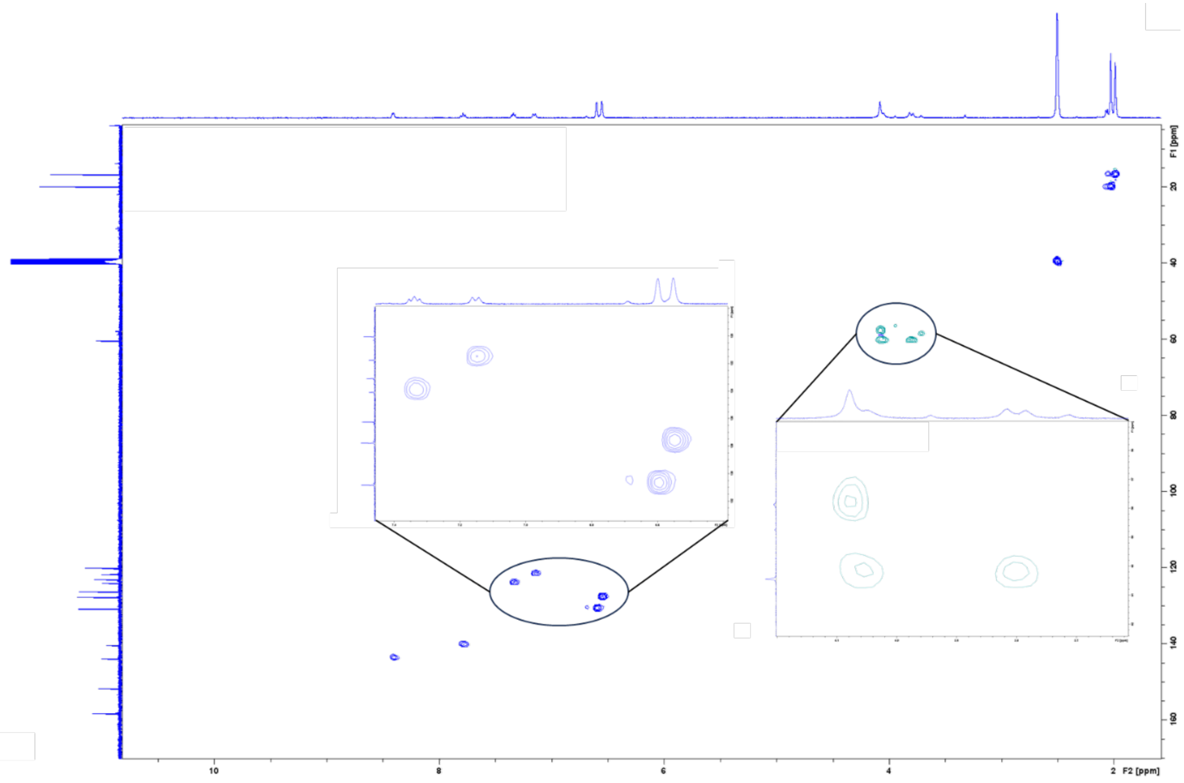

**Figure S16.** $^{1}H$-$^{13}C\{^{1}H\}$ HSQC NMR spectrum of compound **GaL-Cl** in DMSO-d$^{6}$ at 298K.

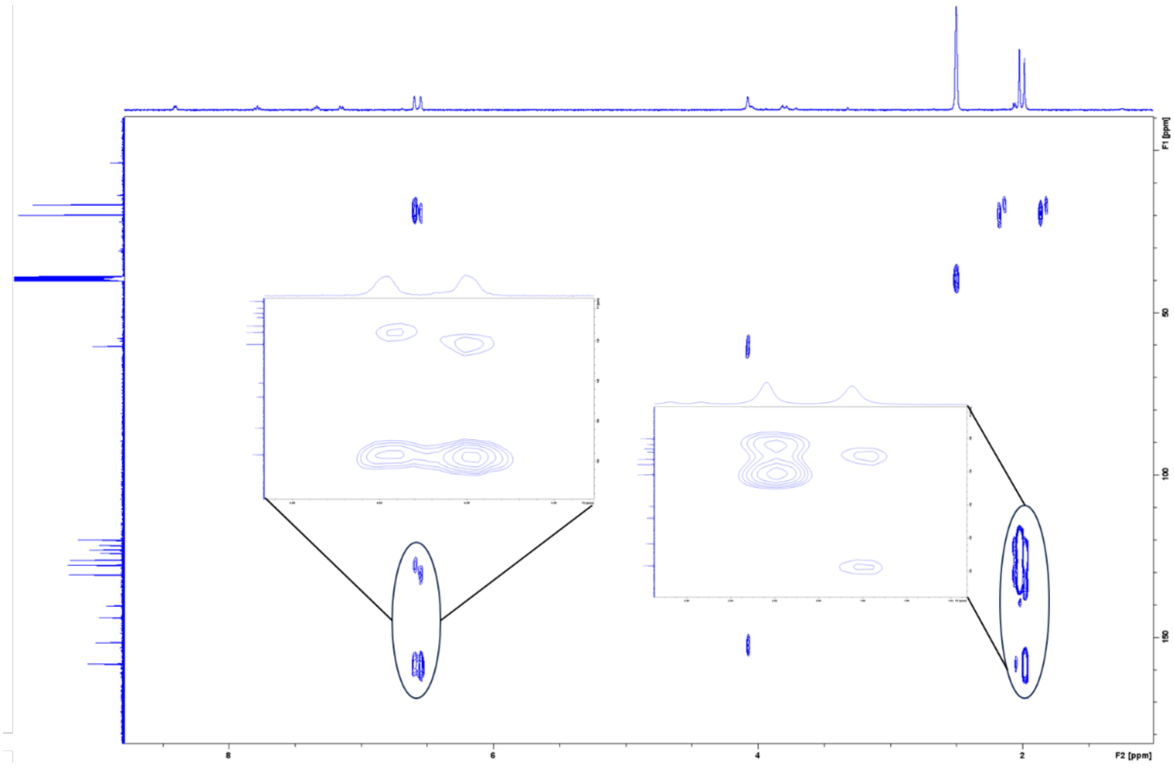

**Figure S17.** $^{1}H$-$^{13}C\{^{1}H\}$ HMBC NMR spectrum of compound **GaL-Cl** in DMSO-d$^{6}$ at 298K.

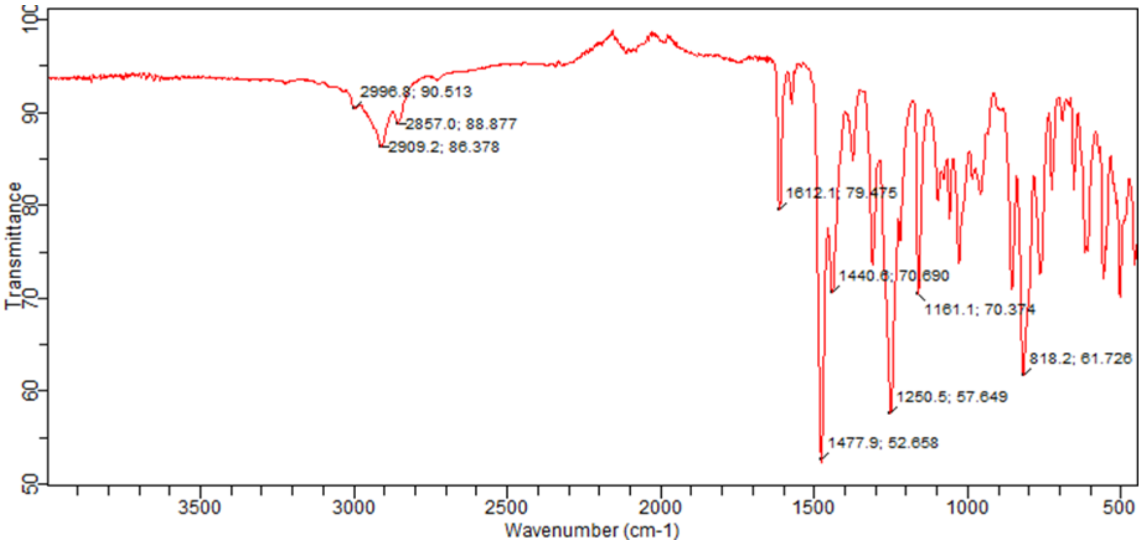


**Figure S18.** IR spectrum of compound **GaL-Cl** at 298K.

Spectra for **GaL-Br**

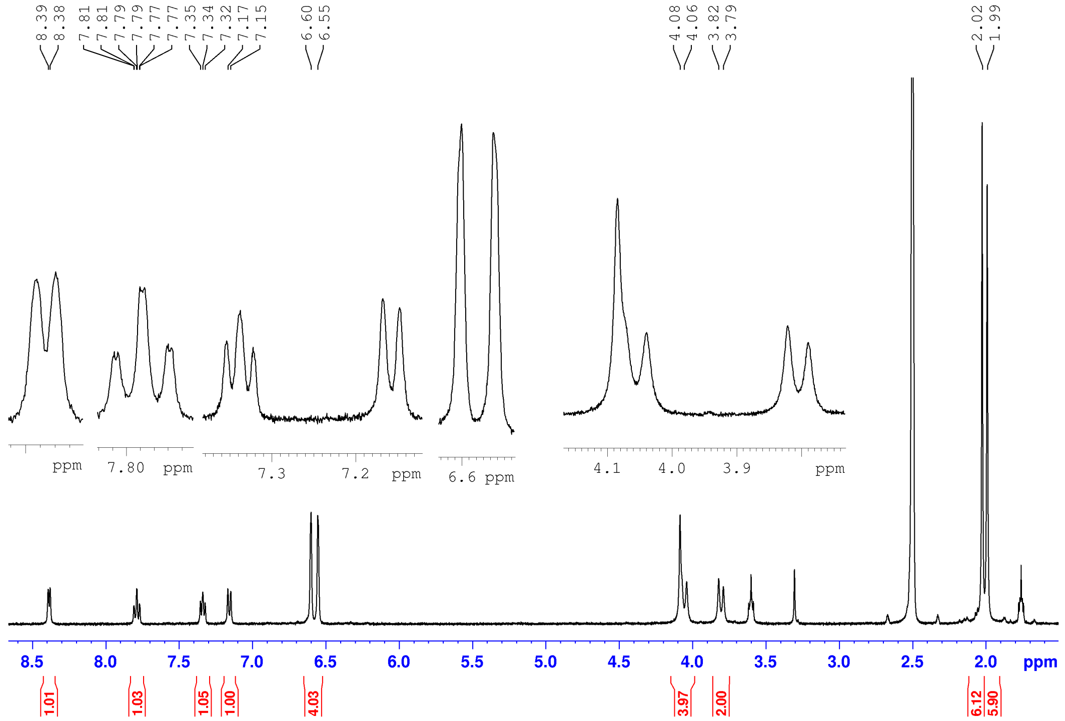


**Figure S19.** $^{1}$H NMR spectrum of compound **GaL-Br** in DMSO-d$^{6}$ at 298K.

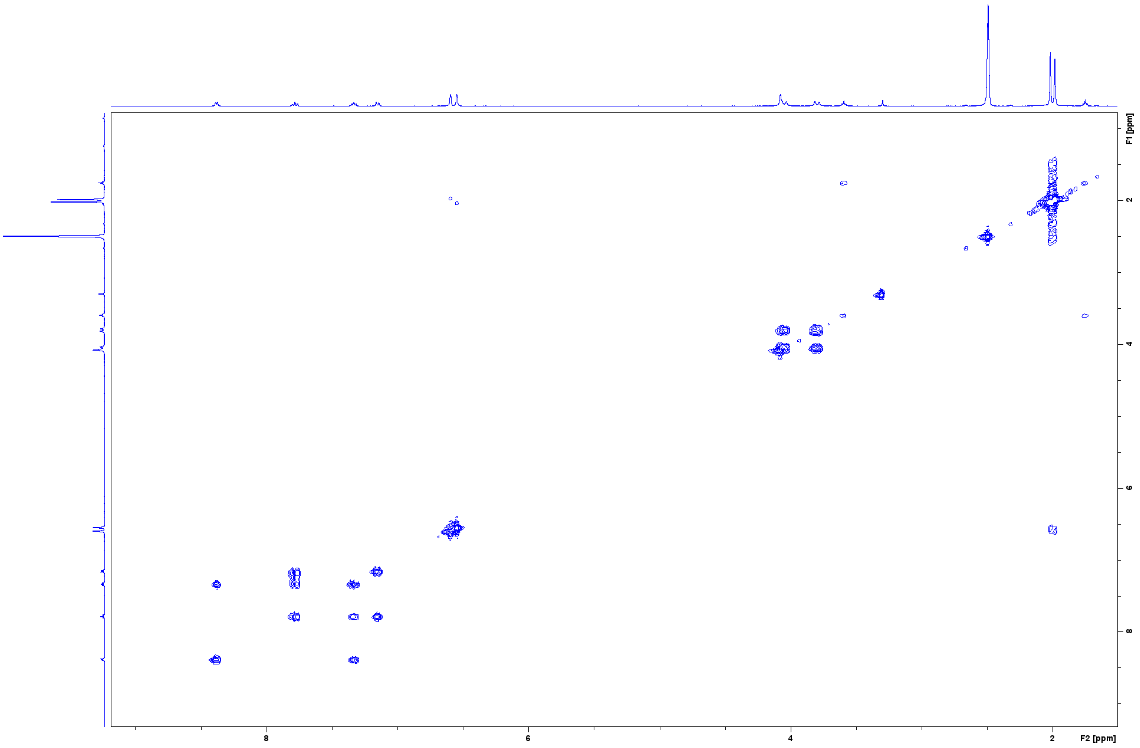


**Figure S20.** COSY NMR spectrum of compound **GaL-Br** in DMSO-d6 at 298K.

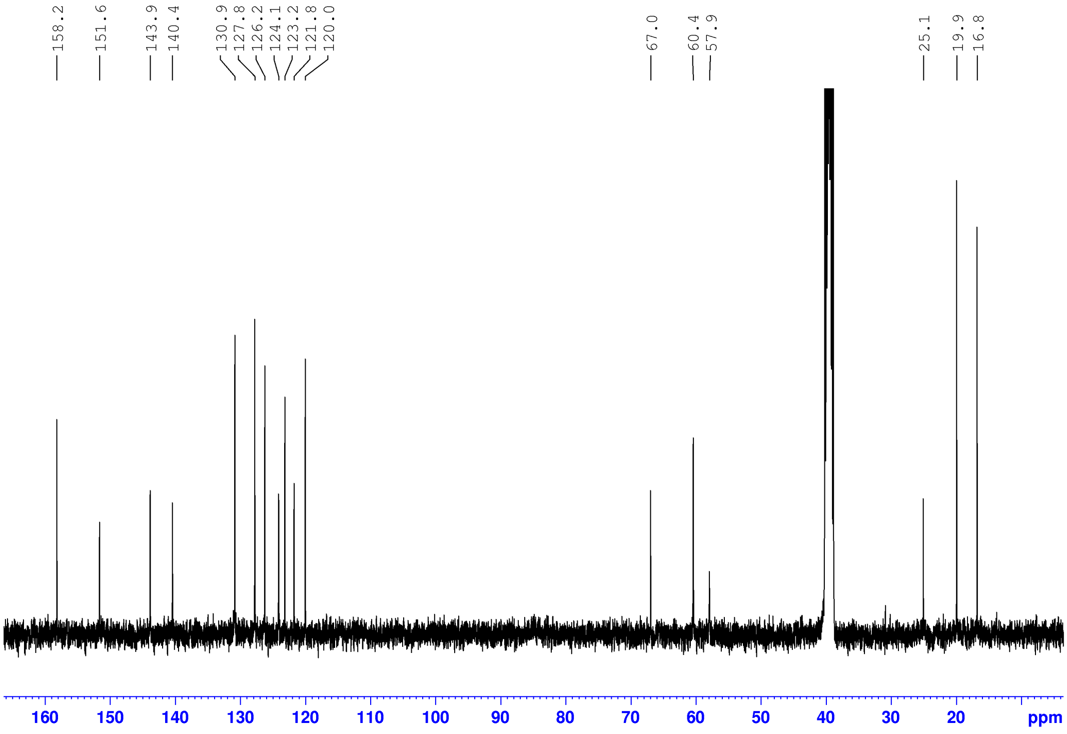


**Figure S21.** $^{13}C\{^{1}H\}$ NMR spectrum of compound **GaL-Br** in DMSO-d6 at 298K.

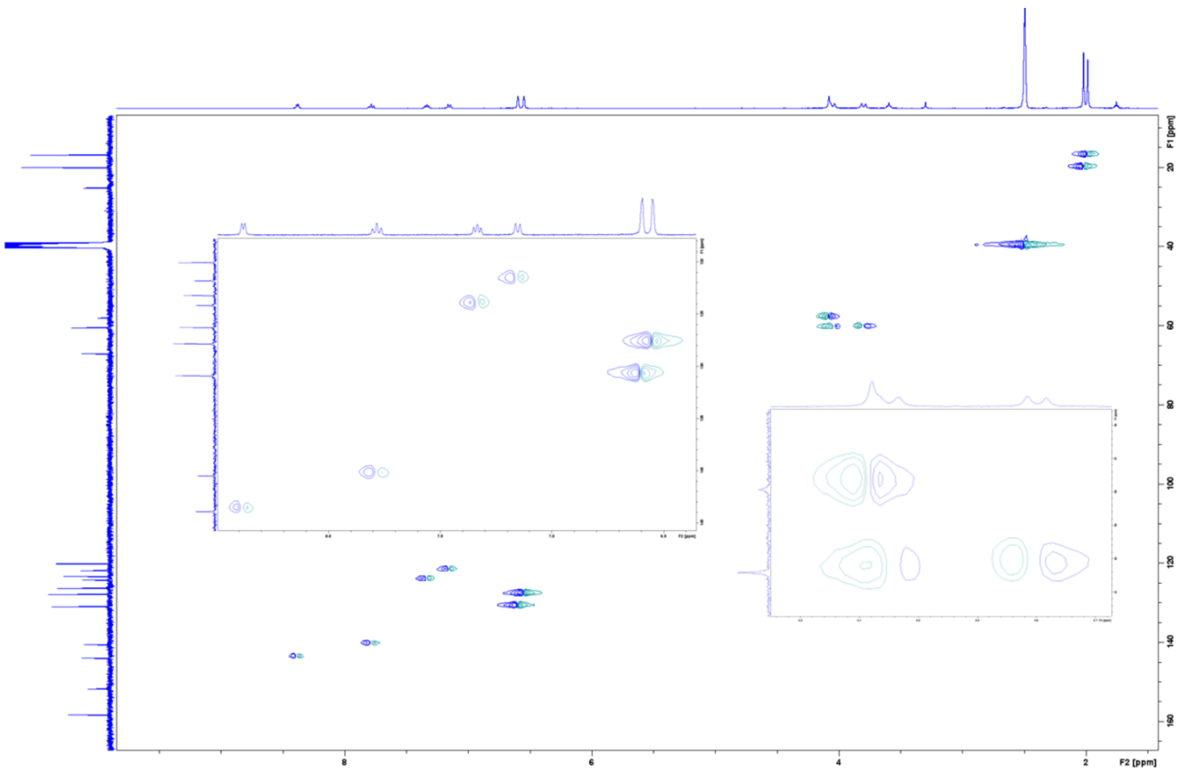


**Figure S22.** $^{1}H$-$^{13}C\{^{1}H\}$ HSQC NMR spectrum of compound **GaL-Br** in DMSO-d$^{6}$ at 298K.

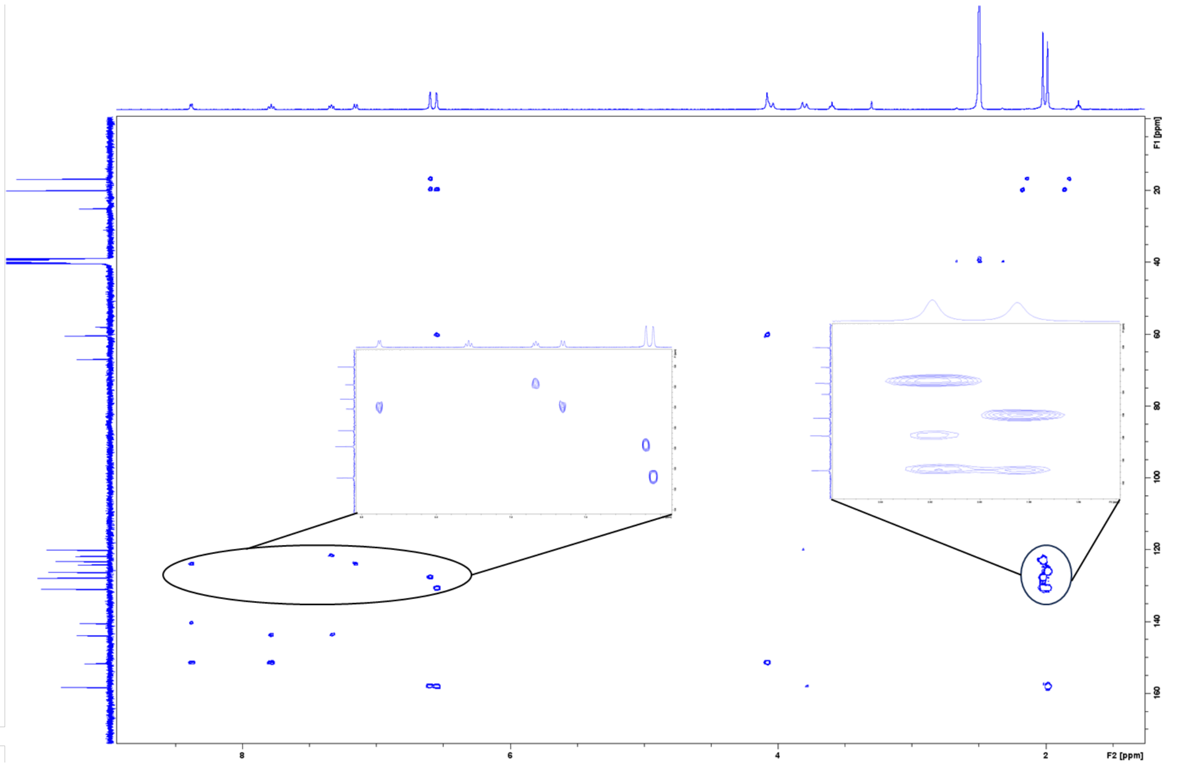


**Figure S23.** $^{1}H$-$^{13}C\{^{1}H\}$ HMBC NMR spectrum of compound **GaL-Br** in DMSO-d$^{6}$ at 298K.

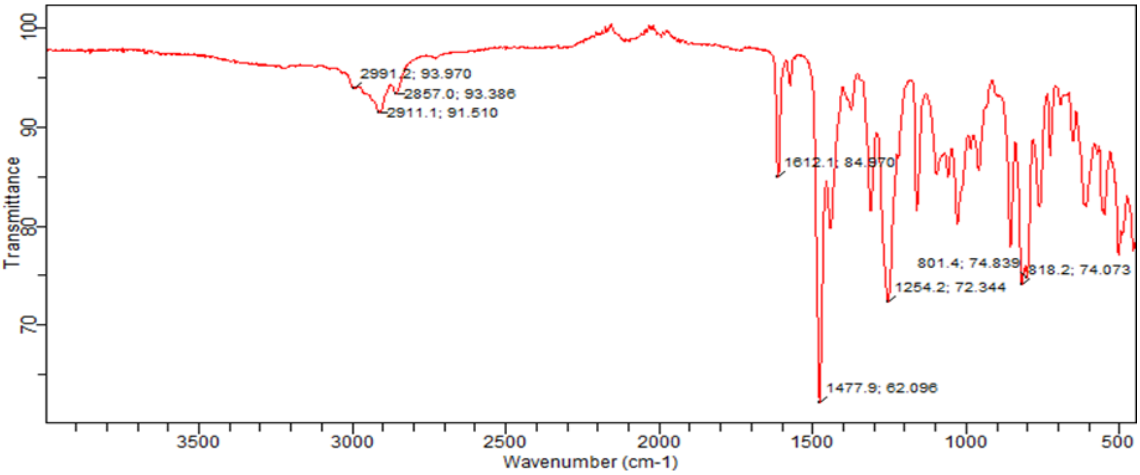


**Figure S24.** IR spectrum of compound **GaL-Br** at 298K.

Spectra for **GaL-I**

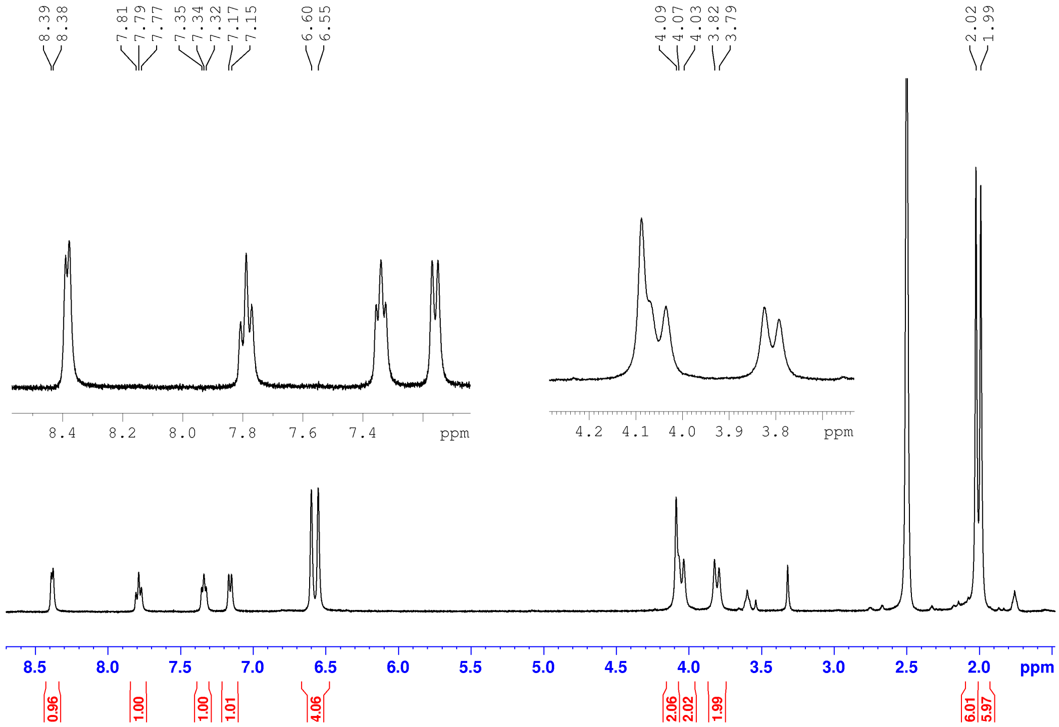


**Figure S25.** $^1$H NMR spectrum of compound **GaL-I** in DMSO-d$^6$ at 298K.

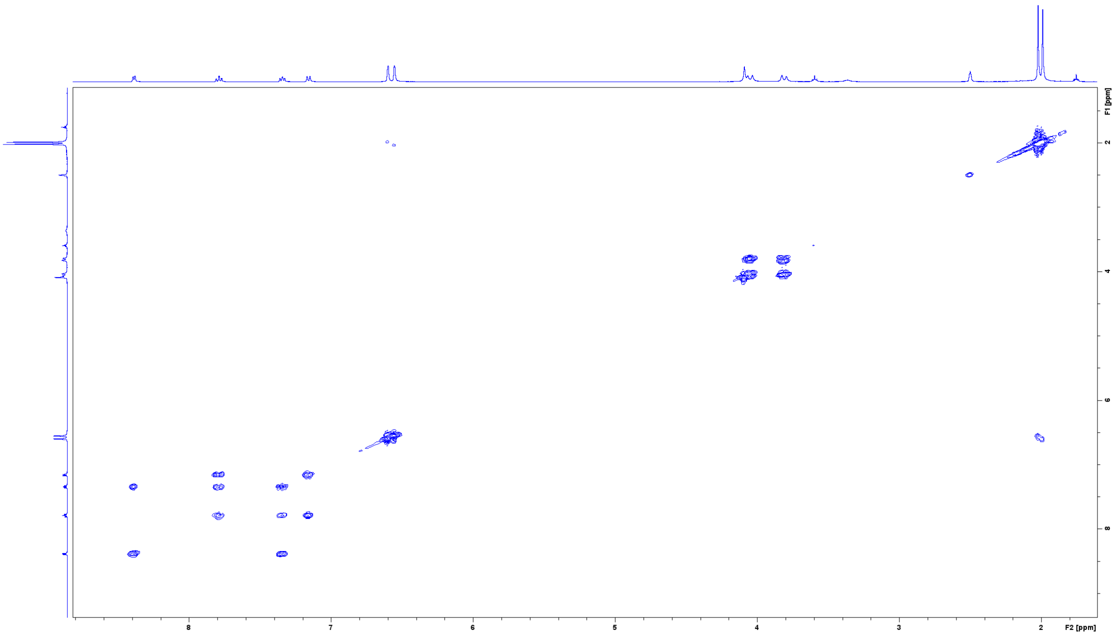


**Figure S26.** COSY NMR spectrum of compound **GaL-I** in DMSO-d$^6$ at 298K.

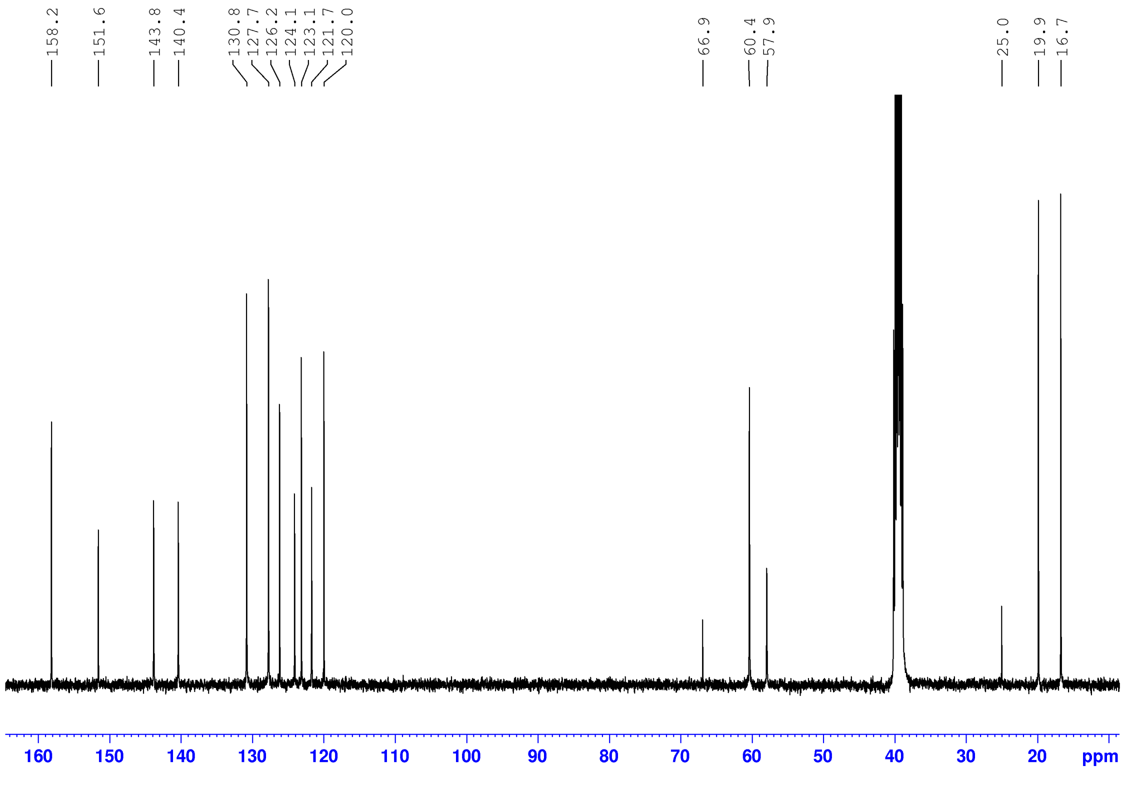


**Figure S27.** $^{13}C\{^1H\}$ NMR spectrum of compound **GaL-I** in DMSO-d$^6$ at 298K.

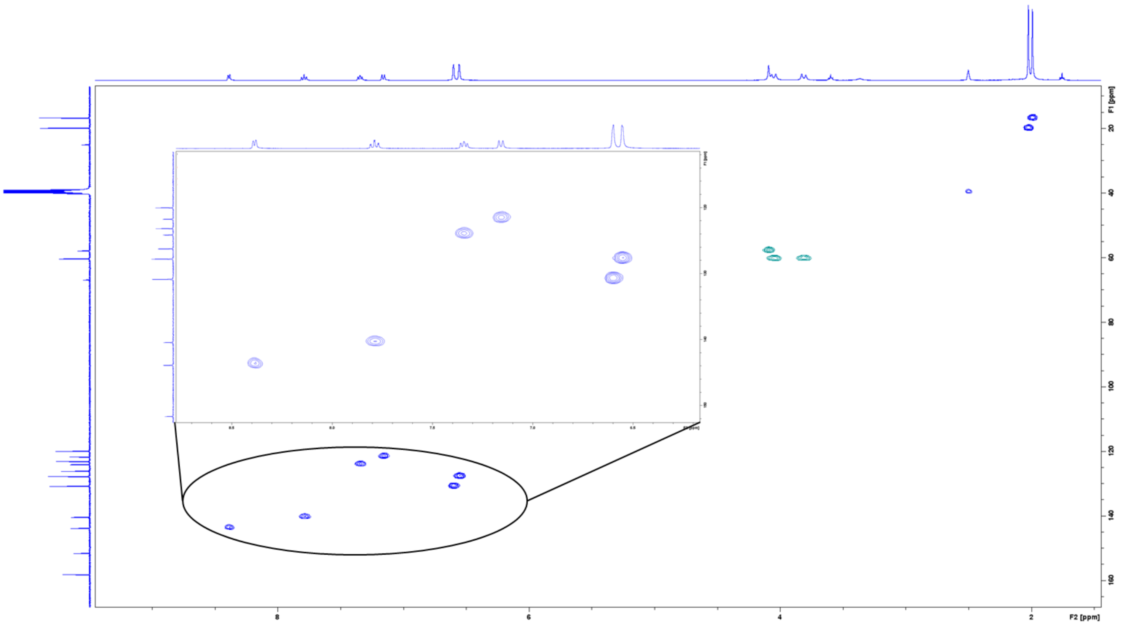

**Figure S28.** $^{1}H$-$^{13}C\{^{1}H\}$ HSQC NMR spectrum of compound **GaL-I** in DMSO-d$^{6}$ at 298K.

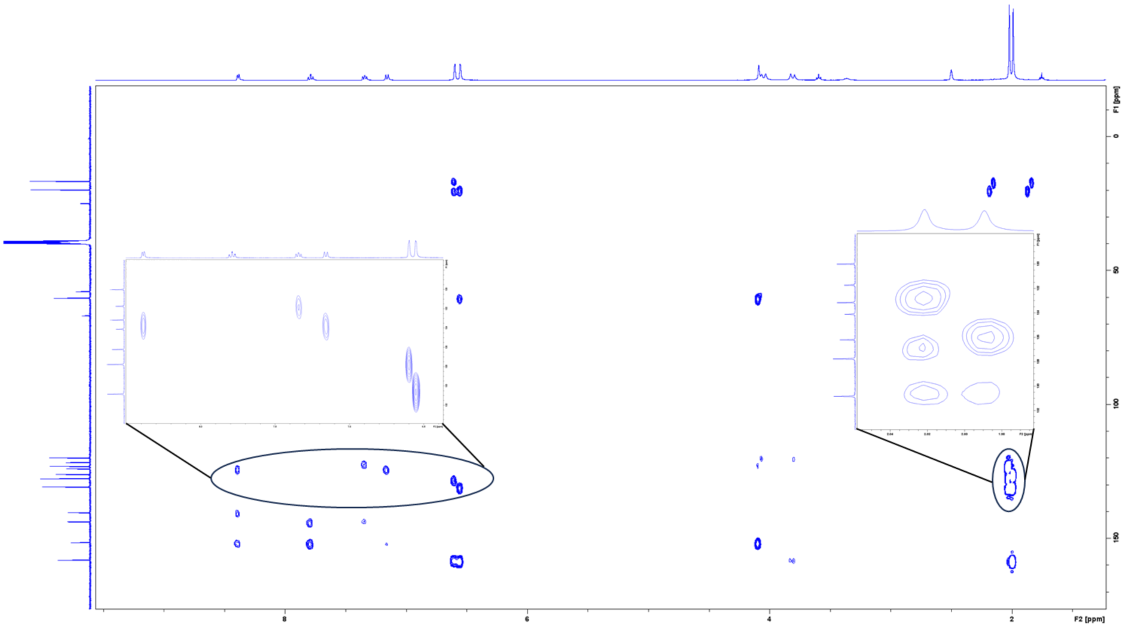

**Figure S29.** $^{1}H$-$^{13}C\{^{1}H\}$ HMBC NMR spectrum of compound **GaL-I** in DMSO-d$^{6}$ at 298K.

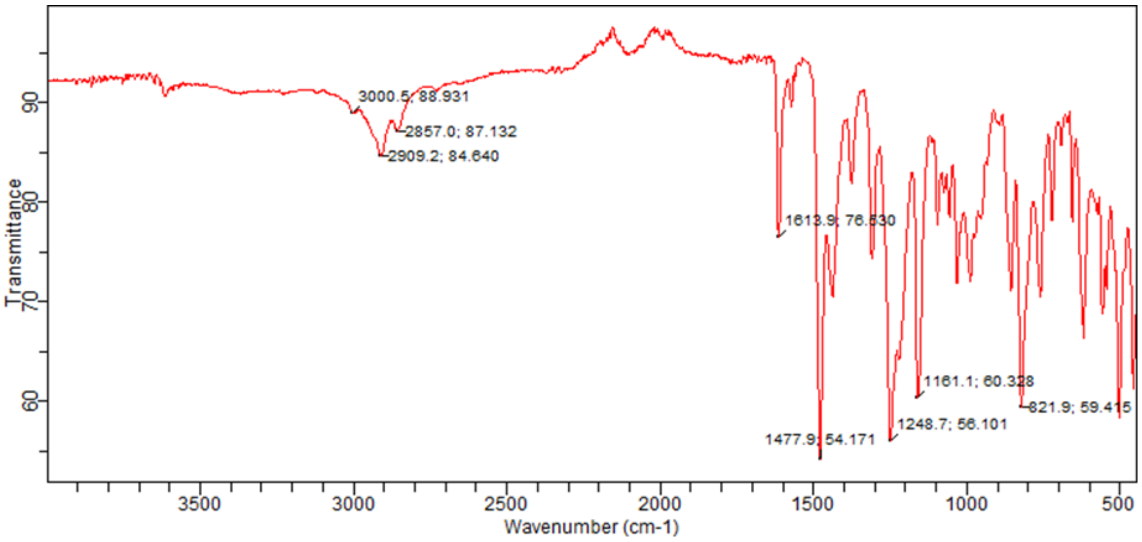


**Figure S30.** IR spectrum of compound **GaL-I** at 298K.

Spectra for **InL-Cl**

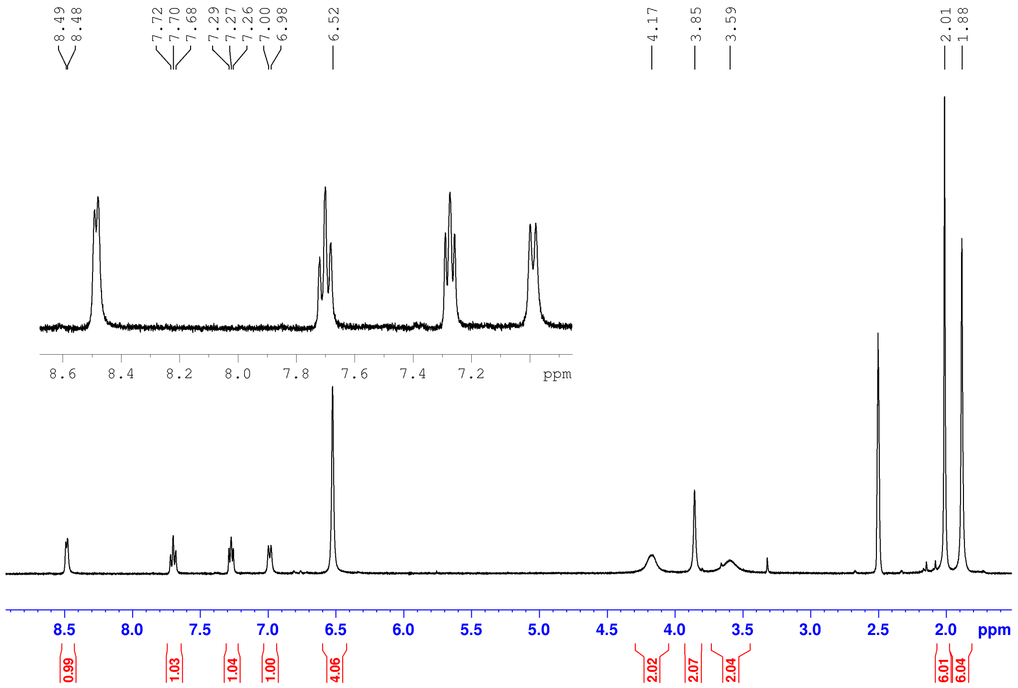


**Figure S31.** $^{1}$H NMR spectrum of compound **InL-Cl** in DMSO-$d^{6}$ at 298K.

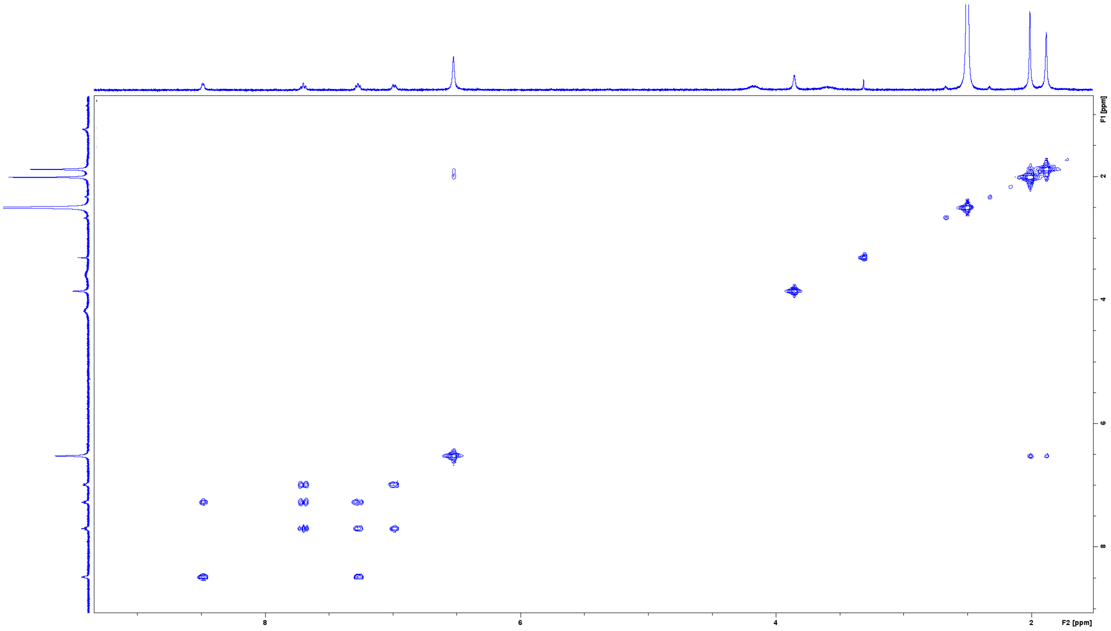


**Figure S32.** COSY NMR spectrum of compound **InL-Cl** in DMSO-d⁶ at 298K.

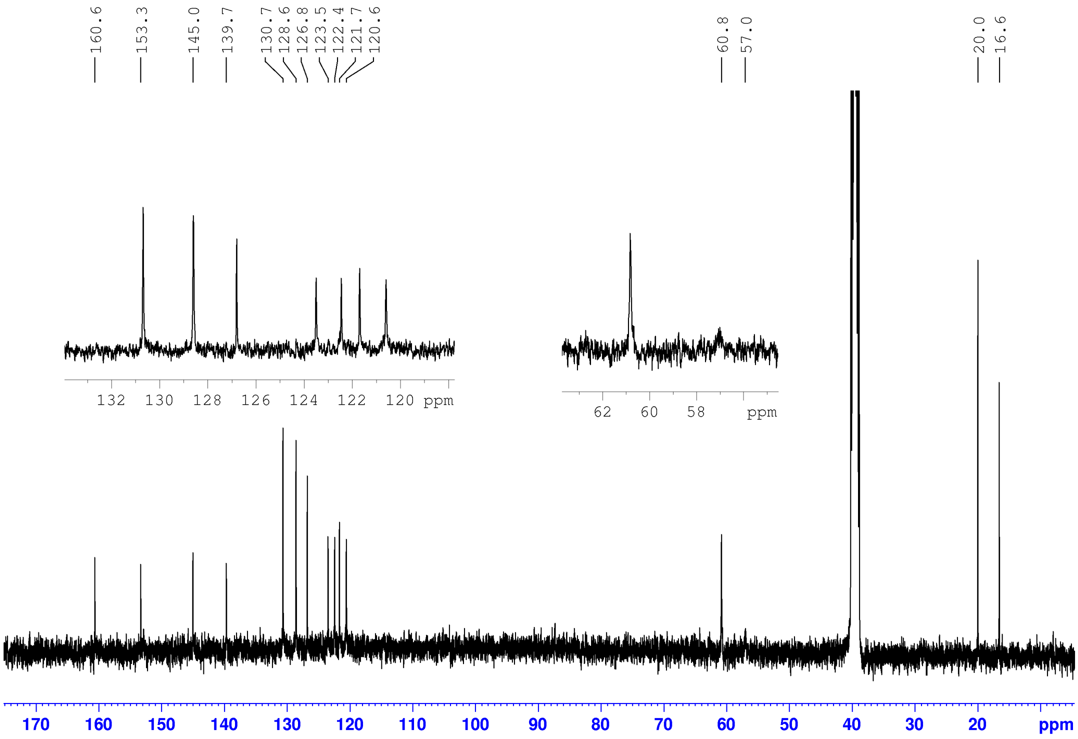


**Figure S33.** $^{13}C\{^{1}H\}$ NMR spectrum of compound **InL-Cl** in DMSO-d⁶ at 298K.

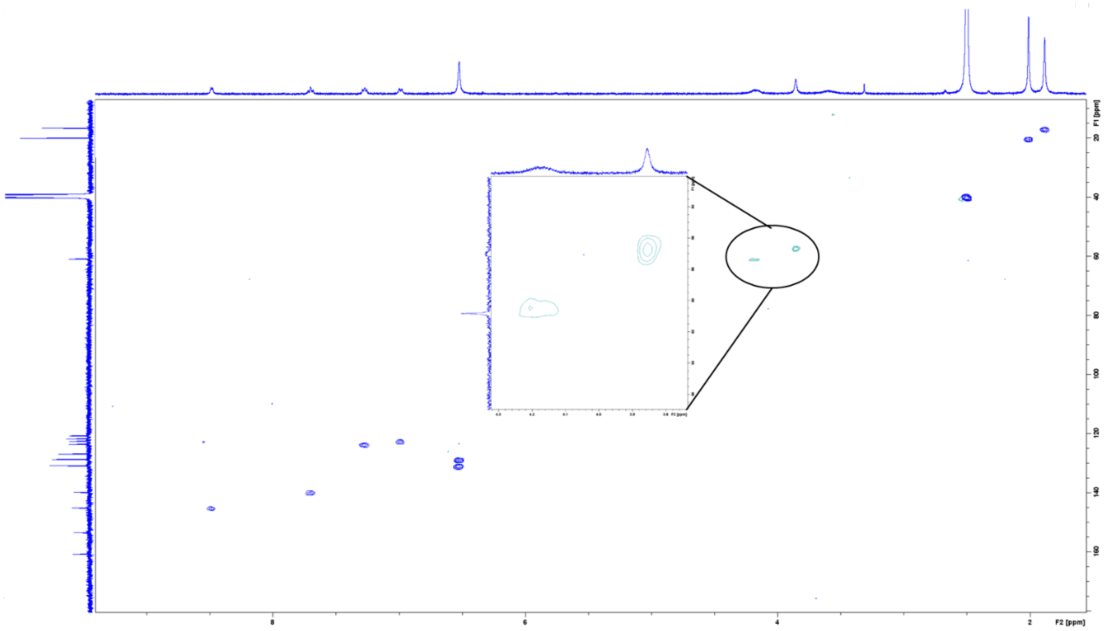


**Figure S34.** $^{1}H$-$^{13}C\{^{1}H\}$ HSQC NMR spectrum of compound **InL-Cl** in DMSO-d$^{6}$ at 298K.

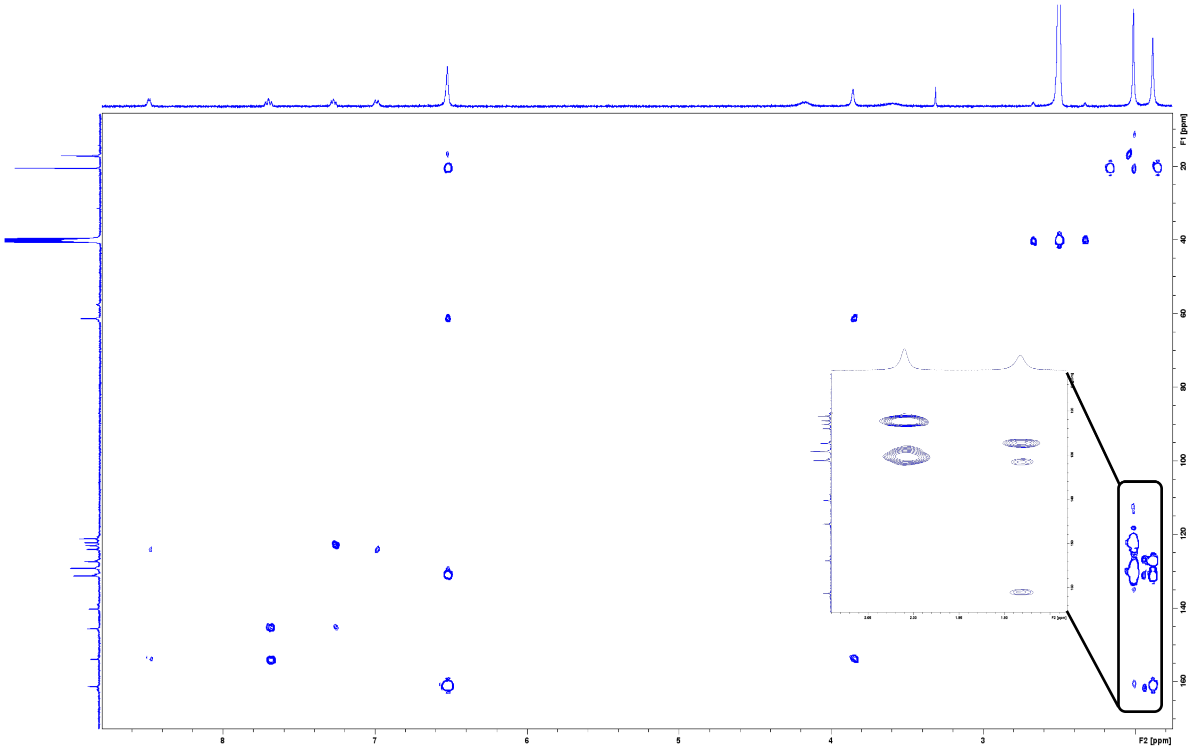


**Figure S35.** $^{1}H$-$^{13}C\{^{1}H\}$ HMBC NMR spectrum of compound **InL-Cl** in DMSO-d$^{6}$ at 298K.

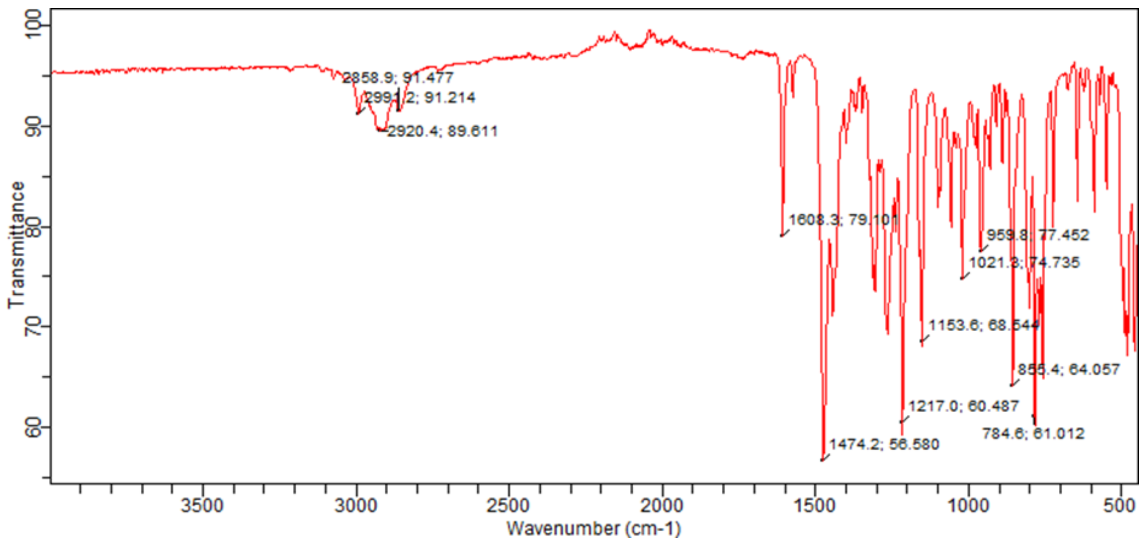


**Figure S36.** IR spectrum of compound **InL-Cl** at 298K.

## [3] Instability of compound **AlL-Br**

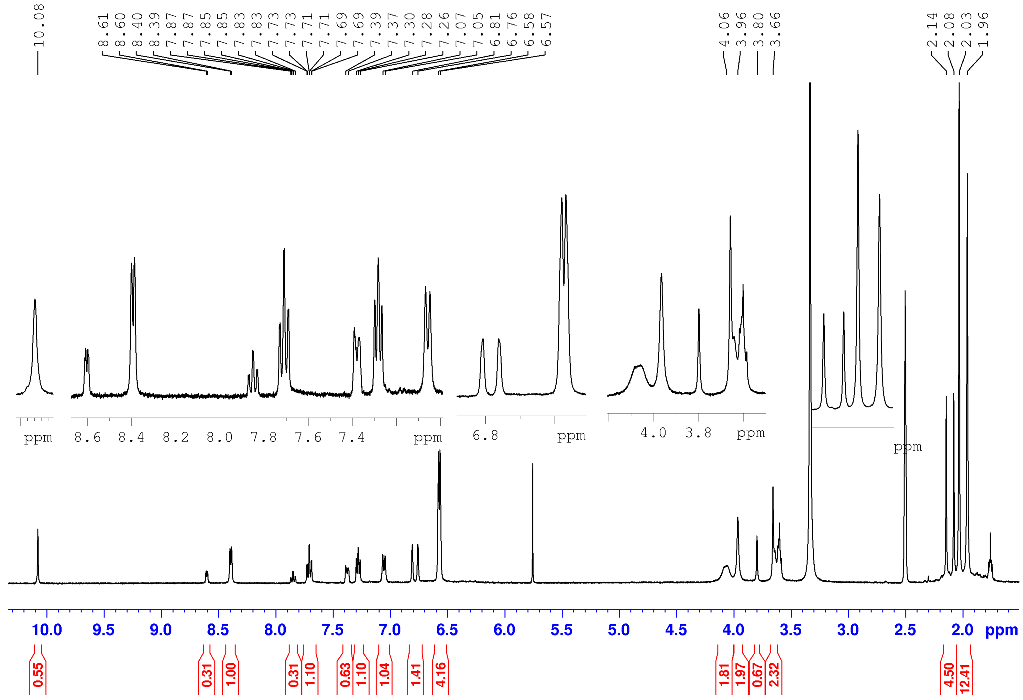


**Figure S37.** $^1$H NMR spectrum of isolated compound **AlL-Br** in “wet” DMSO-d$^6$ at 298K.

## **[4]** X-ray crystallography data for **AlL-Cl**, **GaL-Cl** and **InL-Cl**

Diffraction data were collected using an Oxford Diffraction Supernova diffractometer, equipped with an Atlas CCD area detector and a four-circle kappa goniometer. For the data collection, Mo source with multilayer optics was used. Data integration, scaling, and empirical absorption correction were carried out using the CrysAlis Program package.[2] The structures were solved using direct methods and refined by Full-Matrix-Least-Squares against F2 with SHELX[3] under OLEX2.[4] The non-hydrogen atoms were refined anisotropically, and hydrogen atoms were placed at idealized positions and refined using the riding model. Full-matrix least-squares refinements were carried out by minimizing Σw(*Fo*2 − *Fc*2)2 with the SHELXL weighting scheme and stopped at shift/err < 0.001. The final residual electron density maps showed no remarkable features. Graphics were made with OLEX2 and MERCURY.[5] The structures reported in this paper have been deposited with the Cambridge Crystallographic Data Centre (CCDC) as supplementary publication numbers: 2345917 (**AlL-Cl**), 2338178 (**GaL-Cl**) and 2345889 (**InL-Cl**). It should be noted that the structure of complex **AlL-Cl** is affected by a disorder which could not be satisfactorily modelled. However, despite an Alert A, the refinement parameters are of a high enough quality to demonstrate without any doubt the connectivity of the molecule concerned, which was the purpose of the determination. The disorder is mainly produced by the poor quality of the crystal obtained for the X-ray studies.

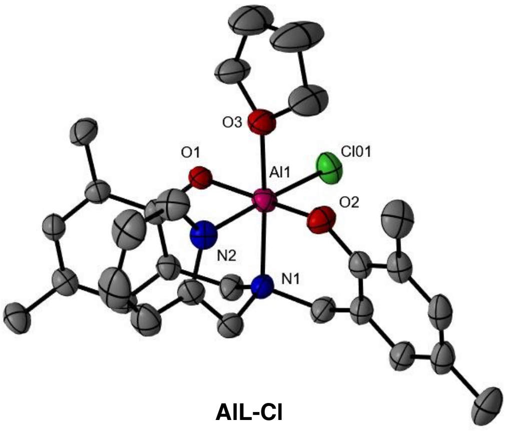


**AlL-Cl**

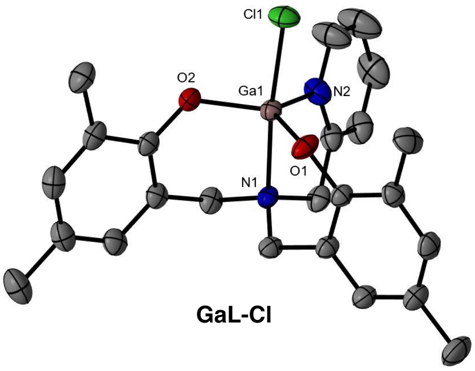


**GaL-Cl**

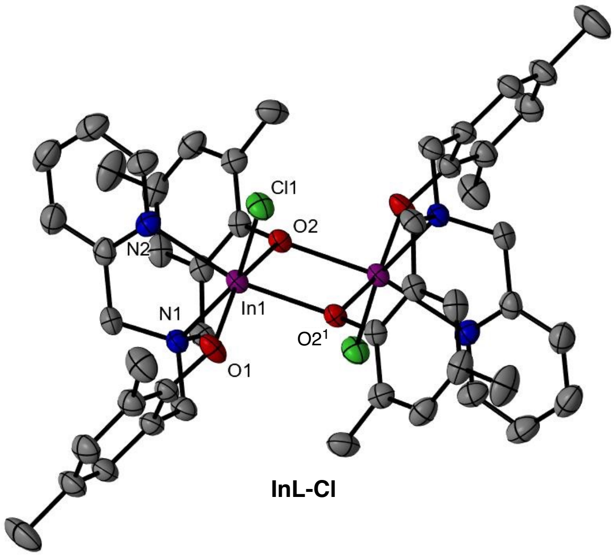


**InL-Cl**

**Table S1.** X-ray crystallographic data for the three complexes.

| | **AlL-Cl** | **GaL-Cl** | **InL-Cl** |
|---|---|---|---|
| **CCDC number** | **2345917** | **2338178** | **2345889** |
| Empirical formula | $C_{60}H_{76}Al_2Cl_2N_4O_7$ | $C_{25}H_{28}Cl_3GaN_2O_2$ | $C_{28}H_{34}ClInN_2O_3$ |
| Formula weight | 1090.10 | 564.56 | 596.84 |
| Temperature/K | 200.00 | 200.0 | 200.00 |
| Crystal system | monoclinic | monoclinic | triclinic |
| Space group | $P2_1/n$ | $P2_1/n$ | P-1 |
| a/Å | 14.3018(8) | 8.5167(4) | 12.1350(5) |
| b/Å | 24.6912(16) | 19.3548(5) | 14.8099(6) |
| c/Å | 17.5672(10) | 15.9323(7) | 15.9232(7) |
| α/° | 90 | 90 | 96.673(2) |
| β/° | 112.081(2) | 102.4920(10) | 99.608(2) |
| γ/° | 90 | 90 | 93.038(2) |
| Volume/$Å^3$ | 5748.5(6) | 2564.09(18) | 2794.8(2) |
| Z | 4 | 4 | 4 |
| $\rho_{calc}g/cm^3$ | 1.260 | 1.462 | 1.418 |
| $\mu/mm^{-1}$ | 0.199 | 1.411 | 0.972 |
| F(000) | 2320.0 | 1160.0 | 1224.0 |
| Crystal size/$mm^3$ | 0.271 × 0.183 × 0.1 | 0.675 × 0.36 × 0.302 | 0.402 × 0.315 × 0.166 |
| Radiation | MoKα (λ = 0.71073) | MoKα (λ = 0.71073) | MoKα (λ = 0.71073) |
| 2Θ range for data collection/° | 4.92 to 56.014 | 4.958 to 59.094 | 4.238 to 55.802 |
| Reflections collected | 100103 | 121654 | 169609 |
| Independent reflections [$R_{int}$] | 13516 [0.4887] | 6248 [0.0885] | 13306 [0.0356] |
| restraints/parameters | 25/684 | 25/302 | 0/549 |
| Goodness-of-fit on $F^2$ | 0.876 | 1.049 | 1.095 |
| Final $R_1$ indexes [I>=2σ (I)] | 0.0664 | 0.0560 | 0.0370 |
| Final R indexes [all data] | $R_1$ = 0.1926, $wR_2$ = 0.1570 | $R_1$ = 0.0686, $wR_2$ = 0.1664 | $R_1$ = 0.0409, $wR_2$ = 0.1313 |
| Largest diff. peak/hole / e $Å^{-3}$ | 0.46/-0.41 | 1.85/-1.32 | 0.62/-0.76 |

## [5] Computational study details

All DFT calculations undertaken using the ORCA 4.2.1 computational software.[6] Solvation optimizations and analytical frequency calculations were performed at the RI-B97-D3/def2-TZVP level of theory.[7-9] Final single-point energies and solvation corrections were calculated at RIJCOSX-ωB97M-V/def2-TZVPP level of theory.[9,10] All solvation corrections were calculated using the SMD model with a parameters for hexan-1-ol,[11] which has previously been shown to be a good solvation approximation for an epoxide solvent environment.[12] Analytical frequencies were calculated for inclusion of the Zero Point Energy (ZPE) correction and entropic contributions to the free energy term as well as confirming all intermediate were true with no imaginary modes and all transition states had the correct critical frequency of decomposition. Numerical precision integration grids were increased beyond the default settings, to Grid4 for

the SCF step and Grid5 for the final energy evaluation. Concentration correction, to account for the low catalyst loading and substrate/solvent environment was applied as a free energy correction based on the Van't Hoff reaction quotient equation RT ln(Q)[13] where Q accounts for the concentration gradient between the substrate and the catalyst. Graphical visualization and structural analysis performed from the DFT calculations using Avogadro 1.2.0.[14]

## [6] Computational study data

Calculated coordinates for all presented structures:

**$CO_2$**

| | | | |
|---|---|---|---|
| O | -0.0008559638 | -0.0299545876 | 1.1529361660 |
| C | -0.0000070060 | 0.0988880593 | -0.0112345489 |
| O | 0.0008629698 | 0.2310665283 | -1.1750344554 |

**Styrene oxide (SO)**

| | | | |
|---|---|---|---|
| C | -3.0800335132 | 4.4985799006 | 0.1344135829 |
| C | -4.3970004542 | 4.2266888437 | 0.5077311009 |
| C | -4.6570623546 | 3.4557573008 | 1.6427860534 |
| C | -3.5929527738 | 2.9556393634 | 2.3986450770 |
| C | -2.2768895775 | 3.2309700338 | 2.0288732952 |
| C | -2.0099088588 | 4.0115515334 | 0.8960522774 |
| C | -0.6150326308 | 4.3416684204 | 0.5012892399 |
| H | -2.8772612244 | 5.0941976364 | -0.7591143321 |
| H | -5.2223188664 | 4.6118942972 | -0.0934037811 |
| H | -5.6859518959 | 3.2371805747 | 1.9328204848 |
| H | -3.7904288208 | 2.3416335265 | 3.2790929981 |
| H | -1.4404439971 | 2.8246036745 | 2.6001592083 |
| C | 0.3639867618 | 4.8880593022 | 1.4639801628 |
| H | 1.1683878938 | 5.5417815202 | 1.1094763306 |
| O | 0.4438459971 | 3.5229399100 | 1.0348372250 |
| H | 0.0451981469 | 5.0339270574 | 2.5018765650 |
| H | -0.4805114127 | 4.6116504958 | -0.5534701393 |

**Cyclic carbonate product**

| | | | |
|---|---|---|---|
| C | 3.4681770742 | -0.8784614066 | 12.9102203234 |
| C | 3.5222906989 | 0.3833194946 | 12.3047762623 |
| C | 3.0785744404 | 0.5307779186 | 10.9862748736 |
| H | 3.0914371890 | 1.5136445371 | 10.5137805042 |
| C | 2.5973144021 | -0.5743075279 | 10.2815127703 |
| H | 2.2492151842 | -0.4485157230 | 9.2551971851 |
| C | 2.5552770427 | -1.8322793749 | 10.8853664061 |
| H | 2.1757536575 | -2.6929896261 | 10.3328714577 |
| C | 4.1136631529 | 1.5376414484 | 13.0779480595 |
| C | 5.6453881259 | 1.6808449602 | 12.9193318371 |
| H | 6.1347742696 | 1.9857279450 | 13.8569391449 |
| H | 6.1175425666 | 0.7731569093 | 12.5251840828 |
| O | 3.6379913884 | 2.8099481193 | 12.5926289904 |

| | | | |
|---|---|---|---|
| H | 3.8315821570 | 1.4635739207 | 14.1378890391 |
| C | 2.9905891305 | -1.9825467095 | 12.2041298260 |
| H | 2.9488651589 | -2.9596922324 | 12.6877198000 |
| H | 3.7966130979 | -0.9973210927 | 13.9462160757 |
| O | 4.4602373904 | 4.4252035927 | 11.2172448217 |
| C | 4.6165670058 | 3.4253658186 | 11.8577990843 |
| O | 5.7923557559 | 2.7347055402 | 11.9549579922 |

**TBAI**

| | | | |
|---|---|---|---|
| N | -1.1329029152 | 0.7185947524 | -0.1043242328 |
| C | 0.3901083536 | 0.7445013625 | -0.1462321721 |
| C | -1.6436783160 | -0.5912479971 | 0.4987707990 |
| C | -1.6060047535 | 0.8757591526 | -1.5397312823 |
| C | -1.6708714049 | 1.8184818932 | 0.8002291196 |
| H | -1.3637942311 | 1.5368240427 | 1.8379493303 |
| H | -2.7720987958 | 1.7094661996 | 0.7829600012 |
| C | -1.2590382449 | 3.2382925560 | 0.4500003671 |
| H | -0.1537323584 | 3.3437219764 | 0.4856991344 |
| C | -1.8762869338 | 4.2223180037 | 1.4554752875 |
| H | -1.5724250524 | 3.5207454027 | -0.5803046180 |
| H | -1.5870983968 | 3.9101645151 | 2.4820437933 |
| H | -2.9843421600 | 4.1339821107 | 1.4177246332 |
| C | -1.4607525815 | 5.6709778653 | 1.2048607444 |
| H | -0.3603086843 | 5.7937258171 | 1.2809225591 |
| H | -1.9229967881 | 6.3561271208 | 1.9426060274 |
| H | -1.7640485274 | 6.0139368951 | 0.1932456458 |
| H | 0.6827878662 | -0.1262911410 | -0.7637324395 |
| C | 1.0816383925 | 0.7159636122 | 1.2065830162 |
| H | 0.6622032160 | 1.6498894813 | -0.7239389490 |
| H | 0.5412981540 | 0.0634101197 | 1.9319413383 |
| H | 1.0413794677 | 1.7212373172 | 1.6758100916 |
| C | 2.5403598176 | 0.2605624989 | 1.0933414641 |
| H | 2.5637883574 | -0.7729952518 | 0.6788872251 |
| C | 3.2520625609 | 0.2902796926 | 2.4464545935 |
| H | 3.0887711836 | 0.8927708216 | 0.3575516094 |
| H | 2.7043177707 | -0.3200427513 | 3.1928558147 |
| H | 3.3010163427 | 1.3228888718 | 2.8488865337 |
| H | 4.2889152728 | -0.0935309476 | 2.3704418280 |
| C | -1.0054543419 | -1.8778888540 | 0.0054503954 |
| H | -2.7349608375 | -0.5950690674 | 0.3174287568 |
| H | -1.5104680614 | -0.4985963829 | 1.6097540894 |
| H | -1.0712359615 | -1.9942505422 | -1.1008958470 |
| C | -1.6846226178 | -3.0732267190 | 0.6926359147 |
| H | 0.0729907124 | -1.8984148212 | 0.2699390668 |
| H | -2.7646817012 | -3.0844428208 | 0.4264321674 |
| H | -1.6422491851 | -2.9111045810 | 1.7918357185 |
| C | -1.0435261043 | -4.4109121493 | 0.3276036264 |
| H | -1.0863952329 | -4.6025180832 | -0.7655461638 |
| H | -1.5534364096 | -5.2533381731 | 0.8353702105 |
| H | 0.0241537393 | -4.4394558600 | 0.6289850236 |
| H | -1.2392695183 | -0.0207988910 | -2.0772999660 |

| | | | |
|---|---|---|---|
| H | -1.0626304401 | 1.7460573807 | -1.9562184722 |
| C | -3.1038279446 | 1.0483033581 | -1.7538195502 |
| H | -3.6690871886 | 0.2220828771 | -1.2739117406 |
| C | -3.4437390050 | 1.0820419216 | -3.2505347281 |
| H | -3.4572746459 | 1.9889476936 | -1.2817840060 |
| H | -3.0963709870 | 0.1386520051 | -3.7266027383 |
| H | -2.8668511597 | 1.8975528390 | -3.7403489689 |
| C | -4.9370684681 | 1.2752791625 | -3.5137345509 |
| H | -5.5350699773 | 0.4563857364 | -3.0633327481 |
| H | -5.1575013568 | 1.2931691314 | -4.5992134835 |
| H | -5.3034359019 | 2.2292353881 | -3.0814114441 |
| I | -1.1957512453 | -0.0281484196 | 4.1126488135 |

**TBACl**

| | | | |
|---|---|---|---|
| N | -1.1303474355 | 0.7199028871 | -0.0774049486 |
| C | 0.3909654077 | 0.7487268343 | -0.1218404796 |
| C | -1.6365873508 | -0.5882623852 | 0.5302590279 |
| C | -1.5996328375 | 0.8764640288 | -1.5105972333 |
| C | -1.6686255772 | 1.8189466115 | 0.8253334661 |
| H | -1.3496865714 | 1.5414286713 | 1.8517650986 |
| H | -2.7609514791 | 1.7056909365 | 0.8104243159 |
| C | -1.2684792768 | 3.2348319952 | 0.4550856653 |
| H | -0.1721232352 | 3.3440443280 | 0.4676683031 |
| C | -1.8690673122 | 4.2174590502 | 1.4678255289 |
| H | -1.6061294757 | 3.5098449374 | -0.5596454976 |
| H | -1.5562346475 | 3.9175039167 | 2.4805133228 |
| H | -2.9682327356 | 4.1281990403 | 1.4509087753 |
| C | -1.4622304599 | 5.6632120649 | 1.1952055764 |
| H | -0.3695572535 | 5.7838919640 | 1.2466308275 |
| H | -1.9069530451 | 6.3471753239 | 1.9311235884 |
| H | -1.7868968145 | 5.9904745104 | 0.1953263644 |
| H | 0.6806075677 | -0.1324846775 | -0.7095943529 |
| C | 1.0882050749 | 0.7572485203 | 1.2272013998 |
| H | 0.6574466976 | 1.6333860668 | -0.7171398428 |
| H | 0.5342483861 | 0.1660868639 | 1.9815964755 |
| H | 1.0955042204 | 1.7791675192 | 1.6384646373 |
| C | 2.5253634019 | 0.2459074663 | 1.1087083696 |
| H | 2.5058160810 | -0.7958424335 | 0.7425923942 |
| C | 3.2540879883 | 0.3037998169 | 2.4502652246 |
| H | 3.0832277752 | 0.8260598918 | 0.3513801402 |
| H | 2.6971861503 | -0.2531256525 | 3.2172279667 |
| H | 3.3459258038 | 1.3416574826 | 2.8035333780 |
| H | 4.2661586518 | -0.1186198883 | 2.3768083087 |
| C | -1.0218757878 | -1.8702322573 | 0.0029895175 |
| H | -2.7222454254 | -0.5813255573 | 0.3684433946 |
| H | -1.4724043142 | -0.4980621295 | 1.6310105453 |
| H | -1.1034614927 | -1.9693816189 | -1.0946124289 |
| C | -1.7151880768 | -3.0632454090 | 0.6745302902 |
| H | 0.0493071217 | -1.9140913897 | 0.2553428576 |
| H | -2.7858426502 | -3.0595972290 | 0.4087466648 |
| H | -1.6667864512 | -2.9223231040 | 1.7662230411 |

| | | | |
|---|---|---|---|
| C | -1.0883470959 | -4.3991760314 | 0.2844166572 |
| H | -1.1370434990 | -4.5653089862 | -0.8031213767 |
| H | -1.6025380789 | -5.2377483785 | 0.7740673095 |
| H | -0.0295536833 | -4.4398946928 | 0.5812851353 |
| H | -1.2056523854 | 0.0059388071 | -2.0532069418 |
| H | -1.0909224864 | 1.7649879135 | -1.9085258160 |
| C | -3.0988060200 | 1.0028229104 | -1.7300005046 |
| H | -3.6347481354 | 0.1441001742 | -1.2980144283 |
| C | -3.4147234441 | 1.0845479884 | -3.2276295158 |
| H | -3.4868333697 | 1.9047196294 | -1.2318535257 |
| H | -3.0288506566 | 0.1823679914 | -3.7312741940 |
| H | -2.8730942088 | 1.9378638416 | -3.6697296165 |
| C | -4.9105358020 | 1.2271125756 | -3.5007094099 |
| H | -5.4711037820 | 0.3711534685 | -3.0964990192 |
| H | -5.1156013814 | 1.2827110341 | -4.5785448541 |
| H | -5.3144803113 | 2.1385524432 | -3.0353576842 |
| Cl | -1.1311735108 | 0.0707884360 | 3.7128047407 |

**TBA$^+$**

| | | | |
|---|---|---|---|
| N | -1.0582275628 | 0.6943340620 | 0.0044740464 |
| C | 0.4655863315 | 0.6976393521 | -0.0343121956 |
| C | -1.5868550448 | -0.5967326115 | 0.6183637048 |
| C | -1.5264194090 | 0.8435597987 | -1.4379911565 |
| C | -1.5846894309 | 1.8317841602 | 0.8713039448 |
| H | -1.2337155695 | 1.6179860471 | 1.8888094632 |
| H | -2.6765417083 | 1.7221261661 | 0.8763682569 |
| C | -1.1923084526 | 3.2395799548 | 0.4567247943 |
| H | -0.0974510774 | 3.3580412396 | 0.4469329262 |
| C | -1.7918427858 | 4.2613223700 | 1.4332277985 |
| H | -1.5518271032 | 3.4660199771 | -0.5591245657 |
| H | -1.4396257172 | 4.0382799390 | 2.4539662922 |
| H | -2.8881512500 | 4.1453247550 | 1.4529868639 |
| C | -1.4298165370 | 5.6977098174 | 1.0633074277 |
| H | -0.3404810770 | 5.8490853247 | 1.0690569978 |
| H | -1.8685014783 | 6.4087405484 | 1.7752469745 |
| H | -1.8004203867 | 5.9576677912 | 0.0610155711 |
| H | 0.7484470999 | -0.1263612082 | -0.7014734041 |
| C | 1.1805153952 | 0.5726080728 | 1.3000423665 |
| H | 0.7479759363 | 1.6313381394 | -0.5370713283 |
| H | 0.9066455123 | -0.3647065175 | 1.8091517485 |
| H | 0.9063203473 | 1.3989033238 | 1.9743109797 |
| C | 2.7006997692 | 0.5926609046 | 1.0866400980 |
| H | 2.9831202786 | -0.2281154745 | 0.4067989582 |
| C | 3.4708675454 | 0.4660277136 | 2.3988361167 |
| H | 2.9838292513 | 1.5278313698 | 0.5757409985 |
| H | 3.2312992320 | -0.4761331540 | 2.9132031080 |
| H | 3.2326103859 | 1.2930302543 | 3.0836071275 |
| H | 4.5539705292 | 0.4823249749 | 2.2214170292 |
| C | -1.2143772831 | -1.8872081728 | -0.0916009135 |
| H | -2.6773204870 | -0.4812583066 | 0.6602670019 |
| H | -1.2227209663 | -0.6055997063 | 1.6534132160 |

| | | | |
|---|---|---|---|
| H | -1.5874061350 | -1.8881685308 | -1.1278175444 |
| C | -1.8133016758 | -3.0903374965 | 0.6501955696 |
| H | -0.1211239516 | -2.0096427341 | -0.1415941147 |
| H | -2.9078142689 | -2.9719191722 | 0.7110495135 |
| H | -1.4451998735 | -3.0970024851 | 1.6894366382 |
| C | -1.4732572425 | -4.4151163514 | -0.0285557038 |
| H | -1.8597985069 | -4.4469358713 | -1.0577143672 |
| H | -1.9112346742 | -5.2602861041 | 0.5179682745 |
| H | -0.3858491247 | -4.5737875203 | -0.0727059370 |
| H | -1.0590908374 | 0.0175146999 | -1.9889397651 |
| H | -1.0736116711 | 1.7736433460 | -1.8041911033 |
| C | -3.0282103252 | 0.8551247841 | -1.6662761178 |
| H | -3.4897641288 | -0.0753664676 | -1.3004444872 |
| C | -3.3323039358 | 1.0036978416 | -3.1636699287 |
| H | -3.5041308300 | 1.6868812620 | -1.1236763751 |
| H | -2.8548458629 | 0.1769989723 | -3.7152689094 |
| H | -2.8701148714 | 1.9320167514 | -3.5382961779 |
| C | -4.8317446684 | 1.0198352885 | -3.4510619949 |
| H | -5.3125355150 | 0.0889926900 | -3.1165345072 |
| H | -5.0240849483 | 1.1262328779 | -4.5264863394 |
| H | -5.3277160748 | 1.8574424765 | -2.9391556379 |

**GaL – catalyst**

| | | | |
|---|---|---|---|
| Ga | 2.50008377673507 | 7.04538441454395 | 16.23184356283787 |
| I | 1.58311394949976 | 7.98104578093827 | 13.94470538870838 |
| O | 4.23482854542096 | 7.69140963693093 | 15.95225142709256 |
| N | 1.12025829279590 | 7.92241168094720 | 17.56209540024843 |
| C | 2.62656421748986 | 7.25094588933692 | 19.34923736946741 |
| N | 3.30526022539254 | 6.38989785340991 | 18.37768135190355 |
| C | 5.20685583861081 | 7.95697310213023 | 18.13026921098308 |
| C | 5.29198228994374 | 9.76369233665238 | 16.49188663240625 |
| C | 1.26443609168362 | 7.67349171702546 | 18.88144314067428 |
| C | 5.99881174969579 | 10.52153405064779 | 17.42763071188606 |
| C | 5.91038412181968 | 8.75368493772199 | 19.04245420104438 |
| C | 4.88578987187066 | 8.45734945974108 | 16.85426031237625 |
| C | 4.78445212988814 | 6.55942030593931 | 18.44647649409057 |
| C | 6.32061231983453 | 10.04641597781910 | 18.70956141983881 |
| C | 2.96635190876563 | 4.95717151312291 | 18.59772763551472 |
| C | 0.68251011200116 | 4.23150971201666 | 19.43557530450183 |
| C | 1.51254004931290 | 4.67296369608522 | 18.39947865099877 |
| C | -1.00506300163120 | 8.36286640304353 | 19.28988595032127 |
| C | -0.05294930616651 | 8.39204093019578 | 17.09307198572834 |
| C | 0.99173644581222 | 4.86970520965754 | 17.10497167577939 |
| C | 0.20778982347156 | 7.88023277573927 | 19.76883670371773 |
| C | -0.67307020391853 | 3.97731268840440 | 19.21745789037190 |
| C | 4.96370605592749 | 10.28521675501981 | 15.12375201112134 |
| C | -1.13443916142629 | 8.63598725711554 | 17.92741217843333 |
| C | 7.08867155444537 | 10.90387897300804 | 19.68099424437817 |
| O | 1.78918328616668 | 5.30283925581780 | 16.10607117581462 |
| C | -0.37897546850043 | 4.61723253722702 | 16.85854070674488 |
| C | -1.17347730261064 | 4.17858603776185 | 17.92006441042139 |

| | | | |
|---|---|---|---|
| C | -0.93196433810435 | 4.83576789755010 | 15.48093078511481 |
| C | -1.56858218037433 | 3.48200831994134 | 20.32278699594258 |
| H | -1.08023391469358 | 3.57249076097644 | 21.30295792456497 |
| H | -1.83279735096411 | 2.42160457354331 | 20.18266538059156 |
| H | -2.51355099164420 | 4.04483044250157 | 20.36100185273162 |
| H | 8.09371795811372 | 11.14304956836957 | 19.29960412678619 |
| H | 7.21146035674467 | 10.39765042807933 | 20.64824185297701 |
| H | 6.57914747290704 | 11.86244694946122 | 19.86488732159549 |
| H | 5.30342776951831 | 11.32246737398538 | 15.00477655237801 |
| H | 3.88270668077050 | 10.23503108136501 | 14.92466630565933 |
| H | 5.43533407008180 | 9.66720792067662 | 14.34460995604334 |
| H | 6.15197665004118 | 8.34541309485837 | 20.02904428590618 |
| H | 6.30984966510735 | 11.53218242790125 | 17.14520413807585 |
| H | 1.10921440108511 | 4.08323203834178 | 20.43282330594440 |
| H | -0.72431311671020 | 5.85587465952713 | 15.12260091792725 |
| H | -2.01528415362091 | 4.65872714259117 | 15.45643537470581 |
| H | -0.44880986295013 | 4.16711193336825 | 14.75235487133353 |
| H | -2.23465195441939 | 3.99028819618173 | 17.72872246663694 |
| H | 5.20122804357221 | 5.85994669694302 | 17.70517765071466 |
| H | 5.14735528334943 | 6.25221796744832 | 19.44583505035433 |
| H | 3.23901595332920 | 8.16299874326538 | 19.46220130371028 |
| H | 2.55206246270649 | 6.77184251060321 | 20.34237385901238 |
| H | 3.29058211447333 | 4.65996620024763 | 19.61357918805815 |
| H | 3.56172978006864 | 4.38865819610074 | 17.86686860819061 |
| H | 0.34551488205619 | 7.64152398818716 | 20.82340031623001 |
| H | -1.84363574084435 | 8.51685175702698 | 19.97035960659992 |
| H | -2.06424522973645 | 9.01512849136080 | 17.50565408220810 |
| H | -0.09179792739391 | 8.54961779459642 | 16.01157813279890 |

**GaL-I**

| | | | |
|---|---|---|---|
| Ga | 2.81283514407374 | 6.69502244039906 | 15.94345388666171 |
| I | 1.89885307944550 | 7.45780105830618 | 13.52176612038274 |
| O | 3.86703770257037 | 8.31975904766405 | 16.10525216130830 |
| N | 1.11555016500675 | 7.75945222162130 | 17.06447555281133 |
| C | 2.50575775248059 | 7.34218612050759 | 18.99640943312711 |
| N | 3.29101363687519 | 6.40180079121548 | 18.18657391899350 |
| C | 5.19065094655289 | 8.03298216213588 | 18.12839913203392 |
| C | 5.24535331898853 | 10.08108734995827 | 16.79825329093441 |
| C | 1.15832996489234 | 7.63314160743165 | 18.40234411389747 |
| C | 6.15442495304373 | 10.61881719721146 | 17.70333110897977 |
| C | 6.09583423038943 | 8.62760231653807 | 19.01965209352770 |
| C | 4.73965544894772 | 8.75832226983025 | 16.99333613404608 |
| C | 4.74838504013543 | 6.62134731634253 | 18.37961688637743 |
| C | 6.60276806789555 | 9.91311678468690 | 18.83277336785966 |
| C | 2.96048997348836 | 4.99066816222549 | 18.52235313248481 |
| C | 0.75411528219510 | 4.31674559480508 | 19.55523835223325 |
| C | 1.49514974447901 | 4.70057792885089 | 18.43133344222154 |
| C | -1.17068391565250 | 8.19831890477119 | 18.58770607500226 |
| C | -0.03888605790113 | 8.10421631601204 | 16.47525739854177 |
| C | 0.86521907283340 | 4.82047459164619 | 17.16604750285813 |
| C | 0.02511699244524 | 7.83728112321419 | 19.19684583880255 |

| | | | |
|---|---|---|---|
| C | -0.61161479270912 | 4.03892577019397 | 19.47851682211292 |
| C | 4.76827657106208 | 10.84119317018661 | 15.59581709518646 |
| C | -1.20200619710278 | 8.34705605808634 | 17.19957412611892 |
| C | 7.59288486592280 | 10.52101754846263 | 19.79211633209988 |
| O | 1.51359317622484 | 5.20516449069883 | 16.08597005220649 |
| C | -0.52670155960323 | 4.51910081975456 | 17.07045214734210 |
| C | -1.22306340131565 | 4.14421789839725 | 18.21586378848423 |
| C | -1.18402207969024 | 4.62495383562319 | 15.72623641593577 |
| C | -1.40323033710534 | 3.62363010640388 | 20.69173641531609 |
| H | -0.79485158047163 | 3.69766754199544 | 21.60481247230023 |
| H | -1.75826918061672 | 2.58198450318098 | 20.61579078164236 |
| H | -2.29601903166139 | 4.25453537297530 | 20.83303557421699 |
| H | 8.57633357235004 | 10.68460535763730 | 19.31972736063321 |
| H | 7.75139940724846 | 9.86724799513406 | 20.66216997562204 |
| H | 7.25330941448841 | 11.50028678306663 | 20.16809525563683 |
| H | 5.23458506961178 | 11.83554667368982 | 15.54459496615146 |
| H | 3.67300296053161 | 10.95535653277238 | 15.60934378205188 |
| H | 4.98818741598825 | 10.28788787780970 | 14.66995090079804 |
| H | 6.41674457277612 | 8.04822335367293 | 19.89284028192081 |
| H | 6.52818864851881 | 11.63339463658833 | 17.52463345379437 |
| H | 1.26696618318522 | 4.23740103911165 | 20.52049811491649 |
| H | -0.98462855403280 | 5.60350866354381 | 15.26446394109179 |
| H | -2.26975158619393 | 4.46873108588900 | 15.80029268426727 |
| H | -0.76525366810155 | 3.88586305193270 | 15.02500858972233 |
| H | -2.29442112541134 | 3.93087913133974 | 18.12645642154980 |
| H | 5.24887905502164 | 5.92361113266802 | 17.68712289162917 |
| H | 5.02206727372189 | 6.32755564054717 | 19.41375973333771 |
| H | 3.07571827273903 | 8.28607954516444 | 19.03337282620796 |
| H | 2.39433750480060 | 6.97693267282319 | 20.03415460888613 |
| H | 3.33123175748877 | 4.77128773053292 | 19.54375303865045 |
| H | 3.52666410931409 | 4.37400127586926 | 17.80368432013756 |
| H | 0.08938032092648 | 7.67899902220859 | 20.27395838644656 |
| H | -2.07205258179789 | 8.34650621430084 | 19.18534916201594 |
| H | -2.11745619311645 | 8.62449263637793 | 16.67673133624559 |
| H | 0.00059433323657 | 8.16053400104948 | 15.38265720667855 |
| I | 4.80918902188699 | 5.01769173633640 | 15.13052486765766 |

**GaL cation**

| | | | |
|---|---|---|---|
| Ga | 2.63775592357212 | 6.85242508222714 | 16.70284493540132 |
| O | 4.04231545469139 | 7.71140678013761 | 15.95325514572236 |
| N | 1.24707989100245 | 7.95207431977494 | 17.66955593805986 |
| C | 2.60431878256978 | 7.19341546866662 | 19.54446297333040 |
| N | 3.29289075222240 | 6.33218462126252 | 18.55125741271427 |
| C | 5.13696998530368 | 7.96389673555030 | 18.12312359885263 |
| C | 5.15332257915246 | 9.76345439745955 | 16.46528481321441 |
| C | 1.32234056929189 | 7.78279491957513 | 19.00934498927354 |
| C | 5.90576497779306 | 10.52739416888988 | 17.36096071296527 |
| C | 5.88216119505880 | 8.77564982455811 | 18.99190500674722 |
| C | 4.76475783359554 | 8.46798302974364 | 16.85805202229574 |
| C | 4.78772733283499 | 6.55957919300451 | 18.52746509608551 |
| C | 6.28298366950997 | 10.06321022534950 | 18.63002999010290 |

| | | | |
|---|---|---|---|
| C | 2.93197904075958 | 4.87386412587626 | 18.70284098587556 |
| C | 0.64164222069513 | 4.08546062407114 | 19.40383215946251 |
| C | 1.48212773653500 | 4.60729160136994 | 18.41134818773246 |
| C | -0.82690215285452 | 8.82679124753573 | 19.26443907497487 |
| C | 0.16970813272383 | 8.53977108004329 | 17.10734625786740 |
| C | 0.96624473021852 | 4.84122688741615 | 17.11837673210780 |
| C | 0.28456688300813 | 8.21095165279958 | 19.83431154483267 |
| C | -0.69509419072394 | 3.77730262099363 | 19.14090549701203 |
| C | 4.76587881076252 | 10.28373758872073 | 15.11167368419468 |
| C | -0.88423759922536 | 9.00259698371670 | 17.87881746248992 |
| C | 7.10569028315541 | 10.91979603745783 | 19.55315005555883 |
| O | 1.75653331199222 | 5.37027073847346 | 16.11801774941740 |
| C | -0.37834528923978 | 4.54367017770496 | 16.82403849013087 |
| C | -1.17486518710623 | 4.01528358333658 | 17.84365332599723 |
| C | -0.91587542485578 | 4.79548040404879 | 15.44547380997896 |
| C | -1.58602614909541 | 3.18115010477083 | 20.19648655938975 |
| H | -1.13319264630910 | 3.25320504313524 | 21.19395517498868 |
| H | -1.77590552854206 | 2.11565661498968 | 19.99503747304225 |
| H | -2.56469759513092 | 3.68110318026697 | 20.22645788317289 |
| H | 8.11305944562251 | 11.08694500552369 | 19.14277875478994 |
| H | 7.22065491178386 | 10.45284323674408 | 20.53981940472161 |
| H | 6.64834653607058 | 11.90983122109603 | 19.69494409551632 |
| H | 5.13658843623889 | 11.30518567703852 | 14.96429905583881 |
| H | 3.67270080060150 | 10.28819517540155 | 14.98154768577848 |
| H | 5.16944156504613 | 9.64645081152255 | 14.31142680040410 |
| H | 6.17656452543753 | 8.37440951770813 | 19.96592323993716 |
| H | 6.20832800385391 | 11.53211899657552 | 17.05355421034636 |
| H | 1.05297219424483 | 3.89310857095229 | 20.39898670255000 |
| H | -0.82304823069554 | 5.85680012091588 | 15.16533585578064 |
| H | -1.97273799947567 | 4.51082177528411 | 15.37741151433347 |
| H | -0.35140388398453 | 4.23043076294968 | 14.68975199467979 |
| H | -2.21799974075600 | 3.78035602869744 | 17.61479566214321 |
| H | 5.18461106863057 | 5.82770039909479 | 17.80624413983209 |
| H | 5.20637039433947 | 6.31954137097818 | 19.51694584224368 |
| H | 3.28564335081091 | 8.03003160078367 | 19.77638071901512 |
| H | 2.41308661789638 | 6.63730576137414 | 20.47379745840347 |
| H | 3.19648069123816 | 4.54787537857976 | 19.72083337966576 |
| H | 3.57722771939109 | 4.33363224882782 | 17.99262777302383 |
| H | 0.35118773924230 | 8.05438289137003 | 20.91099529369231 |
| H | -1.64939056666179 | 9.16358521179087 | 19.89629385825692 |
| H | -1.73847308399889 | 9.47923675017996 | 17.40052938275596 |
| H | 0.17178288595785 | 8.62223171328376 | 16.01984717479708 |

**GaL – IC**

| | | | |
|---|---|---|---|
| Ga | 2.62884928461244 | 6.89575357589081 | 16.15749786630876 |
| I | 1.97797330756614 | 7.44344301394253 | 13.63521469708442 |
| O | 3.83662357737591 | 8.38011917005925 | 16.19039161483752 |
| N | 1.04430633623791 | 7.85384700456915 | 17.19499681120785 |
| C | 2.48299084294743 | 7.48325456289166 | 19.10790908054510 |
| N | 3.23226335347415 | 6.51071736571867 | 18.29164896388584 |
| C | 5.22500154669520 | 8.05284010784652 | 18.16194043006494 |

| | | | |
|---|---|---|---|
| C | 5.38023260202009 | 10.05084263539929 | 16.76879512382947 |
| C | 1.12268465190655 | 7.77795535073646 | 18.53802006410359 |
| C | 6.39831967028072 | 10.51727695878542 | 17.59579162235414 |
| C | 6.24114835451349 | 8.57448646558944 | 18.97623503441364 |
| C | 4.78167184004646 | 8.78746501901458 | 17.03815303498290 |
| C | 4.70341824326578 | 6.67938598971805 | 18.46141115304484 |
| C | 6.85672524155294 | 9.79604001305751 | 18.71055518813825 |
| C | 2.87994973482101 | 5.10944876656214 | 18.67050962775675 |
| C | 0.61477542891263 | 4.46402928307055 | 19.60336713901358 |
| C | 1.42464670068436 | 4.81023795791018 | 18.51580662381131 |
| C | -1.19551027838602 | 8.38045577879605 | 18.73230603044556 |
| C | -0.11431103295314 | 8.18888063439122 | 16.60395122268831 |
| C | 0.88378148385878 | 4.88121519175720 | 17.21335291734543 |
| C | 0.00708371888450 | 8.03034712503218 | 19.33770819359417 |
| C | -0.74224202054774 | 4.18342337638027 | 19.43656024387173 |
| C | 4.89984829267298 | 10.82951847936601 | 15.57914725734219 |
| C | -1.25668554888300 | 8.47479006832419 | 17.34060048631414 |
| C | 7.97766035005335 | 10.31880153505976 | 19.56960162538096 |
| O | 1.64369935310548 | 5.22886265646225 | 16.17161834514151 |
| C | -0.49262444043029 | 4.59232053110891 | 17.02263253293901 |
| C | -1.26552320893823 | 4.25481882934784 | 18.13344611534518 |
| C | -1.06539556724178 | 4.66625515734042 | 15.63814899324747 |
| C | -1.61655274730356 | 3.79594452154084 | 20.60038635930876 |
| H | -1.09985888351339 | 3.95434033315365 | 21.55720062476368 |
| H | -1.90632247307356 | 2.73384967835959 | 20.55379225181776 |
| H | -2.54904908996696 | 4.38076658294786 | 20.61923601595980 |
| H | 7.83169666124227 | 11.37853673175851 | 19.82792158941545 |
| H | 8.94970911104087 | 10.24473466799181 | 19.05461425177826 |
| H | 8.05932434676537 | 9.75120056184942 | 20.50691030948984 |
| H | 5.44348394794771 | 11.77861054983839 | 15.48210130644063 |
| H | 3.82198687985028 | 11.04023420873403 | 15.65183114776615 |
| H | 5.01916934207474 | 10.25315310496851 | 14.64807740036818 |
| H | 6.56803704955894 | 7.98691050207876 | 19.84027042105173 |
| H | 6.85068383955885 | 11.48754506621100 | 17.36662539523042 |
| H | 1.05950231527276 | 4.41436960497070 | 20.60277881242634 |
| H | -0.82893875858660 | 5.62848912354960 | 15.15926718912540 |
| H | -2.15442576874852 | 4.52535670107685 | 15.65001002905519 |
| H | -0.61966081174112 | 3.89952308068266 | 14.98557398005224 |
| H | -2.32869853059170 | 4.04291328305085 | 17.97992095296139 |
| H | 5.16754584700487 | 5.93141606725706 | 17.80074617684384 |
| H | 4.97496608265678 | 6.40460628600201 | 19.49886938264792 |
| H | 3.06886401061545 | 8.41843300395001 | 19.11190163498550 |
| H | 2.39295819004950 | 7.13911770198949 | 20.15292285018321 |
| H | 3.20703637266211 | 4.93392753838514 | 19.71258608219919 |
| H | 3.47654783913713 | 4.46347406770216 | 18.00757096558308 |
| H | 0.08681842471005 | 7.92588760630276 | 20.41977481341053 |
| H | -2.08156133593976 | 8.56889719029720 | 19.34006806853616 |
| H | -2.17873726050136 | 8.74533060055809 | 16.82773367699537 |
| H | -0.09365026641498 | 8.20474708376058 | 15.51113265393601 |
| C | 7.46600759087895 | 7.43109651602760 | 15.42150819457993 |
| C | 6.78236482013550 | 6.20976758465037 | 15.36148446688738 |
| C | 7.36222415647828 | 5.07312394160632 | 15.94390661000011 |

| | | | |
|---|---|---|---|
| H | 6.83409236343409 | 4.11792881213717 | 15.92195459239544 |
| C | 8.60217882997891 | 5.16067578031293 | 16.57390143858153 |
| H | 9.04443001787233 | 4.27175491433265 | 17.02658162365047 |
| C | 9.27697812592659 | 6.38427361590607 | 16.62973843268410 |
| H | 10.24442081868188 | 6.45131959916753 | 17.12990476973375 |
| C | 5.43034082842623 | 6.18897446022143 | 14.75732650921763 |
| C | 4.68003337598541 | 4.98789648648603 | 14.35392215680757 |
| H | 5.12802268383454 | 3.99706078358470 | 14.46159032045057 |
| H | 3.89583620899604 | 5.10128160513109 | 13.59965629321344 |
| O | 4.37112922703863 | 5.61623814346311 | 15.62152542917716 |
| H | 5.09870365903525 | 7.12722767585587 | 14.30322189319854 |
| C | 8.70692323127504 | 7.51922680136905 | 16.05190724596652 |
| H | 9.21769058532255 | 8.48135684886838 | 16.10687754700665 |
| H | 7.00303671622435 | 8.32366783088145 | 14.99835569487317 |

**GaL – TS1**

| | | | |
|---|---|---|---|
| I | 0.16977013958689 | 5.92676103335762 | 13.30623730778195 |
| Ga | 6.07221494775217 | 3.91077592114564 | 13.04320671373251 |
| I | 5.23830337555312 | 3.82502488590937 | 15.57182793113073 |
| O | 7.14359347046409 | 5.48540243599266 | 13.41573936207979 |
| N | 7.77903219097002 | 2.53351939756732 | 13.24320143938897 |
| C | 8.34277699127707 | 3.35395053002859 | 11.04044449882164 |
| N | 6.99384443608270 | 3.93626448083946 | 10.96986720253852 |
| C | 7.96992809166961 | 6.22354631611097 | 11.25653252295960 |
| C | 8.80982626017879 | 7.13978702645133 | 13.35830031112783 |
| C | 8.44774153956718 | 2.29434542989820 | 12.09973531164562 |
| C | 9.64888948404148 | 7.97655723536932 | 12.62787172400612 |
| C | 8.84435581707301 | 7.07484494724037 | 10.56646788545602 |
| C | 7.94118479538498 | 6.23884592515452 | 12.67358946961216 |
| C | 7.03221659284877 | 5.34562691435753 | 10.48453663554977 |
| C | 9.69002833649661 | 7.96822297848215 | 11.22332679237048 |
| C | 6.11366812299457 | 3.13633378127204 | 10.07062298130334 |
| C | 6.53688236515265 | 0.68717801940418 | 9.59426777120833 |
| C | 6.06391104452126 | 1.68991032055844 | 10.44893213591184 |
| C | 9.36529707503936 | 0.26864949463926 | 13.01039987758086 |
| C | 7.88642060143379 | 1.67453919299657 | 14.26892539051666 |
| C | 5.51376101831613 | 1.35552823175107 | 11.71077585638194 |
| C | 9.24779470180168 | 1.15804980064292 | 11.94883847098323 |
| C | 6.48298709304290 | -0.66163887256351 | 9.94851959227404 |
| C | 8.76513858941120 | 7.16059832797057 | 14.85790137365367 |
| C | 8.68193267279052 | 0.53665854580867 | 14.19899032585170 |
| C | 10.60787745651118 | 8.89163017685406 | 10.46505295746799 |
| O | 5.08896467999276 | 2.28062962961502 | 12.55311697239449 |
| C | 5.43145587578131 | -0.01910326006242 | 12.07628549188403 |
| C | 5.91865852822232 | -0.98273059431832 | 11.19679359748799 |
| C | 4.82324872554347 | -0.37089504435529 | 13.40166551601522 |
| C | 6.98072215368194 | -1.74135850690343 | 9.02262997847526 |
| H | 7.52567460423508 | -1.31168360571601 | 8.16984072574495 |
| H | 6.15250951902864 | -2.34420757004661 | 8.61433965569949 |
| H | 7.65944516330655 | -2.43947521620932 | 9.53810261169405 |
| H | 11.66112899052562 | 8.75618278058437 | 10.76037445789403 |

H 10.35956637655095 9.95081083437127 10.64341732001452
H 10.53970030855893 8.71449866471662 9.38222106515513
H 9.48350228011541 7.88525810836161 15.26584962071108
H 8.98026229472085 6.16403531193132 15.27332084419291
H 7.75620779109870 7.41283990270109 15.21964317308902
H 8.84668101333553 7.04015697874691 9.47169881696680
H 10.30186623233751 8.66457835217566 13.17591857999150
H 6.95864047203664 0.97495329745094 8.62484963477936
H 5.30257479616688 0.18781634790144 14.21937112671520
H 4.90133432870801 -1.44860309506239 13.60196172086961
H 3.76181085260434 -0.07983087508789 13.43595713388642
H 5.86222963389518 -2.03513176702956 11.49621738494023
H 5.99745801944851 5.71433669103140 10.55495153280341
H 7.31954149694956 5.34911340686066 9.41488431964702
H 9.03327143520920 4.17329009907095 11.30349186804495
H 8.65669523990894 2.94880188078508 10.06200791675242
H 6.47550809412496 3.24935994766563 9.03015326792625
H 5.11485771873408 3.59362438136365 10.14360237487825
H 9.73952110101460 0.97106786016490 10.99390026063985
H 9.97061765552038 -0.63361217202642 12.90848536908260
H 8.74189013579477 -0.13554741068067 15.05459662479324
H 7.28568722495161 1.92637941692702 15.14811983237798
C 5.08744971478971 8.16513538605330 12.12960008523426
C 3.92211056192417 7.38623393881283 12.08236732253176
C 2.84968518535983 7.79884163522489 11.28241418955812
H 1.91507575483168 7.23123738027525 11.30806282379303
C 2.95729735126729 8.96516346883332 10.52128515081379
H 2.11119680506256 9.28747272393341 9.91086452776149
C 4.13165322841815 9.72161816191205 10.54678490948821
H 4.21222340126018 10.63002565294674 9.94551449832490
C 3.88095866691218 6.11743803549577 12.86972456197916
C 2.94346106837362 5.03202544210530 12.60599051704687
H 2.41688254464193 4.96679337564457 11.66169039995044
H 2.76147255869955 4.28916433680575 13.37597672224513
O 4.57386143126395 5.00473587023685 12.21343855019253
H 4.19098121052160 6.22244551213299 13.91767397937578
C 5.19830012121725 9.32039676828895 11.35658033344475
H 6.11993639380663 9.90485142607225 11.38890279850292
H 5.91742327368194 7.85080834605757 12.76404772236898

**GaL – Int1**

Ga 3.06749534978486 6.87649948344155 16.50259398381656
I 0.90446021578702 9.40207777782767 12.81887579145977
O 4.69454730866259 7.79432897280655 16.34741232703707
N 1.42332924942951 7.79944047336354 17.36493982855883
C 2.61047431269790 7.68348172173094 19.47989244576083
N 3.50883402034367 6.72340568760685 18.83318040711454
C 5.31467349671140 8.41335738238041 18.59095766008915
C 5.56427999534086 9.97803856246889 16.73878241144750
C 1.33627495947012 7.88542487807213 18.70684133235313
C 6.08011294434501 10.90742332550694 17.64318971496146

| | | | |
|---|---|---|---|
| C | 5.82727001485698 | 9.38026269778535 | 19.46581238695042 |
| C | 5.16556253754968 | 8.70601978358302 | 17.21951857015596 |
| C | 4.94235505411805 | 7.04826353775030 | 19.07147898417545 |
| C | 6.22049530139027 | 10.64093553445803 | 19.01415408381556 |
| C | 3.23965518111745 | 5.33432955224249 | 19.29922548557821 |
| C | 0.88154460350630 | 4.50210866157716 | 19.77428370868810 |
| C | 1.89856230469158 | 4.83107697683456 | 18.87254230355961 |
| C | -0.98301331839506 | 8.49633868153391 | 18.53358591217151 |
| C | 0.35020621186517 | 8.06181681490454 | 16.59099128205821 |
| C | 1.69140599205676 | 4.69601701362934 | 17.48539066060987 |
| C | 0.13266705022950 | 8.22799913280228 | 19.32171722753220 |
| C | -0.35710382381572 | 4.03535312385256 | 19.32664883275435 |
| C | 5.42700280735908 | 10.29374650145107 | 15.27799927717950 |
| C | -0.87070146369654 | 8.42394527542866 | 17.14434767212031 |
| C | 6.78298115387630 | 11.67647495209567 | 19.95211445149762 |
| O | 2.67169832883790 | 5.02930981285257 | 16.62132054164406 |
| C | 0.44380880130576 | 4.23123133907922 | 17.00754198516990 |
| C | -0.54618400913899 | 3.91198061622786 | 17.93996698853379 |
| C | 0.22154989590947 | 4.11920196125378 | 15.52714602201893 |
| C | -1.45112181907385 | 3.65916917546396 | 20.29159171224324 |
| H | -1.21444366992005 | 3.99157037807718 | 21.31184339462959 |
| H | -1.60152186827140 | 2.56824355666265 | 20.32641166466880 |
| H | -2.41501532764109 | 4.10631511901291 | 20.00458197345845 |
| H | 6.19050264792242 | 12.60443684342592 | 19.93284272677137 |
| H | 7.81476150060993 | 11.94887793139734 | 19.68027184642605 |
| H | 6.79727930248817 | 11.30918779211598 | 20.98739693913033 |
| H | 5.86458685386593 | 11.27320705422639 | 15.04423626938545 |
| H | 4.36901932712883 | 10.30784154330119 | 14.96992344209241 |
| H | 5.92043360815549 | 9.52758157441562 | 14.66164580070673 |
| H | 5.93286641652012 | 9.12846581146477 | 20.52595572103386 |
| H | 6.38383790982926 | 11.88682067247348 | 17.26035971904571 |
| H | 1.06435310036731 | 4.61139403213827 | 20.84825622574945 |
| H | 0.47516534318291 | 5.06158023471673 | 15.01690793341629 |
| H | -0.81989919783567 | 3.85420703746193 | 15.30165312893805 |
| H | 0.87786718210379 | 3.35241066119181 | 15.08638868039246 |
| H | -1.51408276807508 | 3.56027982183030 | 17.56867755061537 |
| H | 5.51485342274857 | 6.28241314698935 | 18.52420490089290 |
| H | 5.17743375388968 | 6.93906331173684 | 20.14752354041624 |
| H | 3.13516429868378 | 8.65463395275099 | 19.51423057677519 |
| H | 2.37834058892026 | 7.39323475462874 | 20.52068722235638 |
| H | 3.34913763748426 | 5.29462882455725 | 20.39998428132371 |
| H | 4.02695925868327 | 4.70843137019404 | 18.85201758218732 |
| H | 0.08014146758756 | 8.26830657542915 | 20.40989152616457 |
| H | -1.93440583366884 | 8.75562017993147 | 19.00036210879543 |
| H | -1.71608405311611 | 8.63164858509601 | 16.48984128048926 |
| H | 0.52313835060870 | 7.96810420598314 | 15.51681557389401 |
| C | 5.38010122700683 | 6.86990387698737 | 12.55361232524215 |
| C | 4.23543021204958 | 6.48052364472056 | 13.26134257473664 |
| C | 4.04540612959201 | 5.12637467454580 | 13.55859105595561 |
| H | 3.17034735283234 | 4.83004162637563 | 14.13921213803002 |
| C | 4.98832105800864 | 4.17871304016482 | 13.15352019521576 |
| H | 4.83568205556461 | 3.12614418621964 | 13.39998543787119 |

C 6.12489365370921 4.57220166403683 12.44403534802351
H 6.86257395588807 3.83065398797951 12.13242993190261
C 3.20571278574602 7.52576589652913 13.68802876210466
C 2.31568697292746 7.77441996358291 12.47347173089497
H 1.69262629158182 6.90012677199267 12.25421204571203
H 2.88337929722218 8.07840688746859 11.58828140850150
O 2.41039748461207 7.12397905563466 14.76180443758047
H 3.75923281927240 8.45712998862420 13.91159982227289
C 6.32027881490828 5.92392127012829 12.14575070108240
H 7.21310826044551 6.24148510644834 11.60385733605883
H 5.54176432295697 7.92904506863720 12.33418610679801

**GaL – Int1-I**

I 2.06607238311705 7.68323220615781 14.55298365045380
Ga 5.94942219388839 3.78781969696434 12.98069782405960
I 5.16923742250288 3.57893947752067 15.58306994702258
O 7.28781114166065 5.14950352026731 13.43771996567773
N 7.65954364776905 2.33932382503769 13.19740067079045
C 8.31544059673618 3.29710433099773 11.07234649528116
N 6.95096451653620 3.81841676691200 10.94280177424303
C 7.72819450417891 6.19852293004752 11.28584421588523
C 8.48478155916988 7.16632554718023 13.39740919796875
C 8.40823732400878 2.19309490646559 12.09043656004352
C 9.04760791903401 8.22271511859469 12.68767227896066
C 8.32862956161221 7.27381984414300 10.61820643440493
C 7.79338198666856 6.12588851632051 12.70469076260353
C 6.93783381667229 5.22181379739584 10.46188848357172
C 8.98049879911331 8.30839078708968 11.28800007736393
C 6.12900143048421 2.99807802340010 10.01349458423287
C 6.58455640002209 0.55200203737613 9.55228827370886
C 6.07401110666190 1.55132236939909 10.38813691408648
C 9.36502454803799 0.16259153257362 12.95188662187092
C 7.74643778558352 1.43368297886274 14.18091927865463
C 5.46131102249387 1.21418733349600 11.62282057864675
C 9.27008036546488 1.10349476763092 11.93247912778264
C 6.51258270232258 -0.79934092843054 9.89471375231602
C 8.54822653319427 7.08564827973753 14.89459743706956
C 8.59791207106340 0.33556851687200 14.10534756972769
C 9.54209302559132 9.49267595029826 10.54599036633046
O 4.99845244000942 2.13217688813919 12.44518323158283
C 5.36297727788585 -0.16561896810302 11.97335180700556
C 5.88884667924997 -1.12579208779031 11.11289929998814
C 4.69987716697508 -0.52199568337216 13.27086408516725
C 7.06530301602138 -1.87383830225593 8.99405822529204
H 7.58037341593867 -1.43554650704652 8.12691127697563
H 6.27262493753092 -2.53606074472460 8.60718143279907
H 7.78857314097375 -2.51777850391367 9.52101791428569
H 10.43945560117178 9.89408748109837 11.04148625234674
H 8.80990393055712 10.31686308375481 10.48306904275056
H 9.81638926200847 9.22688022542464 9.51405319026784
H 9.06420057200481 7.95949156508929 15.31752442079781

| | | | |
|---|---|---|---|
| H | 9.06551231771947 | 6.16987025453320 | 15.22179481362086 |
| H | 7.53832553374130 | 7.01539397906375 | 15.32827067493806 |
| H | 8.25279323481669 | 7.31053237817938 | 9.52573135845101 |
| H | 9.55748964033398 | 9.01504134464488 | 13.24721998114757 |
| H | 7.05461340856832 | 0.84504371293670 | 8.60651653846018 |
| H | 5.14182893788783 | 0.03956477892724 | 14.10784085634915 |
| H | 4.77310362102323 | -1.59996291877885 | 13.47398944486567 |
| H | 3.63674573115346 | -0.23366239373198 | 13.26210340601996 |
| H | 5.81966058662590 | -2.17973631619144 | 11.40591336351826 |
| H | 5.87636815818585 | 5.51370356141047 | 10.45149615005190 |
| H | 7.30690366445793 | 5.24711164140423 | 9.41573410072808 |
| H | 8.94716412111290 | 4.13582834800877 | 11.41023647307362 |
| H | 8.71015716658223 | 2.95053397835041 | 10.09882452178819 |
| H | 6.52674362079743 | 3.11468876867117 | 8.98512224582582 |
| H | 5.12081931217966 | 3.44139355051105 | 10.05038693708408 |
| H | 9.82935522677740 | 0.99129572558033 | 11.00284971703520 |
| H | 10.01908456198706 | -0.70448045642615 | 12.84248386819566 |
| H | 8.63733562680168 | -0.37826636067079 | 14.92822817531597 |
| H | 7.08839595574035 | 1.61679001950228 | 15.03632627976596 |
| C | 5.26155910726393 | 8.41499454435943 | 12.36849434614066 |
| C | 4.46577092881418 | 7.32216192465504 | 12.01288258373212 |
| C | 3.88529797539670 | 7.30092747007227 | 10.73735730703848 |
| H | 3.28480912758296 | 6.43623194613651 | 10.44617767707070 |
| C | 4.09850548865617 | 8.34692076729535 | 9.83915383605328 |
| H | 3.64275604211867 | 8.31401969957952 | 8.84664575326519 |
| C | 4.90144061383424 | 9.43257166134634 | 10.20413807794658 |
| H | 5.08068090636794 | 10.24555891934469 | 9.49709116412972 |
| C | 4.28665462998300 | 6.14259113760548 | 12.95468074465989 |
| C | 2.85161254692148 | 5.95977756453258 | 13.44772796904150 |
| H | 2.15302260222603 | 5.81340748833499 | 12.61681746187490 |
| H | 2.80707821087674 | 5.12794460655172 | 14.15839796078325 |
| O | 4.58130802372092 | 4.93419792851965 | 12.29727725298300 |
| H | 4.93303230422271 | 6.30764637860672 | 13.83823637000399 |
| C | 5.48297955664315 | 9.46265514202422 | 11.47264175759359 |
| H | 6.12918039982030 | 10.29393668723159 | 11.76172052197250 |
| H | 5.73661852934136 | 8.42693667266787 | 13.35084701725668 |

**GaL – TS2**

| | | | |
|---|---|---|---|
| Ga | 3.69637728251968 | 4.86338742931101 | 15.97278636801892 |
| I | 4.05363636260951 | 0.34964840080484 | 15.08062805904359 |
| O | 4.41981548029717 | 6.36002258172190 | 15.03121999338318 |
| N | 1.81360545709886 | 5.80364331780630 | 16.07835761115104 |
| C | 2.97910874073050 | 7.00677331978845 | 17.83299307387367 |
| N | 3.99594812691491 | 5.93260721558248 | 17.82245955614560 |
| C | 5.68673012743678 | 7.48946892373686 | 16.77257172975558 |
| C | 5.66699642684441 | 8.26843853689623 | 14.45978089833586 |
| C | 1.69478176120939 | 6.58773988023853 | 17.16888711670940 |
| C | 6.50514709299087 | 9.31484918580610 | 14.83254847529293 |
| C | 6.51638932066825 | 8.57254498937268 | 17.10215330642930 |
| C | 5.23844733547655 | 7.32731962030010 | 15.43906809331177 |
| C | 5.38657914930193 | 6.48121974783483 | 17.84221899920504 |

| | | | |
|---|---|---|---|
| C | 6.94609248583918 | 9.49839518231319 | 16.15412243849939 |
| C | 3.82098484614828 | 5.01539275634608 | 18.99054134669731 |
| C | 1.59691336271725 | 4.59406292613095 | 20.12316418264719 |
| C | 2.46388478997958 | 4.38923832387010 | 19.04334404985433 |
| C | -0.68893910849665 | 6.67391192197432 | 16.87891857853078 |
| C | 0.72255263852492 | 5.44767795505619 | 15.38088400774460 |
| C | 2.07842636196681 | 3.56127093435539 | 17.96608047086735 |
| C | 0.44462024219094 | 7.03302718485105 | 17.60089476430503 |
| C | 0.34110694921526 | 3.98645909340196 | 20.17044898905347 |
| C | 5.20325689193964 | 8.08569468936221 | 13.04426685130823 |
| C | -0.54875314646922 | 5.87683351169538 | 15.74119859004449 |
| C | 7.83464236735863 | 10.65668980851941 | 16.52430736900406 |
| O | 2.88953296780707 | 3.37788297105680 | 16.91913059981983 |
| C | 0.81193471466660 | 2.92453638742090 | 18.00142646215135 |
| C | -0.02075964157097 | 3.15465568372670 | 19.09641037103514 |
| C | 0.41466308521752 | 2.03228573093026 | 16.86293592166155 |
| C | -0.58444575291192 | 4.18207749840052 | 21.34265659912313 |
| H | -0.61631494610849 | 3.28716750775598 | 21.98485944805586 |
| H | -1.61662661881707 | 4.37775761226416 | 21.01541110631306 |
| H | -0.26215689805644 | 5.02501865443015 | 21.96950842855330 |
| H | 7.32247386664477 | 11.62246908162954 | 16.38609313453828 |
| H | 8.74318552139595 | 10.68517984583054 | 15.90287141565844 |
| H | 8.14984028885811 | 10.59576353403158 | 17.57517538531787 |
| H | 5.61624804620867 | 8.86450379639915 | 12.38962089334033 |
| H | 4.10452371653963 | 8.11328135578914 | 12.97989162484872 |
| H | 5.50066338589339 | 7.09976403140578 | 12.65482161267512 |
| H | 6.84592410549502 | 8.67786363489805 | 18.14094059665257 |
| H | 6.82591425257928 | 10.02285427233998 | 14.06169317142692 |
| H | 1.91662478485723 | 5.24099000148229 | 20.94656327016619 |
| H | 0.38631346618691 | 2.58788858229863 | 15.91246156569741 |
| H | -0.57154981514911 | 1.58198709877180 | 17.03670574890925 |
| H | 1.15429329735841 | 1.23175924708682 | 16.71078757044285 |
| H | -0.99998769514141 | 2.66554174512912 | 19.11501837475662 |
| H | 6.05180665932847 | 5.60716296991681 | 17.75912308041277 |
| H | 5.57441460224433 | 6.93145993565686 | 18.83455020237517 |
| H | 3.40548475776326 | 7.85468995620499 | 17.27065207891081 |
| H | 2.78326574293551 | 7.34790436421847 | 18.86304987734911 |
| H | 4.02516691694634 | 5.58525470587327 | 19.91510812890792 |
| H | 4.60347987879214 | 4.24609521913020 | 18.88705746990395 |
| H | 0.37167297276819 | 7.63617328323215 | 18.50580389430534 |
| H | -1.67571609005104 | 7.00488854434646 | 17.20498433259055 |
| H | -1.41096483776166 | 5.57384346283992 | 15.14865680196520 |
| H | 0.89769123604537 | 4.79733305611423 | 14.52446305263823 |
| C | 7.31023037672906 | 3.19535969684498 | 17.71328665803539 |
| C | 6.97924760785367 | 3.50570284318109 | 16.38829095408170 |
| C | 7.60424893110144 | 4.60567319056735 | 15.77837656462158 |
| H | 7.32739538476683 | 4.86864862179433 | 14.75626129199384 |
| C | 8.52758168890892 | 5.38043518632910 | 16.47570690365162 |
| H | 8.98306800029739 | 6.24520057591028 | 15.99116048069827 |
| C | 8.84608922167503 | 5.06744488672870 | 17.80132583466435 |
| H | 9.56323563098720 | 5.67825337792393 | 18.35193540356605 |
| C | 5.92747300859906 | 2.75422940302714 | 15.57141143762845 |

| | | | |
|---|---|---|---|
| C | 5.34571326402177 | 1.56081425750683 | 16.32178218734251 |
| H | 4.70309656091768 | 1.87308095480005 | 17.15107155491847 |
| H | 6.12951235711001 | 0.87349451359659 | 16.65759389062572 |
| O | 4.95617426799864 | 3.67580339619709 | 15.13472909581646 |
| H | 6.42064852790000 | 2.37740551120521 | 14.65332986916392 |
| C | 8.23776499131274 | 3.97288808091076 | 18.41613272394742 |
| H | 8.48009296740878 | 3.72059144260720 | 19.45005285766290 |
| H | 6.84207246977960 | 2.34737065849797 | 18.21493609865182 |
| O | 3.01011490977772 | 4.18904189202763 | 13.80459689654053 |
| C | 4.00429690211653 | 3.62743754789833 | 13.41282706373330 |
| O | 4.70882861475902 | 3.11936475435552 | 12.61670709253451 |

**GaL – Int2**

| | | | |
|---|---|---|---|
| Ga | 3.73397896230292 | 5.15560663549916 | 16.75574199877742 |
| I | 4.68374907032422 | 0.02904416980757 | 17.26209487561434 |
| O | 5.42834436756946 | 5.91541093962176 | 16.58222517057738 |
| N | 1.95282451918342 | 6.15982033001988 | 16.35819876208813 |
| C | 2.43297289813816 | 7.49677658940134 | 18.32591010000203 |
| N | 3.48192300098401 | 6.52520375856359 | 18.65309214169367 |
| C | 5.30527972481111 | 7.97404101068055 | 17.80561370382315 |
| C | 6.11380843311585 | 7.93278693930480 | 15.50092351275045 |
| C | 1.45198239147594 | 6.95937121732960 | 17.32260020361958 |
| C | 6.32231290465937 | 9.31076344214851 | 15.59545095052305 |
| C | 5.52124195160101 | 9.35683965045982 | 17.85437162537462 |
| C | 5.59997001115418 | 7.25552269946811 | 16.63107690867565 |
| C | 4.77205274206484 | 7.20241941398936 | 18.97033015876194 |
| C | 6.03882819434425 | 10.04837040408315 | 16.75639705122553 |
| C | 3.07711070065371 | 5.66094201839983 | 19.79844027546109 |
| C | 0.65061678577765 | 5.02186279711141 | 20.18692038923160 |
| C | 1.85655474061157 | 4.85130464722484 | 19.49756028093987 |
| C | -0.72506159027856 | 6.86847375582625 | 16.30606866488686 |
| C | 1.15397745474948 | 5.71925339002360 | 15.36265469530629 |
| C | 1.95538762074843 | 3.89661425691841 | 18.46555719010713 |
| C | 0.10553779833835 | 7.32322951455017 | 17.32469694142011 |
| C | -0.47657582382786 | 4.25984275898346 | 19.87222142553791 |
| C | 6.40891526399248 | 7.16167372927395 | 14.24715968567768 |
| C | -0.18859307627132 | 6.06478814501565 | 15.29845517209193 |
| C | 6.29891689244823 | 11.53095629897031 | 16.81237551773324 |
| O | 3.11274376682003 | 3.72905903767954 | 17.79389052553645 |
| C | 0.82479430985611 | 3.11802317042716 | 18.12292190629347 |
| C | -0.35928692740832 | 3.31890237260424 | 18.83531620444415 |
| C | 0.93123404135558 | 2.12792053433250 | 17.00115915671441 |
| C | -1.76878808820044 | 4.41733156877146 | 20.63006476306680 |
| H | -1.77375172542846 | 5.34184493325636 | 21.22402574542802 |
| H | -1.93222507467165 | 3.57846410306306 | 21.32552015130156 |
| H | -2.63463901031716 | 4.44440517087615 | 19.95161052190460 |
| H | 5.86505352784518 | 12.05015984082670 | 15.94451199948176 |
| H | 7.37854880153042 | 11.75011797623222 | 16.81111005149020 |
| H | 5.87210391330714 | 11.97690591533901 | 17.72119335216708 |
| H | 6.74630324032417 | 7.83044135270953 | 13.44461797412422 |
| H | 5.52146772681096 | 6.61048947203991 | 13.90060885630079 |

| | | | |
|---|---|---|---|
| H | 7.18474477324455 | 6.40048665336219 | 14.41867065446227 |
| H | 5.28992003629420 | 9.89942738543695 | 18.77652680293928 |
| H | 6.71653098012411 | 9.83533304363142 | 14.71951318222809 |
| H | 0.59619785254853 | 5.76578863842955 | 20.98825555686300 |
| H | 1.17726993890163 | 2.62854458444789 | 16.05088408648107 |
| H | -0.00605351253448 | 1.57091739613773 | 16.87253507273428 |
| H | 1.75302375587152 | 1.41836842928350 | 17.17801110628933 |
| H | -1.23396575584750 | 2.71848536106237 | 18.56593606326982 |
| H | 5.47221475817466 | 6.39838617363725 | 19.24548277330087 |
| H | 4.64220153222495 | 7.85634089802350 | 19.85316879352801 |
| H | 2.92750369690008 | 8.37223285454804 | 17.86964746451310 |
| H | 1.89951377034304 | 7.84350302694554 | 19.22878474796899 |
| H | 2.91948003873433 | 6.29522269961597 | 20.69128856938379 |
| H | 3.93167643434488 | 4.99522103326929 | 19.99459308844325 |
| H | -0.27844593380518 | 7.94292851450881 | 18.13495427751410 |
| H | -1.78340825005104 | 7.13252499313260 | 16.30072392043510 |
| H | -0.80201389709615 | 5.68980414489032 | 14.48045346693805 |
| H | 1.63846920684218 | 5.06827675764742 | 14.63462743364795 |
| C | 8.44654813925269 | 2.06478933341646 | 14.28287394385587 |
| C | 7.57480856923872 | 2.70567156861596 | 15.17083693061650 |
| C | 7.96352736240085 | 3.91261117885726 | 15.76513516638018 |
| H | 7.26766787041738 | 4.43692017429918 | 16.42181693761752 |
| C | 9.21055207905688 | 4.46721177983813 | 15.47145737913098 |
| H | 9.50099509897964 | 5.41440856936881 | 15.93025445025192 |
| C | 10.08057014343989 | 3.82098000756536 | 14.58991503341277 |
| H | 11.05351037475757 | 4.25841168883609 | 14.35966778867709 |
| C | 6.24517655940710 | 2.06060733868115 | 15.51589871424735 |
| C | 6.41127889864097 | 1.25631216094157 | 16.79326979138059 |
| H | 6.55657194185830 | 1.91007474081671 | 17.66041055628049 |
| H | 7.24726874504609 | 0.55596083620094 | 16.70611197463835 |
| O | 5.22951341770891 | 3.04604594491862 | 15.75443483948146 |
| H | 5.92013675035081 | 1.42173476611745 | 14.68155937465198 |
| C | 9.69451499538346 | 2.61815600923119 | 13.99349311138833 |
| H | 10.36209697108024 | 2.11540121125360 | 13.29167031396107 |
| H | 8.13614109178010 | 1.13391768873582 | 13.80226947233919 |
| O | 3.65222202739241 | 4.39104315009914 | 14.98415753919609 |
| C | 4.47819794022695 | 3.43334803841683 | 14.64885126210593 |
| O | 4.59605609467124 | 2.92021231504511 | 13.55296242798737 |

**GaL – TS3**

| | | | |
|---|---|---|---|
| Ga | 3.12238812350712 | 5.03754038114170 | 10.34321680729765 |
| I | 4.96930922691235 | -0.15137233267204 | 15.56211494788431 |
| O | 2.68859444423328 | 5.74332887027685 | 12.00031042874863 |
| N | 4.10996311655205 | 6.13409413449513 | 8.89427446949710 |
| C | 1.95259728033087 | 7.19760971978175 | 8.57297977798341 |
| N | 1.38889653764725 | 6.12061898353846 | 9.40090016472121 |
| C | 1.23462924690286 | 7.57018156208126 | 11.41356643522083 |
| C | 3.01067103511119 | 7.85080279795004 | 13.06469964613526 |
| C | 3.33084961350012 | 6.86856138911309 | 8.07279013189920 |
| C | 2.59752363442720 | 9.17478443739977 | 13.22362893027697 |
| C | 0.85918317178434 | 8.90769641788162 | 11.59706755550740 |

| | | | |
|---|---|---|---|
| C | 2.31625626023122 | 7.03681772138059 | 12.14069269383645 |
| C | 0.50203272833444 | 6.66392908579304 | 10.47749313142444 |
| C | 1.52728564582669 | 9.73179217697287 | 12.50483660282052 |
| C | 0.62121025902718 | 5.14399838924512 | 8.56850628497942 |
| C | 1.30631436685798 | 4.53961480087647 | 6.20021104536299 |
| C | 1.48247699074546 | 4.42021872721298 | 7.58375122169801 |
| C | 5.14207180252558 | 7.08325607885952 | 6.50701254155905 |
| C | 5.38995646016893 | 5.86883944674946 | 8.55710984284997 |
| C | 2.48708225736922 | 3.57798037886264 | 8.09981449569554 |
| C | 3.82437313351601 | 7.35241520939035 | 6.86171061614058 |
| C | 2.10819276567450 | 3.82863893705170 | 5.30519896537485 |
| C | 4.15614266936074 | 7.26956708704533 | 13.84095357540037 |
| C | 5.94325619961337 | 6.33820938413169 | 7.37463185258226 |
| C | 1.10898087953615 | 11.16147384928044 | 12.72593283677465 |
| O | 2.67506973385684 | 3.46877747035610 | 9.43483747108421 |
| C | 3.30913301218249 | 2.84347663068074 | 7.21454498819064 |
| C | 3.09753343945419 | 2.98739302207668 | 5.84192081403568 |
| C | 4.37559709768172 | 1.94908248870932 | 7.77467212358830 |
| C | 1.91202360462983 | 3.93595519266209 | 3.81575089434744 |
| H | 1.18466979143879 | 4.71984433582106 | 3.56431269648722 |
| H | 1.54092851180274 | 2.99043441793009 | 3.39026366495979 |
| H | 2.85532439053926 | 4.17072291441083 | 3.29944117636919 |
| H | 1.96767819647541 | 11.84695694926367 | 12.66724041546762 |
| H | 0.65694038786138 | 11.29715072811019 | 13.72109218690059 |
| H | 0.36895469031563 | 11.48120987232341 | 11.97977437028918 |
| H | 4.61550049310799 | 8.02391492521097 | 14.49239682044515 |
| H | 4.92798003342129 | 6.86509981987422 | 13.16834387786050 |
| H | 3.82278012184657 | 6.42507368637340 | 14.46270814826822 |
| H | 0.01365834204055 | 9.30427713385365 | 11.02632401208202 |
| H | 3.13811627783168 | 9.80304093356343 | 13.93800948174774 |
| H | 0.51627685099131 | 5.19397581403821 | 5.81829829992288 |
| H | 5.10461173766871 | 2.52291491223702 | 8.36918879719689 |
| H | 4.91305868164008 | 1.42448646777413 | 6.97406790817250 |
| H | 3.94629789353496 | 1.20648986452222 | 8.46334466577619 |
| H | 3.73613134753014 | 2.42006836087507 | 5.15778216982208 |
| H | 0.11705638609539 | 5.78456425540849 | 11.01691127708207 |
| H | -0.35732346454367 | 7.18234796022696 | 10.01541431940757 |
| H | 2.03369245690814 | 8.09356473579502 | 9.21364583950788 |
| H | 1.29005944039100 | 7.44467352898425 | 7.72626788231818 |
| H | -0.20060930841404 | 5.67956722507865 | 8.05950909447311 |
| H | 0.17773998875742 | 4.42724748411478 | 9.27668586015606 |
| H | 3.16607292086702 | 7.92079623401717 | 6.20475687210841 |
| H | 5.54034441538803 | 7.44477789148401 | 5.55818335732361 |
| H | 6.97998423451139 | 6.10599419801337 | 7.13566583002186 |
| H | 5.94460419502230 | 5.25698275339284 | 9.26759562874571 |
| C | 2.45256999453889 | -0.21473805572197 | 12.57569322334759 |
| C | 3.63917844015657 | 0.45590827333334 | 12.26390023279099 |
| C | 4.39293793458858 | 0.03627382945379 | 11.16037712143327 |
| H | 5.32228657103273 | 0.55038782556403 | 10.90581075133593 |
| C | 3.96689077868262 | -1.04150149078829 | 10.38447190103115 |
| H | 4.56632923021898 | -1.36764174782046 | 9.53260842443124 |
| C | 2.78072701318663 | -1.70729790405733 | 10.70202561061706 |

| | | | |
|---|---|---|---|
| H | 2.45120857775477 | -2.55528387030297 | 10.09940422046080 |
| C | 4.06221574476966 | 1.64377995806722 | 13.08004466799877 |
| C | 5.53128504779714 | 1.72111379392731 | 13.44573794517386 |
| H | 5.81132448893123 | 2.28255624821273 | 14.33200179577298 |
| H | 6.19120046405567 | 0.90728931477343 | 13.16640981865836 |
| O | 3.79288647601568 | 2.88386883629837 | 12.23885579211856 |
| H | 3.42330400750105 | 1.78077753223624 | 13.95520669185225 |
| C | 2.02464968188462 | -1.29151045841422 | 11.80032743510957 |
| H | 1.10488226468285 | -1.81659294602409 | 12.06232990413609 |
| H | 1.88301719337340 | 0.09338813163077 | 13.45398494239261 |
| O | 4.89033196235499 | 4.37659507870835 | 10.98711270136691 |
| C | 4.93060994729434 | 3.39798634098197 | 11.80164938148356 |
| O | 6.02529777484851 | 2.88816166561915 | 12.21982322585728 |

**GaL – FC**

| | | | |
|---|---|---|---|
| Ga | 3.06873026672702 | 5.11830322839417 | 10.22268807928405 |
| O | 2.73789035185345 | 5.74256189642204 | 11.91948480720781 |
| N | 4.14682171346060 | 6.20302410752126 | 8.87613281829105 |
| C | 1.97943354046585 | 7.24268466800157 | 8.49948695121855 |
| N | 1.41790183237119 | 6.13927983342502 | 9.30404938906908 |
| C | 1.22665673173930 | 7.54541212199020 | 11.35193744476480 |
| C | 2.97765242889453 | 7.84288716499873 | 13.02622357104814 |
| C | 3.39162286424517 | 6.96991776986518 | 8.05813419887147 |
| C | 2.52697203082943 | 9.15234387645843 | 13.20664633804678 |
| C | 0.81477159935275 | 8.86976704277510 | 11.55692561173582 |
| C | 2.31537981717227 | 7.03131929399967 | 12.08137737695979 |
| C | 0.51185061296688 | 6.64955814705520 | 10.39072919519513 |
| C | 1.45029267752858 | 9.69452003522067 | 12.48715929360482 |
| C | 0.67969847490146 | 5.15176162470408 | 8.44654179691242 |
| C | 1.43380329843413 | 4.54763148000232 | 6.10437600993313 |
| C | 1.57781305916753 | 4.42975835299414 | 7.49144223590881 |
| C | 5.27884892378686 | 7.31552385268997 | 6.60964085282324 |
| C | 5.44892576782933 | 5.98209598417650 | 8.59189215763630 |
| C | 2.57837293973807 | 3.59566964011717 | 8.02660169458747 |
| C | 3.93753595577466 | 7.53539203396936 | 6.90730650278388 |
| C | 2.26321037495028 | 3.84403025819139 | 5.22734575540681 |
| C | 4.13231363564465 | 7.28421590003857 | 13.80655947084388 |
| C | 6.05101755178221 | 6.53293685077430 | 7.47129157720922 |
| C | 0.99397962739505 | 11.10821793601078 | 12.72817104461729 |
| O | 2.73511984881198 | 3.49282077224148 | 9.38159468575014 |
| C | 3.43500773477669 | 2.87888607623003 | 7.16591060131662 |
| C | 3.25292561486743 | 3.02095782012684 | 5.78705876925522 |
| C | 4.51468586155921 | 2.00244308619748 | 7.73373411869550 |
| C | 2.08980014365620 | 3.93858694716753 | 3.73530904437690 |
| H | 1.46334013785417 | 4.79627946747792 | 3.45663776462038 |
| H | 1.60693667228443 | 3.03381720338863 | 3.33431723049924 |
| H | 3.05702107907462 | 4.03990037507630 | 3.22241424525604 |
| H | 1.83062132006640 | 11.81861738233538 | 12.65582082815242 |
| H | 0.56334288457193 | 11.22047669765099 | 13.73498945432712 |
| H | 0.22748217759883 | 11.41153611278879 | 12.00308536948247 |
| H | 4.55769402344584 | 8.04119553647659 | 14.47695863581909 |

| | | | |
|---|---|---|---|
| H | 4.92809362839716 | 6.92277371205310 | 13.13697292314336 |
| H | 3.81848663447266 | 6.42011886090622 | 14.41155124110870 |
| H | -0.03647618299484 | 9.25396206873689 | 10.98719879174748 |
| H | 3.04234969561400 | 9.78042152086214 | 13.93885937609192 |
| H | 0.64537730502305 | 5.19164382446191 | 5.70366245518863 |
| H | 5.14384389688041 | 2.55439418636096 | 8.44910332773656 |
| H | 5.15688280386252 | 1.60518684644516 | 6.93788781532930 |
| H | 4.09266516795467 | 1.15071708942328 | 8.28839210156564 |
| H | 3.91862926866839 | 2.46767712474039 | 5.11828928067030 |
| H | 0.13061394702837 | 5.75219868573214 | 10.90249683394932 |
| H | -0.34661506411168 | 7.16696789178212 | 9.93062229936890 |
| H | 1.99751180345054 | 8.13966686754176 | 9.14349093221584 |
| H | 1.34290804561192 | 7.46344307632575 | 7.62820967296119 |
| H | -0.12608435750890 | 5.68282173700680 | 7.91248137921902 |
| H | 0.21533071646184 | 4.43804244453879 | 9.14393159845657 |
| H | 3.30492504058933 | 8.13467609286421 | 6.25258580889993 |
| H | 5.71911511719893 | 7.74623138416029 | 5.70970526742891 |
| H | 7.10410973807855 | 6.33868362052817 | 7.27392399797133 |
| H | 5.98705821341995 | 5.34193590173687 | 9.28890243190294 |
| C | 3.49753279193390 | -0.81356979883010 | 13.00336020298174 |
| C | 3.49182960166870 | 0.42009998354018 | 12.33787829374759 |
| C | 2.96640943457397 | 0.51216485599667 | 11.04539588951140 |
| H | 2.92733142406680 | 1.47187487841070 | 10.52754568747812 |
| C | 2.46008416974363 | -0.63194118048295 | 10.42254620053328 |
| H | 2.04324169390324 | -0.55458465096368 | 9.41705739542574 |
| C | 2.47796251939219 | -1.86273707938440 | 11.07888893398978 |
| H | 2.08010934972094 | -2.75185454829263 | 10.58842381650324 |
| C | 4.09757742158486 | 1.60506666232972 | 13.04579547377379 |
| C | 5.64475341990126 | 1.63541052283165 | 13.05405323671686 |
| H | 6.06853618547833 | 1.97867928707751 | 14.00684930968087 |
| H | 6.09102705923814 | 0.68242240099523 | 12.75164539566922 |
| O | 3.78059716883457 | 2.85706213123320 | 12.35479506267669 |
| H | 3.69194822991740 | 1.70629344332054 | 14.06129405511755 |
| C | 2.99643079165730 | -1.95168729440755 | 12.37357300379162 |
| H | 3.00217301117354 | -2.90774269262469 | 12.89816416130484 |
| H | 3.88884891595201 | -0.88786193172355 | 14.02145985891602 |
| O | 4.87953287795740 | 4.26945838224615 | 10.97339857638836 |
| C | 4.87101647515102 | 3.30860679388386 | 11.74475232693764 |
| O | 5.97443864277401 | 2.62774898788200 | 12.04010568438587 |

**GaL – FC-I**

| | | | |
|---|---|---|---|
| Ga | 2.01714942657187 | 4.62285591229397 | 10.16371709305772 |
| O | 1.99141455587437 | 5.62690037830565 | 11.77074888208600 |
| N | 3.78403399660789 | 5.32950068815107 | 9.22529573501917 |
| C | 2.36205933711954 | 7.19126659341555 | 8.61754767324740 |
| N | 1.21093042479116 | 6.38061195323401 | 9.06122412663608 |
| C | 1.03658823472876 | 7.76010487686256 | 11.13306070866079 |
| C | 2.48969694397488 | 7.52296320656427 | 13.07884660425523 |
| C | 3.58598447167895 | 6.35570731000032 | 8.37307549610905 |
| C | 2.32965296219684 | 8.88482430352218 | 13.32497492104119 |
| C | 0.91675940683159 | 9.12948790710960 | 11.41165214944482 |

| | | | |
|---|---|---|---|
| C | 1.83249008339044 | 6.93930509192084 | 11.96210790855441 |
| C | 0.31194578625412 | 7.15757334117766 | 9.96985926973933 |
| C | 1.54804869780561 | 9.71812228500098 | 12.50715312940602 |
| C | 0.42411772999248 | 5.89247867672806 | 7.88755117639819 |
| C | 1.50015573121601 | 5.56504289412863 | 5.61558229842139 |
| C | 1.27133795715401 | 5.12481094045223 | 6.92440162779395 |
| C | 5.66974612687361 | 5.88269970033007 | 7.27437126179343 |
| C | 4.89593051923655 | 4.57957550583577 | 9.12979571650759 |
| C | 1.85723983968624 | 3.92642139462374 | 7.38737547252034 |
| C | 4.51586201313665 | 6.65182843730819 | 7.37445465662813 |
| C | 2.30500014746339 | 4.83975655381432 | 4.73532351165206 |
| C | 3.32822750022121 | 6.64249913987554 | 13.95819459846992 |
| C | 5.87197568997978 | 4.83466073956135 | 8.17585617386651 |
| C | 1.39995334083713 | 11.18608634558806 | 12.81036739514417 |
| O | 1.67129020803700 | 3.50004713369467 | 8.63789935874390 |
| C | 2.67060398740544 | 3.16892920280365 | 6.50368569741652 |
| C | 2.87432222924379 | 3.64432715588678 | 5.20905369734298 |
| C | 3.28024645680630 | 1.88958994844330 | 6.99625017951180 |
| C | 2.53812628929326 | 5.29896739571220 | 3.31972384754054 |
| H | 2.19267065374485 | 6.33183899089862 | 3.17329610420311 |
| H | 2.00049576860382 | 4.66586682772390 | 2.59543237246470 |
| H | 3.60461027715006 | 5.25740234464181 | 3.05041931914871 |
| H | 2.37810969132478 | 11.68732081197803 | 12.87902141156134 |
| H | 0.88736211084160 | 11.35130965937407 | 13.77146372473724 |
| H | 0.81617336972854 | 11.69581959908048 | 12.03145252550561 |
| H | 3.78742841092460 | 7.21832873450610 | 14.77273093408659 |
| H | 4.12109314961430 | 6.14462678581305 | 13.37928319586394 |
| H | 2.72523582938048 | 5.82921878372510 | 14.38992307666203 |
| H | 0.30129174816433 | 9.74672021397759 | 10.74899978455680 |
| H | 2.84165801491948 | 9.31908571498342 | 14.18974882662665 |
| H | 1.03448712190778 | 6.49759339108811 | 5.28016323040002 |
| H | 3.81798254305158 | 2.04349115061173 | 7.94376550237201 |
| H | 3.96850019527773 | 1.46523127581175 | 6.25303434046189 |
| H | 2.50135520061802 | 1.14437024163620 | 7.22072965726776 |
| H | 3.51064424237487 | 3.06022201549756 | 4.53615593108027 |
| H | -0.45683318352120 | 6.44591960107244 | 10.30417691534088 |
| H | -0.18912290304676 | 7.95098547368690 | 9.38469892551528 |
| H | 2.58845904359170 | 7.90158748341307 | 9.43112449948356 |
| H | 2.11182562063471 | 7.77526191284134 | 7.71613210096405 |
| H | -0.04166129188191 | 6.76373718437277 | 7.39105243563254 |
| H | -0.37289015766782 | 5.25495878758339 | 8.29844850367572 |
| H | 4.31221646311363 | 7.46196872040189 | 6.67438438863907 |
| H | 6.40164995257323 | 6.08962996893500 | 6.49244891348343 |
| H | 6.76131993184768 | 4.20745017989442 | 8.13005086047258 |
| H | 4.96257985038593 | 3.74905270448277 | 9.83717935707803 |
| C | -2.05586799026294 | -0.78379684987149 | 13.23650014491279 |
| C | -1.12012259159414 | 0.10905865795658 | 12.70327783857523 |
| C | 0.09522174614034 | -0.37786425009570 | 12.20154480624165 |
| H | 0.82473070082665 | 0.31346855923727 | 11.77118075251953 |
| C | 0.36364564294596 | -1.74488536854918 | 12.23842988097242 |
| H | 1.30814994989372 | -2.11785803311339 | 11.83954369905207 |
| C | -0.56863210923927 | -2.63316937036633 | 12.78314476019249 |

| | | | |
|---|---|---|---|
| H | -0.35273536764905 | -3.70239322820585 | 12.81189393392618 |
| C | -1.41204518263900 | 1.57993104315660 | 12.70081615821500 |
| C | -0.48987164169854 | 2.43595248646965 | 13.58216008216779 |
| H | -0.92822971619585 | 2.69366774733793 | 14.55244768033188 |
| H | 0.50205318481138 | 1.97812774153210 | 13.71071719876436 |
| O | -1.17894867525617 | 2.16536355358868 | 11.37840527094533 |
| H | -2.47231093441278 | 1.75767664624606 | 12.94213980813640 |
| C | -1.77908104793355 | -2.15148119658449 | 13.28375903347299 |
| H | -2.51346373125339 | -2.84076278793835 | 13.70306632219735 |
| H | -3.00880763350007 | -0.40556550275955 | 13.61467271668452 |
| O | -0.34146136839393 | 4.11034280398546 | 10.59417752396626 |
| C | -0.58660298096964 | 3.36657021982818 | 11.52538891517183 |
| O | -0.32234412093119 | 3.64936854435602 | 12.81506014147178 |
| I | 2.93631079684648 | 2.50923922171278 | 11.52669386028901 |

**InCl – catalyst**

| | | | |
|---|---|---|---|
| In | 2.54316500756834 | 7.10631093024354 | 16.06722922137309 |
| Cl | 1.56078342118499 | 8.24133602339690 | 14.18684561969889 |
| O | 4.52318663446072 | 7.65970194311653 | 15.80901967779651 |
| N | 1.04692630863217 | 7.98289317826077 | 17.58366721609472 |
| C | 2.59938853218955 | 7.23272248333377 | 19.30089962118560 |
| N | 3.30430415614307 | 6.40569206147761 | 18.31307910795545 |
| C | 5.22748223526779 | 7.98157820527644 | 18.08704678708215 |
| C | 5.50861133141456 | 9.74722373462477 | 16.42279583988098 |
| C | 1.20840668768584 | 7.62078114754051 | 18.87105459823225 |
| C | 6.09811944698871 | 10.53295881844396 | 17.41411891316163 |
| C | 5.82001798009655 | 8.80768744211913 | 19.05172294405114 |
| C | 5.06445558876000 | 8.44480652588877 | 16.76267604269492 |
| C | 4.78439526208636 | 6.58916086447628 | 18.40988329646726 |
| C | 6.26751995685517 | 10.09226149334289 | 18.73705099828893 |
| C | 2.97277311923313 | 4.95911785601399 | 18.47588223901181 |
| C | 0.72662001075977 | 4.25866277784114 | 19.40245157918824 |
| C | 1.51234074519457 | 4.67193792391994 | 18.31962014294661 |
| C | -1.09563847017818 | 8.14074965017466 | 19.32592866834822 |
| C | -0.14722878357991 | 8.42333498380273 | 17.14599334743608 |
| C | 0.93109909664394 | 4.84179524577179 | 17.04174810851955 |
| C | 0.14198656020793 | 7.68614016211763 | 19.76964510936632 |
| C | -0.64208816014382 | 4.02459915255380 | 19.26413520943353 |
| C | 5.32785974477299 | 10.23564250211210 | 15.01524672291549 |
| C | -1.24285449260597 | 8.52899576297354 | 17.99281955174949 |
| C | 6.91833335023515 | 10.97529738221288 | 19.76921363088196 |
| O | 1.66700944619611 | 5.21422155780859 | 15.98213241332667 |
| C | -0.46173310714313 | 4.61983262077995 | 16.88436157198753 |
| C | -1.20843772809666 | 4.22036359393746 | 17.99300918234237 |
| C | -1.08439713514333 | 4.83994085309525 | 15.53717978856885 |
| C | -1.48506617143940 | 3.56354168833125 | 20.42399804788177 |
| H | -0.93956083698252 | 3.65092332275209 | 21.37389888350110 |
| H | -1.78448845310367 | 2.50914538016223 | 20.31191507695221 |
| H | -2.41186886089590 | 4.15121950019509 | 20.50966218191948 |
| H | 7.96905985735965 | 11.18606350460714 | 19.51495824291426 |
| H | 6.90486675757614 | 10.50423710537954 | 20.76166778910980 |

| | | | |
|---|---|---|---|
| H | 6.40723462095460 | 11.94704513645668 | 19.85057619093727 |
| H | 5.66627297784968 | 11.27494903692769 | 14.91119107398766 |
| H | 4.27419906710982 | 10.16822765020845 | 14.70377984429561 |
| H | 5.88961977675604 | 9.60928117942163 | 14.30570585098273 |
| H | 5.94589962285758 | 8.42615881479255 | 20.07030139797465 |
| H | 6.43561798491724 | 11.53881347783020 | 17.14480803172634 |
| H | 1.20230880234778 | 4.12312023031541 | 20.37933647963178 |
| H | -0.87923272259020 | 5.85658307363786 | 15.16600441965482 |
| H | -2.17078129746625 | 4.68459568787662 | 15.57231257554390 |
| H | -0.65413801229055 | 4.16005099768730 | 14.78652448616558 |
| H | -2.28305672733689 | 4.05706504975892 | 17.86247692586315 |
| H | 5.22126213652541 | 5.87965863506327 | 17.69082943351214 |
| H | 5.12010012175808 | 6.29594283527089 | 19.42279762668434 |
| H | 3.18513416296363 | 8.16098842726499 | 19.41796080023325 |
| H | 2.55949369314965 | 6.73342834433709 | 20.28599512801234 |
| H | 3.32924010206397 | 4.62412438355368 | 19.46876250304482 |
| H | 3.55019963093847 | 4.42506731282820 | 17.70601850950096 |
| H | 0.28750963623189 | 7.35745096028520 | 20.79860015412817 |
| H | -1.94275416402985 | 8.18264869634121 | 20.01190338505904 |
| H | -2.19498086458065 | 8.88939787926244 | 17.60562270692066 |
| H | -0.19209259153030 | 8.67186285539473 | 16.08291446377513 |

**In catalyst cation**

| | | | |
|---|---|---|---|
| In | 2.68815177259308 | 6.86971290772138 | 16.24920137245909 |
| O | 4.43249306194332 | 7.76414471752411 | 15.75480036857691 |
| N | 1.15077191739989 | 7.96344964197169 | 17.50203449539620 |
| C | 2.61412862026945 | 7.19664441407981 | 19.30992544096529 |
| N | 3.33848964678492 | 6.32945585791406 | 18.35544219616449 |
| C | 5.21441700592373 | 7.96355562022129 | 18.05838005811309 |
| C | 5.37213621618497 | 9.82936155232782 | 16.47645876286697 |
| C | 1.26092929956056 | 7.64625607585622 | 18.81264333249295 |
| C | 6.00655037049714 | 10.57345233129921 | 17.47376253193330 |
| C | 5.84443339774965 | 8.75720615528754 | 19.02664425553213 |
| C | 4.98342279116245 | 8.50386536044428 | 16.77208532508529 |
| C | 4.82709143138567 | 6.54984432110183 | 18.39636111545721 |
| C | 6.25794473562575 | 10.06504959987369 | 18.75617361900222 |
| C | 2.98424248882833 | 4.87930846538659 | 18.52870659298822 |
| C | 0.72724291486702 | 4.22417880921532 | 19.42544133367701 |
| C | 1.51479762459875 | 4.62392162383364 | 18.34013712303213 |
| C | -1.01881722225556 | 8.32629588286745 | 19.17991048076872 |
| C | -0.00820400467085 | 8.45359815548447 | 17.01572806420755 |
| C | 0.91289671276566 | 4.79655967270171 | 17.07128411500320 |
| C | 0.17674328473714 | 7.81327862122769 | 19.67451467770621 |
| C | -0.64682720609060 | 3.99979501550452 | 19.29504019376170 |
| C | 5.11588258041731 | 10.39579374930359 | 15.11110893434486 |
| C | -1.11190816935115 | 8.66575831437968 | 17.82801000160724 |
| C | 6.96173171213687 | 10.89592315014646 | 19.79290270084739 |
| O | 1.64351623403780 | 5.14172089780955 | 15.96895758147392 |
| C | -0.47872476161078 | 4.60151027152765 | 16.91777152943478 |
| C | -1.22192965056508 | 4.20396703657202 | 18.03175480993959 |
| C | -1.11634918913139 | 4.81915669940829 | 15.57797329564597 |

| | | | |
|---|---|---|---|
| C | -1.47730675446064 | 3.52866268010318 | 20.45659614643083 |
| H | -0.97194942558784 | 3.70752740019413 | 21.41480728341189 |
| H | -1.67236315189979 | 2.44716539070591 | 20.38499435052966 |
| H | -2.45570635317406 | 4.02880781427048 | 20.48216967303743 |
| H | 8.01670948775854 | 11.05286298535226 | 19.52048357223394 |
| H | 6.94065713136961 | 10.41402847575543 | 20.77878741145859 |
| H | 6.50384947479914 | 11.89119860252828 | 19.88719038389782 |
| H | 5.42924120565560 | 11.44522002733869 | 15.05658895240121 |
| H | 4.04860657077160 | 10.33569575635786 | 14.84654269708378 |
| H | 5.65604971833868 | 9.82952288946021 | 14.33843821609278 |
| H | 6.03439852683462 | 8.32932144853790 | 20.01518283746735 |
| H | 6.31396341062696 | 11.59615905555030 | 17.23930034758161 |
| H | 1.20555542306883 | 4.07489944995906 | 20.39781594590118 |
| H | -0.92726900430361 | 5.83933146046276 | 15.20595815418579 |
| H | -2.20059597265720 | 4.66183707373617 | 15.62665075429709 |
| H | -0.69711793869503 | 4.13900927225529 | 14.82255173934901 |
| H | -2.29664240165151 | 4.04497645362714 | 17.90735505470542 |
| H | 5.25867310500778 | 5.83922879936185 | 17.67422103489574 |
| H | 5.20573982410524 | 6.27829035490900 | 19.39564927132160 |
| H | 3.23398636684305 | 8.09756520261594 | 19.45805542696699 |
| H | 2.50548558387218 | 6.69630318982376 | 20.28515882378167 |
| H | 3.30558519925567 | 4.55414767911326 | 19.53242029800167 |
| H | 3.57893590536659 | 4.32318256158317 | 17.78768390950447 |
| H | 0.27587101331917 | 7.52726324870460 | 20.72157153191207 |
| H | -1.87561391671128 | 8.45563774597730 | 19.84189539671290 |
| H | -2.02830923946476 | 9.07363202354935 | 17.40393268040117 |
| H | -0.03639635888177 | 8.67205029887475 | 15.94711776915180 |

**InCl2 – catalyst**

| | | | |
|---|---|---|---|
| In | 2.84967387620130 | 6.63084080942085 | 15.65189383601570 |
| Cl | 1.83191888982303 | 7.42836955760307 | 13.58619031486862 |
| O | 4.11652361006554 | 8.35131162215534 | 15.89437946046570 |
| N | 1.12144699317700 | 7.77163629322897 | 16.95304498966971 |
| C | 2.54631888615752 | 7.30800144206844 | 18.86106797974754 |
| N | 3.32411604317268 | 6.36872121250893 | 18.04774500724301 |
| C | 5.22170914141857 | 8.01338864523628 | 18.03258577381206 |
| C | 5.33531945955031 | 10.13162241200725 | 16.81922960243658 |
| C | 1.18528974825563 | 7.59619282282835 | 18.28427619022582 |
| C | 6.12515136758735 | 10.64924050950574 | 17.84008863917402 |
| C | 6.01066763699269 | 8.58893006952593 | 19.03984284315354 |
| C | 4.85963564009095 | 8.78145982973731 | 16.88778373054957 |
| C | 4.79333242676117 | 6.58150151199808 | 18.17547206749282 |
| C | 6.48507245774962 | 9.89879858420245 | 18.97282929830396 |
| C | 2.99064356674318 | 4.95306702895666 | 18.36590375297414 |
| C | 0.84015800220799 | 4.38060115529321 | 19.54946466181538 |
| C | 1.51617384174124 | 4.68267249163842 | 18.36043480765662 |
| C | -1.15769285368030 | 8.08969235292257 | 18.50573970150884 |
| C | -0.04729232486639 | 8.09696564745188 | 16.38110924928580 |
| C | 0.80577096537201 | 4.75160363587505 | 17.12785497475006 |
| C | 0.05332723244037 | 7.74601436144612 | 19.09418829572894 |
| C | -0.53561799499690 | 4.15012995582427 | 19.58477241365732 |

| | | | |
|---|---|---|---|
| C | 4.95305129408625 | 10.94401505061931 | 15.61701715053052 |
| C | -1.21108860442157 | 8.27864547883317 | 17.12219853839149 |
| C | 7.34951409156671 | 10.48541573067558 | 20.05862854318733 |
| O | 1.38002984677380 | 5.04592247741719 | 15.98796624384655 |
| C | -0.60340430081632 | 4.49859145759378 | 17.15475778972685 |
| C | -1.23092507754304 | 4.21534975814975 | 18.36297900997375 |
| C | -1.35313083953906 | 4.56638290050478 | 15.85709383219656 |
| C | -1.25105306882745 | 3.82306841236896 | 20.87016283960588 |
| H | -0.58659387170696 | 3.95560007643062 | 21.73656731734988 |
| H | -1.61219376877541 | 2.78049825507080 | 20.88955184534024 |
| H | -2.13227461081012 | 4.46750014289261 | 21.02322392911326 |
| H | 8.37102915126546 | 10.70231232040863 | 19.70202115381333 |
| H | 7.43920521390996 | 9.79227484091992 | 20.90798286155270 |
| H | 6.93934005627940 | 11.43344675160772 | 20.44464411760083 |
| H | 5.36432212196034 | 11.96209423986415 | 15.67838057606812 |
| H | 3.85821369575412 | 11.00075418611182 | 15.51248522887061 |
| H | 5.30840912140188 | 10.46644495137540 | 14.69059935991140 |
| H | 6.26344639390532 | 7.97382741446506 | 19.91119719072639 |
| H | 6.47291834991901 | 11.68537333167114 | 17.75444137237442 |
| H | 1.41575890735533 | 4.33599131330735 | 20.48129405479428 |
| H | -1.17294456343266 | 5.52660483561843 | 15.35049348355256 |
| H | -2.43343893743965 | 4.43347289044950 | 16.01355744428488 |
| H | -0.99644507489089 | 3.79592695889749 | 15.15530396253894 |
| H | -2.31355189549120 | 4.04215674472589 | 18.35983037484425 |
| H | 5.25119596643983 | 5.94793446429674 | 17.39724047802799 |
| H | 5.12805739829220 | 6.20052068186555 | 19.16204686630022 |
| H | 3.11504971073115 | 8.25285146307423 | 18.88803433810278 |
| H | 2.44191454448674 | 6.94974515461766 | 19.90250897909456 |
| H | 3.41301838815078 | 4.70559976560874 | 19.36134562296222 |
| H | 3.50717546041850 | 4.34212729392688 | 17.60735694712936 |
| H | 0.12914065281929 | 7.55193857380897 | 20.16452012615118 |
| H | -2.05752265889941 | 8.18867751893519 | 19.11568458404584 |
| H | -2.14260084977267 | 8.53880208075184 | 16.61907606399393 |
| H | -0.01834433491285 | 8.18357903984785 | 15.29131921594622 |
| Cl | 4.61823766089851 | 5.03258370305179 | 15.04607603921405 |

**InCl – IC**

| | | | |
|---|---|---|---|
| In | 2.75473712449999 | 6.90667712075106 | 15.99110950600384 |
| Cl | 2.10779786551559 | 7.40909870110224 | 13.72079835884092 |
| O | 4.32338548100098 | 8.31928553308441 | 16.00329320442575 |
| N | 1.08000935050189 | 7.97914558135829 | 17.16185037833250 |
| C | 2.47877660948896 | 7.52872412644243 | 19.10177215038448 |
| N | 3.28610108888538 | 6.58424848993586 | 18.31119756884229 |
| C | 5.18495237163567 | 8.23414099707196 | 18.26683201850762 |
| C | 5.42086760894762 | 10.22062780558772 | 16.86657957879210 |
| C | 1.12452498378996 | 7.80151119001541 | 18.49705664620782 |
| C | 6.10726054234469 | 10.83870224457701 | 17.90919415711601 |
| C | 5.87195234619652 | 8.90382997589023 | 19.29101958706917 |
| C | 4.94311369304986 | 8.88951099732349 | 17.03424625356014 |
| C | 4.75499320375310 | 6.81317062799720 | 18.47318132038827 |
| C | 6.34938014086512 | 10.20506929360697 | 19.13938127989783 |

| | | | |
|---|---|---|---|
| C | 2.96507037182234 | 5.16691166348333 | 18.66030237255042 |
| C | 0.73356319889261 | 4.58114571758511 | 19.68727483020646 |
| C | 1.50459249363200 | 4.85851449991333 | 18.55113375483807 |
| C | -1.22787079028417 | 8.27362476420248 | 18.65680205329468 |
| C | -0.07924802166309 | 8.28988077292252 | 16.55634809752085 |
| C | 0.90745074931055 | 4.87023137212964 | 17.26590157675727 |
| C | -0.02725563880264 | 7.94266507639991 | 19.27551773231710 |
| C | -0.63598944142735 | 4.32937328000845 | 19.59971695604414 |
| C | 5.17056325491802 | 10.91174167940584 | 15.55798960323408 |
| C | -1.25709963553882 | 8.46087951486949 | 17.27228884498398 |
| C | 7.09730906166229 | 10.90753653784793 | 20.24201232410892 |
| O | 1.61248378271355 | 5.12134577114570 | 16.16742887043914 |
| C | -0.48922974511648 | 4.61603990134091 | 17.16194070825246 |
| C | -1.21922262887929 | 4.35844319753799 | 18.32045734511046 |
| C | -1.12655271180344 | 4.65026952902910 | 15.80448477925397 |
| C | -1.46092159252241 | 4.01500216982962 | 20.82014613879362 |
| H | -0.90399143967986 | 4.22730403053125 | 21.74348928628673 |
| H | -1.75370725553561 | 2.95292230590514 | 20.84932263233435 |
| H | -2.39137089712696 | 4.60297589777680 | 20.84363476900813 |
| H | 6.61856458575798 | 11.86122519209502 | 20.51410504208689 |
| H | 8.13185652270279 | 11.14222457840737 | 19.94470702636632 |
| H | 7.14508808716185 | 10.28684909858678 | 21.14739048044316 |
| H | 5.54136243010332 | 11.94514158762096 | 15.57897696878570 |
| H | 4.09767182275112 | 10.91844032346659 | 15.31335415072455 |
| H | 5.65882704582135 | 10.38083927280077 | 14.72540875088135 |
| H | 6.04381045941300 | 8.37642322545389 | 20.23497395084441 |
| H | 6.46432006077811 | 11.86296245369412 | 17.76006446042655 |
| H | 1.22226273471702 | 4.56928885581230 | 20.66730903577955 |
| H | -0.90484587242123 | 5.59691521478736 | 15.28804874685989 |
| H | -2.21531319332888 | 4.52335073171681 | 15.87294709274383 |
| H | -0.71930681901918 | 3.85943032716896 | 15.15590214921794 |
| H | -2.29459079499229 | 4.17409879644112 | 18.22623970286999 |
| H | 5.24504071398751 | 6.14226138353633 | 17.74913520419949 |
| H | 5.05834931987724 | 6.48062020082094 | 19.48398589484262 |
| H | 3.03957927781366 | 8.47855209667713 | 19.13283852700163 |
| H | 2.35758222506213 | 7.17513499560445 | 20.14070212570514 |
| H | 3.32353274951381 | 4.97138408298992 | 19.68882943977662 |
| H | 3.54891968682121 | 4.54097818168356 | 17.96738690064189 |
| H | 0.02411632748561 | 7.76086192684105 | 20.34883056848286 |
| H | -2.14003680925479 | 8.37058466962701 | 19.24705508720822 |
| H | -2.17942466289491 | 8.71466136431584 | 16.75127887590976 |
| H | -0.03407197921071 | 8.37985147518916 | 15.46824634714339 |
| C | 7.57598859187653 | 6.99510892391843 | 15.46758095314906 |
| C | 6.92564256158757 | 5.83220243437462 | 15.02775296535823 |
| C | 7.57942984855672 | 4.59777919498481 | 15.14629331448746 |
| H | 7.08925626662178 | 3.68313927100332 | 14.80885326527076 |
| C | 8.85923893629406 | 4.52884700844866 | 15.69507976299604 |
| H | 9.36153840152667 | 3.56386915589528 | 15.77947863429902 |
| C | 9.49864055196366 | 5.69157925416988 | 16.13446178576605 |
| H | 10.49946086375833 | 5.63413153774045 | 16.56546252886593 |
| C | 5.54483274084384 | 5.96811952953193 | 14.50739084521579 |
| C | 4.67792293001018 | 4.87279206516902 | 14.04863791338594 |

| | | | |
|---|---|---|---|
| H | 5.03933182771287 | 3.84216959303494 | 14.04114522775239 |
| H | 3.86865546650407 | 5.11235952954789 | 13.35348824160230 |
| O | 4.48708394494229 | 5.40040252219122 | 15.38704190839746 |
| H | 5.27338809089329 | 6.97219546136318 | 14.17184113121916 |
| C | 8.85423507032309 | 6.92509012603100 | 16.02051256317892 |
| H | 9.34319117227995 | 7.83600359031362 | 16.36875367327419 |
| H | 7.05909268221249 | 7.95359985383494 | 15.39530967283454 |

**InCl – TS1**

| | | | |
|---|---|---|---|
| I | 0.05924770253926 | 6.25867706518395 | 13.75883688505655 |
| In | 5.95651338347194 | 3.94528569985655 | 13.20789989240176 |
| Cl | 5.35028655678532 | 3.72513286243710 | 15.53266673877210 |
| O | 7.02269998034677 | 5.77335760281711 | 13.47719772485769 |
| N | 7.82559626201026 | 2.49638972291213 | 13.30973006777953 |
| C | 8.33886446153176 | 3.35826277532200 | 11.09857609755358 |
| N | 6.99756022267872 | 3.95244453274045 | 11.01707490418680 |
| C | 8.01171310938528 | 6.22938477141517 | 11.31108443999246 |
| C | 8.86802287502249 | 7.23176172994806 | 13.36784051613412 |
| C | 8.43232961342963 | 2.26458442097313 | 12.13066770812737 |
| C | 9.82060365509913 | 7.90777107518800 | 12.61109151462015 |
| C | 8.99719115652696 | 6.92568400826109 | 10.59527045815448 |
| C | 7.93106385024324 | 6.36778007205828 | 12.72294040942771 |
| C | 7.02709951368723 | 5.37617567821894 | 10.56862106065917 |
| C | 9.91220217980262 | 7.77603262019429 | 11.21501154742186 |
| C | 6.10359829634342 | 3.16448558999256 | 10.11941797328695 |
| C | 6.64083796657480 | 0.75780265642657 | 9.58549096957343 |
| C | 6.08335138713039 | 1.70465789349788 | 10.45422523382069 |
| C | 9.27114966600272 | 0.16252411661277 | 12.94204743443763 |
| C | 7.91520408057254 | 1.59566621242885 | 14.30125748110630 |
| C | 5.49874411758085 | 1.29501053435983 | 11.68326848120460 |
| C | 9.16776308561521 | 1.09415973311264 | 11.91592023428605 |
| C | 6.66042667111742 | -0.60315257768847 | 9.89192497702026 |
| C | 8.77325635890064 | 7.38850073354355 | 14.85701155442054 |
| C | 8.64173488593907 | 0.41848302738610 | 14.16348148837308 |
| C | 10.95169250557790 | 8.52996049634903 | 10.42688108251226 |
| O | 4.97106520228685 | 2.15052454827040 | 12.53432198055465 |
| C | 5.50126175558953 | -0.09782253755938 | 11.99917051968627 |
| C | 6.08056044609985 | -0.99925148855814 | 11.11121800534439 |
| C | 4.88076939753711 | -0.53294144237017 | 13.29361752097691 |
| C | 7.24968715247875 | -1.61811630252943 | 8.94687747056343 |
| H | 7.82378961819093 | -1.12902745180543 | 8.14668184123860 |
| H | 6.46968783589099 | -2.23050044373897 | 8.46402451857667 |
| H | 7.92520829229357 | -2.31552348332581 | 9.46750011619469 |
| H | 11.97251563297348 | 8.31161328967416 | 10.78070216455003 |
| H | 10.81272232271498 | 9.62081585595167 | 10.50558871175412 |
| H | 10.90552199654577 | 8.26744809029593 | 9.36028130722516 |
| H | 9.56497057084593 | 8.04897047338828 | 15.23742268117972 |
| H | 8.84153125043884 | 6.41246147574338 | 15.36145933701284 |
| H | 7.79467969472139 | 7.80050893873509 | 15.14898946929543 |
| H | 9.03507197958550 | 6.79939314914827 | 9.50776705861167 |
| H | 10.52501080553498 | 8.56660302040053 | 13.13086750855869 |

| | | | |
|---|---|---|---|
| H | 7.07957216671340 | 1.10348348400885 | 8.64277278309966 |
| H | 5.31551100759031 | 0.01423908660467 | 14.14386534068214 |
| H | 5.00932287345249 | -1.61268845780208 | 13.45350187185144 |
| H | 3.80570173178077 | -0.29601279792000 | 13.31381003115398 |
| H | 6.08512326367646 | -2.06203931433296 | 11.37824086144423 |
| H | 5.99950187049757 | 5.75317144432156 | 10.69706055486399 |
| H | 7.26029355583247 | 5.40670301688596 | 9.48619363588850 |
| H | 9.02845042648902 | 4.16822692927698 | 11.39127111422463 |
| H | 8.66864239436639 | 2.97303119753145 | 10.11652101633156 |
| H | 6.44154273332820 | 3.30892047416888 | 9.07420673733207 |
| H | 5.09998094759678 | 3.60631759603579 | 10.22167152117092 |
| H | 9.61259603540373 | 0.91138103977314 | 10.93771640297870 |
| H | 9.82104244403428 | -0.76700838646513 | 12.78617403057770 |
| H | 8.69057985399894 | -0.29138980745012 | 14.98882200782702 |
| H | 7.35814039526322 | 1.84468024092677 | 15.20842786908151 |
| C | 4.92400056707055 | 8.18523012977265 | 11.96250435678560 |
| C | 3.71912531497827 | 7.47761913213622 | 12.08557618576791 |
| C | 2.58969107059217 | 7.91090492645132 | 11.38139093399078 |
| H | 1.63508289923839 | 7.40042800736874 | 11.53713783456380 |
| C | 2.67604115665464 | 9.02760197695898 | 10.54642633645558 |
| H | 1.78733636923801 | 9.36658996626509 | 10.01004997781306 |
| C | 3.88447342667598 | 9.71388182159765 | 10.40456400273981 |
| H | 3.94712518117539 | 10.58439346771507 | 9.74770511761959 |
| C | 3.69565082994034 | 6.25614311754836 | 12.94736710142948 |
| C | 2.69124673973306 | 5.20677026952014 | 12.82360319473610 |
| H | 2.08893874866858 | 5.11865832108304 | 11.92758983995826 |
| H | 2.53665314186136 | 4.51167423725157 | 13.64173209213402 |
| O | 4.28873576062331 | 5.08136401330635 | 12.30255730780767 |
| H | 4.09006881353066 | 6.41801008087986 | 13.95878865876058 |
| C | 5.01029251344065 | 9.29186955143833 | 11.11836557038338 |
| H | 5.95874282962440 | 9.82397764752177 | 11.02100607486888 |
| H | 5.79856466328537 | 7.85274355555271 | 12.52425227876565 |

**InCl – TS1 open**

| | | | |
|---|---|---|---|
| Cl | 0.17375866159441 | 5.85329571257161 | 13.01870885121700 |
| In | 5.97521827615794 | 3.97367474831641 | 13.15686166785713 |
| Cl | 5.32288031169054 | 3.80729575895502 | 15.47301634694726 |
| O | 7.04725943809018 | 5.79964399250857 | 13.40605956135394 |
| N | 7.82309587665974 | 2.51304989356975 | 13.31415559249789 |
| C | 8.37594472738704 | 3.32889848058655 | 11.09501705873798 |
| N | 7.04104995990454 | 3.93280386494301 | 10.98179822666011 |
| C | 8.07260570481680 | 6.20548290521713 | 11.24660778220974 |
| C | 8.90900767744399 | 7.23730415424326 | 13.29686544694246 |
| C | 8.44541441155253 | 2.25437195184008 | 12.14872921763831 |
| C | 9.88272353099536 | 7.88532552502181 | 12.54273090388774 |
| C | 9.07889430193970 | 6.87443944029235 | 10.53341651029120 |
| C | 7.97177691295984 | 6.37201232481977 | 12.65409550181370 |
| C | 7.09112714407734 | 5.34744906821666 | 10.50516209199996 |
| C | 9.99546644215410 | 7.72434979884686 | 11.15122521129677 |
| C | 6.15442122691932 | 3.13436925994568 | 10.08592328529757 |
| C | 6.67933426434485 | 0.71273555324026 | 9.60989724023636 |

| | | | |
|---|---|---|---|
| C | 6.11622213734877 | 1.68194528733146 | 10.44992132353012 |
| C | 9.25793402473453 | 0.16329337697483 | 13.01339093951860 |
| C | 7.89208983044603 | 1.63142499031359 | 14.32447047261218 |
| C | 5.50848230973922 | 1.30249408226547 | 11.67730910822040 |
| C | 9.17561261048373 | 1.07508796875215 | 11.96773745006093 |
| C | 6.68231903262645 | -0.64160309663467 | 9.94464839343292 |
| C | 8.79314945646469 | 7.42430104163348 | 14.78102180707913 |
| C | 8.61283518054188 | 0.44734783700177 | 14.22040869214600 |
| C | 11.05775854575674 | 8.44856234069027 | 10.36569352497928 |
| O | 4.97464328010724 | 2.17981138689070 | 12.50222323694767 |
| C | 5.49386325108230 | -0.08334883261945 | 12.02195331344558 |
| C | 6.07961243084334 | -1.00768570167576 | 11.16231072735686 |
| C | 4.84873654407722 | -0.48564902003497 | 13.31496151544799 |
| C | 7.27771887403083 | -1.68066930478764 | 9.03021216076625 |
| H | 7.86504832813373 | -1.21256800174270 | 8.22708534405178 |
| H | 6.50027441594376 | -2.29895991616972 | 8.55087313289128 |
| H | 7.94220976629017 | -2.37026370207381 | 9.57491594290143 |
| H | 12.06903164978411 | 8.23019646675843 | 10.74590296319915 |
| H | 10.92752510420034 | 9.54229083328075 | 10.41368527704169 |
| H | 11.03076627328863 | 8.15934285547357 | 9.30535548285123 |
| H | 9.58492571746454 | 8.08543271709623 | 15.16023120756273 |
| H | 8.84568743220637 | 6.45792859201095 | 15.30569470873279 |
| H | 7.81378705927398 | 7.85018116721721 | 15.04918125099798 |
| H | 9.13297700197643 | 6.72565281496861 | 9.44943087652258 |
| H | 10.58749478387921 | 8.54501226041350 | 13.06094235533089 |
| H | 7.13577339373943 | 1.03526255888625 | 8.66739813704979 |
| H | 5.27698829670262 | 0.07333457582404 | 14.16089837127147 |
| H | 4.96230447879237 | -1.56336241023261 | 13.49814087370639 |
| H | 3.77646921930460 | -0.23580817145161 | 13.31379852936100 |
| H | 6.07037649239738 | -2.06473458148167 | 11.45101476836447 |
| H | 6.06517586999458 | 5.73576978081585 | 10.60890763511748 |
| H | 7.34319988590875 | 5.35270189427417 | 9.42665311929104 |
| H | 9.06787990882606 | 4.13873239311988 | 11.38272780036491 |
| H | 8.71690370570711 | 2.92261417489575 | 10.12539731861017 |
| H | 6.50996288268928 | 3.25504131769802 | 9.04364410287229 |
| H | 5.15293264057583 | 3.58592950275573 | 10.16260690013999 |
| H | 9.63323003572223 | 0.87042810989765 | 10.99983798483793 |
| H | 9.80372597725289 | -0.77266013482779 | 12.88380719848446 |
| H | 8.64509545360834 | -0.24630344878063 | 15.06020355278075 |
| H | 7.32348914667262 | 1.90180216833092 | 15.21824258073342 |
| C | 4.83896320173010 | 8.19842087734444 | 11.97747849094386 |
| C | 3.62722740074396 | 7.51227282675408 | 12.14796333082440 |
| C | 2.43842352953110 | 8.05180919305898 | 11.64010530436724 |
| H | 1.49652672944005 | 7.51645881605461 | 11.83147918158158 |
| C | 2.47636303261154 | 9.26681118631340 | 10.94992693354950 |
| H | 1.54825136556572 | 9.68878575436989 | 10.55775263986052 |
| C | 3.68531411242048 | 9.94116084541170 | 10.76100746064426 |
| H | 3.70674372560794 | 10.88851717402801 | 10.21696776143709 |
| C | 3.61868069657344 | 6.21312734826096 | 12.88203734252074 |
| C | 2.76600313223890 | 5.09646642376574 | 12.50053370663503 |
| H | 2.22439455362050 | 5.11864996659036 | 11.56223257546386 |
| H | 2.51420133341630 | 4.33179874124327 | 13.22777335665779 |

O 4.33429232917018 5.12070332282756 12.19330670171054
H 3.89551677031856 6.27249812466074 13.94176473536348
C 4.86909767598876 9.40492540377613 11.27849616853680
H 5.81882721619298 9.92602019004601 11.14030836134659
H 5.75756112152243 7.77761991240077 12.39055510095765

**InCl – Int1**

I 2.35567223264643 8.15910112657421 14.82179876106655
In 5.77395002018395 3.78364421018720 13.21495044873915
Cl 5.18488205899521 3.38273214096126 15.55318593551572
O 7.16399738476562 5.38222676969781 13.64486723016651
N 7.64491351835865 2.29366034986755 13.31660144999719
C 8.28099214350783 3.31817151716501 11.20431968470236
N 6.92773519548398 3.86025180715351 11.06577819822164
C 7.79894334046078 6.21023771495430 11.44561196962336
C 8.60293279508492 7.23064819929854 13.51658585345063
C 8.35419720825813 2.17025059883010 12.18098642387793
C 9.31918698185251 8.15312071912554 12.76159422013303
C 8.55543961024736 7.15313619746998 10.73373513660193
C 7.81316797372307 6.22503754432292 12.87086514163039
C 6.90935873967087 5.29018980655386 10.65809741348988
C 9.31729671915853 8.14042677127179 11.35651037733476
C 6.10326619869600 3.07777005568185 10.10536592002070
C 6.69176235002322 0.69515082017950 9.52400182944113
C 6.08299923911675 1.60842229399340 10.39348461043277
C 9.24995829907427 0.06805332179680 12.93315461797732
C 7.71332306550282 1.34190910370428 14.25814850667909
C 5.42989331590463 1.15428033839550 11.57388312931625
C 9.17077932762143 1.05594951255018 11.95812371530324
C 6.70095339346485 -0.67695274371185 9.77895326075799
C 8.60473305714596 7.25754915060974 15.01706933382724
C 8.51545929083298 0.21432697408924 14.11248433001655
C 10.08875795961240 9.16304701366315 10.56330233355680
O 4.85307138371567 1.97305045587074 12.42227651512434
C 5.42469359201193 -0.25205870365902 11.83507057706051
C 6.05548692323592 -1.11929707647733 10.94821978226343
C 4.74329467808509 -0.73440370191904 13.08127014956247
C 7.35122186805285 -1.65337250992203 8.83305087859810
H 7.96129352996770 -1.13037625682234 8.08207840720685
H 6.60743242804213 -2.25877388323803 8.28716667342799
H 8.00839579529518 -2.36046155741484 9.36479724464932
H 11.14451843789456 9.21585606027159 10.87620112126118
H 9.66960729798368 10.17688791470041 10.68167664321103
H 10.07026831558894 8.92472947471557 9.48986017961278
H 9.23310911393397 8.07534734787896 15.39812624654943
H 8.96566466739852 6.30230948108626 15.42947125768578
H 7.58275847804083 7.37633920100071 15.41015356106058
H 8.52034160896485 7.11980922276267 9.63912815157454
H 9.90577275286126 8.91460634265016 13.28831582699712
H 7.18281782481380 1.07772682975558 8.62181206916854
H 5.13419106264137 -0.21421780073767 13.96923970723729

H 4.86893990164570 -1.81904824080577 13.21028430995482
H 3.66723782946470 -0.50040518119210 13.05838773946035
H 6.05286295257745 -2.19127292521393 11.17771505145824
H 5.85548596347044 5.60185010187097 10.74979341928046
H 7.18500924437221 5.36250160465014 9.58608579976737
H 8.91437482221286 4.13813795211727 11.58378776478097
H 8.69535249302370 3.00103012051463 10.22832979441763
H 6.48879635778253 3.25601928714890 9.08059204717783
H 5.08770481936863 3.50087770479950 10.16955147308232
H 9.70171361295271 0.96001260297010 11.01050961180048
H 9.86453009196441 -0.81876849801186 12.76820468729383
H 8.54354839425219 -0.54073746361424 14.89811950558648
H 7.07990032276204 1.51123998489491 15.13348785220341
C 5.15106206859479 8.40648000137131 11.93608389906279
C 4.19886454615014 7.38245920649468 11.91378316596527
C 3.33109916844721 7.29352021596294 10.81631125035209
H 2.61164191531772 6.47292095617564 10.77699748469722
C 3.40721204522398 8.21379129684690 9.77161275965584
H 2.72423479704755 8.13189069223523 8.92274579108640
C 4.36324703908092 9.23535497226574 9.80385106476944
H 4.43078742587135 9.95017603091101 8.98074704167422
C 4.15735729336013 6.33044938793883 13.01024751710071
C 2.84128460140981 6.28354038963709 13.78374738051385
H 1.98662794053943 6.09674875887284 13.12481071464188
H 2.89654179801374 5.53168585556191 14.57754183109093
O 4.27642314018281 5.04491036241519 12.44767012255655
H 4.96931052778123 6.56350423512871 13.72578659493090
C 5.23752514947387 9.32618562153423 10.88783429966313
H 6.00250310576031 10.10494204372753 10.91473376425674
H 5.85090867807339 8.46481548090353 12.77207116708533

**In TS1 no_Cl**

In 3.17863065810672 6.82395153736683 16.36294221998815
I 0.63342773245156 9.19985014906675 12.45286394052111
O 5.06255381432672 7.69698178869606 16.27371902376154
N 1.38427462890620 7.84386382847101 17.35473302355822
C 2.61545498717780 7.60737406175492 19.43847361729922
N 3.53873522185885 6.67345931825274 18.77876227420483
C 5.36027806501841 8.38024072136778 18.57006143822899
C 5.82626804910520 9.90626680684718 16.72230183005306
C 1.31513862512680 7.79806653289380 18.69752700012285
C 6.19759301532078 10.87108962929349 17.65853230513400
C 5.73618755716898 9.38378803776226 19.47437193078808
C 5.39290239773234 8.63504245023981 17.17922192035661
C 4.96716838465271 7.01845101053536 19.05317778251949
C 6.15992603924876 10.64179093111228 19.04339797349325
C 3.27746919262327 5.26302435831396 19.19846517788620
C 0.92600491630982 4.53855609522731 19.79839437401977
C 1.90688137513875 4.78738365343292 18.83119703426545
C -1.03703857310538 8.28381797656766 18.60686604383498
C 0.28835367571386 8.11124799764880 16.61805319174943

| | | | |
|---|---|---|---|
| C | 1.61607179397729 | 4.60661441902683 | 17.45928278778477 |
| C | 0.10370631483952 | 8.00920357326978 | 19.35663334600054 |
| C | -0.35969463815137 | 4.12588593945850 | 19.44397708243579 |
| C | 5.86787504691147 | 10.17269328103593 | 15.24584481976441 |
| C | -0.94382519996673 | 8.34914169260506 | 17.21543444223316 |
| C | 6.57196728559752 | 11.71336542519146 | 20.01830086671902 |
| O | 2.54785905815907 | 4.83035589585873 | 16.51804629284227 |
| C | 0.31105200753577 | 4.20164625455802 | 17.07880805176039 |
| C | -0.63842263967815 | 3.97249890529575 | 18.07532905701497 |
| C | -0.00788940605936 | 4.06318354505102 | 15.61894604098107 |
| C | -1.41011393085987 | 3.83907392074576 | 20.48469393105629 |
| H | -1.10429084983192 | 4.20990225027136 | 21.47296737385830 |
| H | -1.59851963457962 | 2.75783176004997 | 20.58271349124254 |
| H | -2.37178664162345 | 4.30938759171319 | 20.22883116584822 |
| H | 5.96908737846008 | 12.62657627031810 | 19.89493295367108 |
| H | 7.62521779669897 | 12.00318116658310 | 19.87878090940537 |
| H | 6.45524791122900 | 11.37275548222464 | 21.05625589102815 |
| H | 6.28372962832590 | 11.16659317596008 | 15.03445928766100 |
| H | 4.85923240653313 | 10.11764150074226 | 14.80532359284192 |
| H | 6.46947216865222 | 9.41339360428197 | 14.72442533622660 |
| H | 5.71046637645753 | 9.16254753762611 | 20.54644281423737 |
| H | 6.52708582439003 | 11.84866408079085 | 17.29239142010315 |
| H | 1.17696748148349 | 4.67514307348372 | 20.85556312095137 |
| H | 0.21843765009272 | 4.99493740369912 | 15.07610928937654 |
| H | -1.06489485620003 | 3.80846683599728 | 15.46572052217184 |
| H | 0.61246349544430 | 3.28380325467341 | 15.15023604535798 |
| H | -1.64534070558325 | 3.66795311998971 | 17.77212375417187 |
| H | 5.56280155835198 | 6.24854920618679 | 18.53839224346493 |
| H | 5.16091691959758 | 6.92448531583102 | 20.13845730490035 |
| H | 3.12179059027991 | 8.58751394766751 | 19.47594066656883 |
| H | 2.40987754384163 | 7.29626838546808 | 20.47842361832730 |
| H | 3.43873136334594 | 5.18509478857727 | 20.29050632973812 |
| H | 4.03803508902081 | 4.64860376685079 | 18.69327428783727 |
| H | 0.06259990159220 | 7.93925363307654 | 20.44381481327812 |
| H | -1.99499412255786 | 8.43919531469765 | 19.10530230668774 |
| H | -1.81252788016629 | 8.56428120101780 | 16.59446394078571 |
| H | 0.44374053466797 | 8.11675624284743 | 15.53577946288054 |
| C | 5.39526740786154 | 7.02927110276204 | 12.53382848707903 |
| C | 4.20151763163155 | 6.62147506115266 | 13.14192899424496 |
| C | 4.03295314065566 | 5.27098301690133 | 13.47519569474647 |
| H | 3.10826875980865 | 4.94906047328068 | 13.95968357732462 |
| C | 5.04833192218416 | 4.34810446495526 | 13.21225655536922 |
| H | 4.90934019229713 | 3.30092481664749 | 13.48703711235395 |
| C | 6.23553859916658 | 4.76221962934611 | 12.60549405628247 |
| H | 7.03045543305119 | 4.04175453489944 | 12.40563791034551 |
| C | 3.10212273772131 | 7.63649916260525 | 13.44474648233643 |
| C | 2.20660065457767 | 7.71298606254653 | 12.20999382771426 |
| H | 1.67985801549927 | 6.76617495764130 | 12.04654409359442 |
| H | 2.75203565698501 | 8.01484520270176 | 11.30967084939478 |
| O | 2.31705287503192 | 7.29884751165795 | 14.54840022798584 |
| H | 3.59401130187002 | 8.62381894535543 | 13.57265523814934 |
| C | 6.40650272419058 | 6.10737042470632 | 12.26525595441847 |

| | | | |
|---|---|---|---|
| H | 7.33769085486809 | 6.44031026262369 | 11.80317891318578 |
| H | 5.53856431308913 | 8.08506581394268 | 12.28937822147311 |

**InCl – TS1 Cl_open**

| | | | |
|---|---|---|---|
| Cl | 2.46866738124917 | 7.92701312132764 | 14.60831193200887 |
| In | 5.76477916138511 | 3.78434573294910 | 13.22650825393339 |
| Cl | 5.20983380725930 | 3.37128852698741 | 15.57349081398330 |
| O | 7.16300332146082 | 5.38064269457016 | 13.64696050017164 |
| N | 7.64506469907560 | 2.29612066187588 | 13.31402776109869 |
| C | 8.26459755463831 | 3.32401552613381 | 11.19882644161759 |
| N | 6.90882644927056 | 3.86132022188208 | 11.06937590486235 |
| C | 7.77250951569160 | 6.21540525274330 | 11.44275080286698 |
| C | 8.58629593503029 | 7.24016252579522 | 13.50772307034444 |
| C | 8.34776522125541 | 2.17580855369491 | 12.17418411278441 |
| C | 9.28816801732485 | 8.16954688327117 | 12.74796146339494 |
| C | 8.51526169422044 | 7.16521666819798 | 10.72585628026334 |
| C | 7.79806145081522 | 6.22905311715961 | 12.86804287325794 |
| C | 6.88321831838612 | 5.29029234778926 | 10.66053892018830 |
| C | 9.27406201883062 | 8.15837576514390 | 11.34293739725566 |
| C | 6.08002213231533 | 3.07612273165470 | 10.11537341972563 |
| C | 6.67018777732032 | 0.69539630789443 | 9.52836805795257 |
| C | 6.06578571259205 | 1.60657207566028 | 10.40303928164854 |
| C | 9.25527330026205 | 0.07585964863859 | 12.91857245781096 |
| C | 7.72274180018285 | 1.34377812378860 | 14.25417491046018 |
| C | 5.42219759400561 | 1.14992505344427 | 11.58784154365721 |
| C | 9.16676190326227 | 1.06443303347077 | 11.94508063132896 |
| C | 6.68428266038672 | -0.67695290947676 | 9.78166019346811 |
| C | 8.60109645326686 | 7.26485415474511 | 15.00818539030956 |
| C | 8.52758562002920 | 0.21880002618937 | 14.10251318851951 |
| C | 10.03072398152119 | 9.18760640535675 | 10.54405472763194 |
| O | 4.84973089867964 | 1.96581189887487 | 12.44129452277972 |
| C | 5.42209323542847 | -0.25699119903988 | 11.84733037356186 |
| C | 6.04818300439237 | -1.12198925579935 | 10.95507472286969 |
| C | 4.75068679034204 | -0.74205589695825 | 13.09790305926652 |
| C | 7.32992640859378 | -1.65086253846586 | 8.82999732570677 |
| H | 7.93175561554684 | -1.12541001583858 | 8.07408411853284 |
| H | 6.58371108467166 | -2.25879024037362 | 8.29018409698903 |
| H | 7.99418213151585 | -2.35584731720123 | 9.35577834968762 |
| H | 11.09347608480336 | 9.23493002163343 | 10.83385437785595 |
| H | 9.61683420791805 | 10.20105876255583 | 10.68235501223281 |
| H | 9.98810434258147 | 8.96058320799028 | 9.46885561363874 |
| H | 9.22565544609266 | 8.08760351349108 | 15.38503826457976 |
| H | 8.97392157110129 | 6.31217343610887 | 15.41596237942297 |
| H | 7.58152931768454 | 7.37356398243652 | 15.41043524553121 |
| H | 8.47047987585617 | 7.13341693492359 | 9.63154226217387 |
| H | 9.87308058645447 | 8.93523796200757 | 13.27057574371111 |
| H | 7.15394077401227 | 1.07996205357795 | 8.62305514414923 |
| H | 5.14753429154590 | -0.22254243063073 | 13.98362956605657 |
| H | 4.87889323664812 | -1.82674019189399 | 13.22436622602228 |
| H | 3.67416330887946 | -0.50947927400323 | 13.08345137209162 |
| H | 6.04949107932014 | -2.19421978282297 | 11.18353721924454 |

| | | | |
|---|---|---|---|
| H | 5.82880253781696 | 5.59812037197020 | 10.75650341094423 |
| H | 7.15380358622253 | 5.36283003291793 | 9.58712712968598 |
| H | 8.89731426347301 | 4.14604498820919 | 11.57488400387010 |
| H | 8.67403176191370 | 3.00906901431269 | 10.21993612908048 |
| H | 6.45744268571178 | 3.25512652086716 | 9.08758500937535 |
| H | 5.06376780259039 | 3.49652677613597 | 10.18688284593500 |
| H | 9.69207167119117 | 0.97116043284785 | 10.99404423440581 |
| H | 9.87169102350691 | -0.80882156817226 | 12.74890667400124 |
| H | 8.56285026006714 | -0.53692508459303 | 14.88726674191612 |
| H | 7.09440847135633 | 1.51066515231401 | 15.13367935385723 |
| C | 5.15447635038065 | 8.42331245095073 | 12.00744815554438 |
| C | 4.23311687853874 | 7.37390704865273 | 11.93564402112402 |
| C | 3.43288805983315 | 7.25827942653154 | 10.79024058623006 |
| H | 2.74147618261746 | 6.41626672339198 | 10.71518901001450 |
| C | 3.54682102119431 | 8.17480917717250 | 9.74548032684222 |
| H | 2.91833097847420 | 8.07026388476908 | 8.85777884284239 |
| C | 4.47296730349632 | 9.22088468563206 | 9.82707292289770 |
| H | 4.57257137750400 | 9.93175485294523 | 9.00375009897658 |
| C | 4.14845286145326 | 6.32479745668621 | 13.03300622249710 |
| C | 2.81424142494356 | 6.33927889473649 | 13.78467330626854 |
| H | 1.97962290068814 | 6.15371273530048 | 13.09773767579131 |
| H | 2.83680034727833 | 5.57449340548362 | 14.56911494156045 |
| O | 4.25460516209342 | 5.03404037685255 | 12.48160191570481 |
| H | 4.94770799346997 | 6.54860516753467 | 13.76605138968147 |
| C | 5.27872077431013 | 9.34011292475969 | 10.96040769800863 |
| H | 6.02264845392909 | 10.13682607006111 | 11.02512650606324 |
| H | 5.80480031693790 | 8.50129691326907 | 12.88099524972953 |

**In no_Cl – TS1 Cl_open**

| | | | |
|---|---|---|---|
| In | 3.17883679066005 | 6.80608814611765 | 16.37588941712121 |
| Cl | 0.88126813077583 | 8.98452410360571 | 12.45785083487831 |
| O | 5.06429319082212 | 7.67579000794090 | 16.28599352238239 |
| N | 1.38375643122436 | 7.83838140088586 | 17.35845617763758 |
| C | 2.60933272301228 | 7.61466004306508 | 19.44654936245380 |
| N | 3.53150276883441 | 6.67396663049338 | 18.79522773463319 |
| C | 5.35749871447475 | 8.37513997599321 | 18.57821470866099 |
| C | 5.83437216736787 | 9.88547928325868 | 16.72026997805272 |
| C | 1.31177400417934 | 7.80601279560311 | 18.70112757545851 |
| C | 6.20543717986859 | 10.85630877954238 | 17.65026270546499 |
| C | 5.73351066894662 | 9.38450668162312 | 19.47605801903589 |
| C | 5.39469487524429 | 8.61925614992606 | 17.18559034279647 |
| C | 4.96025911364126 | 7.01773074231836 | 19.07003924210304 |
| C | 6.16216476151296 | 10.63790554774346 | 19.03682453929433 |
| C | 3.26627681640367 | 5.26737200023127 | 19.22537658990730 |
| C | 0.90836818171285 | 4.55081286107679 | 19.81175612937498 |
| C | 1.89855327286015 | 4.78895947993696 | 18.85137436348058 |
| C | -1.03647379791399 | 8.30765265044885 | 18.60056433185695 |
| C | 0.29178339680220 | 8.10648676237112 | 16.61612812487333 |
| C | 1.62084615998758 | 4.59394570353435 | 17.47871459515242 |
| C | 0.10034954923100 | 8.03189756316402 | 19.35567063304505 |
| C | -0.37387411811142 | 4.13439583779105 | 19.44918154189730 |

| | | | |
|---|---|---|---|
| C | 5.88331729771705 | 10.13938842289225 | 15.24183967250057 |
| C | -0.94001061210831 | 8.35883073758339 | 17.20866651461146 |
| C | 6.57383525943049 | 11.71591135454082 | 20.00478176370822 |
| O | 2.56201697481603 | 4.80932184425685 | 16.54470557893786 |
| C | 0.31983369484367 | 4.18417289849999 | 17.08986508781311 |
| C | -0.63918158983775 | 3.96562927767713 | 18.07964721030107 |
| C | 0.01531677617514 | 4.03021311128099 | 15.62849326402153 |
| C | -1.43481346165085 | 3.85978640704692 | 20.48253070572452 |
| H | -1.13554348450957 | 4.23526329154757 | 21.47104999472485 |
| H | -1.62990361925757 | 2.78020423271685 | 20.58561994281729 |
| H | -2.39177848792063 | 4.33357288163245 | 20.21551217094323 |
| H | 5.97120529175944 | 12.62844558370250 | 19.87509023122134 |
| H | 7.62719952507016 | 12.00466341218200 | 19.86395457787987 |
| H | 6.45639950231151 | 11.38220204811493 | 21.04490379794441 |
| H | 6.30596248383097 | 11.12899298313338 | 15.02383558505665 |
| H | 4.87614299949385 | 10.08618600056416 | 14.79779303733116 |
| H | 6.48173161293601 | 9.37175625475596 | 14.72907644493279 |
| H | 5.70433911230626 | 9.17142735413704 | 20.54969903382247 |
| H | 6.53980516394945 | 11.82981635343549 | 17.27770550029203 |
| H | 1.14904642392370 | 4.69849169526414 | 20.86983588179929 |
| H | 0.24461091596582 | 4.95731402168670 | 15.07890917134170 |
| H | -1.03944859762787 | 3.77082098460611 | 15.46771024356337 |
| H | 0.64264601870160 | 3.24831174846588 | 15.17352366623689 |
| H | -1.64304583517509 | 3.65734210913125 | 17.77010028313956 |
| H | 5.55483789439737 | 6.24287841597471 | 18.56152067179440 |
| H | 5.15194993787091 | 6.93055111129070 | 20.15626489305520 |
| H | 3.11851130530580 | 8.59357031385927 | 19.47945851857516 |
| H | 2.40013210785223 | 7.31091581470275 | 20.48795945991893 |
| H | 3.41934365776794 | 5.19865823493480 | 20.31923006627132 |
| H | 4.03012940949311 | 4.64835490469002 | 18.73095202805343 |
| H | 0.05616078619889 | 7.97272385168626 | 20.44338934969660 |
| H | -1.99442573038704 | 8.47495934732986 | 19.09515318029273 |
| H | -1.80660355163378 | 8.57417136482116 | 16.58485609663222 |
| H | 0.45344718410703 | 8.09678215182708 | 15.53478641640887 |
| C | 5.36176991500578 | 7.06908502896083 | 12.50320262392636 |
| C | 4.18753462495193 | 6.63804772425634 | 13.13284535279929 |
| C | 4.05194342159770 | 5.28398021208265 | 13.46574608497611 |
| H | 3.14245734377615 | 4.94376343399175 | 13.96640553564918 |
| C | 5.07866395599320 | 4.38030883085518 | 13.17970787363810 |
| H | 4.96418947192128 | 3.32980005664492 | 13.45316915067334 |
| C | 6.24566397146254 | 4.81796090476081 | 12.55111784828144 |
| H | 7.04987932614372 | 4.11311599905824 | 12.33345008156696 |
| C | 3.07499033450827 | 7.62784640246874 | 13.45935914989920 |
| C | 2.15435699066550 | 7.71923478536320 | 12.23973647288323 |
| H | 1.63071659621411 | 6.76618215706407 | 12.09362480484595 |
| H | 2.71178880113611 | 7.98707114847888 | 11.33417435837572 |
| O | 2.30496242851795 | 7.26281952100106 | 14.56544074999378 |
| H | 3.54623664625753 | 8.62462225377023 | 13.59412964253202 |
| C | 6.38457043785173 | 6.16707060942790 | 12.21179343334097 |
| H | 7.30016660213559 | 6.51859688913671 | 11.73256550985458 |
| H | 5.48086638780794 | 8.12799146733506 | 12.25886777483685 |

**InCl – TS2**

| | | | |
|---|---|---|---|
| In | 4.14983661919420 | 4.88436072561445 | 16.21632585048943 |
| I | 3.32113533081181 | 0.26630764246976 | 14.18170056371773 |
| O | 5.78522496577481 | 6.00821536185798 | 15.62619535147414 |
| N | 2.11081973816359 | 5.87853521001420 | 16.21770964435157 |
| C | 3.00739216506897 | 7.20748444477278 | 18.05305626412563 |
| N | 4.13273697640684 | 6.27121774847441 | 18.20111236544249 |
| C | 5.71770687158381 | 7.88720653430063 | 17.13183044567114 |
| C | 6.17652796090014 | 8.18348954363247 | 14.75144704291964 |
| C | 1.83455692190064 | 6.61309129083126 | 17.31586711233435 |
| C | 6.30922730527291 | 9.55167648825209 | 14.98618503751775 |
| C | 5.85159587184834 | 9.27074416479436 | 17.31805113652605 |
| C | 5.87174702080297 | 7.32856896824523 | 15.84273316648889 |
| C | 5.44842471497572 | 6.97868095023197 | 18.28992845020928 |
| C | 6.14873698206754 | 10.12738310309148 | 16.25760841784922 |
| C | 3.95960066295518 | 5.39639316869272 | 19.39778627114103 |
| C | 1.66228817981815 | 4.93568386991396 | 20.33867809052451 |
| C | 2.65951066423008 | 4.65493720201661 | 19.39668483882160 |
| C | -0.52320952188783 | 6.36820663033414 | 16.90732595501116 |
| C | 1.11252424701749 | 5.38879528946193 | 15.45826209207537 |
| C | 2.44695923389865 | 3.66744210114959 | 18.40601727882520 |
| C | 0.51309628853332 | 6.87315766607184 | 17.68505925542771 |
| C | 0.43384383048137 | 4.27335994544922 | 20.32164492952486 |
| C | 6.34499615731770 | 7.58787451490033 | 13.38456541311894 |
| C | -0.22092652074703 | 5.62126395026443 | 15.76636711235056 |
| C | 6.28551053490004 | 11.61379822947113 | 16.45865379390765 |
| O | 3.38663615275460 | 3.37319798739288 | 17.50162177473452 |
| C | 1.20063837551638 | 2.98697368740561 | 18.37027893171055 |
| C | 0.23158203828619 | 3.30765758628000 | 19.32093588896195 |
| C | 0.96434527424122 | 1.95834720627004 | 17.30485925111685 |
| C | -0.62832327762277 | 4.55728956644443 | 21.35101134454521 |
| H | -0.65871527743592 | 3.77378815617122 | 22.12544027835581 |
| H | -1.63000259749923 | 4.59794188355118 | 20.89778138699999 |
| H | -0.44672055863035 | 5.51381811391056 | 21.86075558709079 |
| H | 5.47466755245802 | 12.16659755398039 | 15.95760361579027 |
| H | 7.23326728992388 | 11.99178919767546 | 16.04603323779433 |
| H | 6.25595423134493 | 11.87544211253486 | 17.52539638010819 |
| H | 6.63708577061845 | 8.35339710619553 | 12.65374287307140 |
| H | 5.41209080062024 | 7.11275408878098 | 13.04437176371695 |
| H | 7.10551800422969 | 6.79287914725308 | 13.39060050194045 |
| H | 5.73239473722431 | 9.68149350876298 | 18.32601500783509 |
| H | 6.54426893020984 | 10.20151576197272 | 14.13723984793169 |
| H | 1.85410472299559 | 5.69837741357668 | 21.10050431333281 |
| H | 1.18411648795380 | 2.35979793023434 | 16.30386741723726 |
| H | -0.07161615085859 | 1.59518553474294 | 17.32502888170385 |
| H | 1.63580274629302 | 1.09401474727717 | 17.42486452411231 |
| H | -0.72867831949461 | 2.78361536225885 | 19.27954181722745 |
| H | 6.21068393690993 | 6.18411599916333 | 18.33900896843589 |
| H | 5.48622069955319 | 7.54560701283125 | 19.23921006201366 |
| H | 3.38086335724029 | 8.06359078092459 | 17.46481023736607 |
| H | 2.67479280109628 | 7.59057567789229 | 19.03400924702801 |

| | | | |
|---|---|---|---|
| H | 4.04277984385934 | 6.02429001703884 | 20.30511519103854 |
| H | 4.80804559254126 | 4.69413395868454 | 19.39066846608672 |
| H | 0.31337740420570 | 7.44390989682613 | 18.59162265671107 |
| H | -1.56054886504977 | 6.54823278988504 | 17.19222185184671 |
| H | -1.00329823322382 | 5.21084546502561 | 15.12950340233725 |
| H | 1.42158935499159 | 4.80720806273368 | 14.58904307298355 |
| C | 6.55149071946943 | 2.89153453862503 | 17.15721977095954 |
| C | 6.62083808545289 | 2.86210543028589 | 15.75391787021434 |
| C | 7.72953707802820 | 3.43755871117336 | 15.11998021262820 |
| H | 7.77597323412612 | 3.43515241826879 | 14.02904589198182 |
| C | 8.74224544023770 | 4.04013976519233 | 15.86467169821307 |
| H | 9.59182009599681 | 4.49664485412420 | 15.35443046432252 |
| C | 8.66286949175151 | 4.07518826281498 | 17.25874247428811 |
| H | 9.45395901484271 | 4.55038482449491 | 17.84098818588531 |
| C | 5.46983349160962 | 2.34219982926001 | 14.90583515279361 |
| C | 4.85520701120326 | 1.07377577950610 | 15.48033894659486 |
| H | 4.33827364732456 | 1.26671559929060 | 16.42622255136438 |
| H | 5.59699723605907 | 0.27602153780034 | 15.59259123920620 |
| O | 4.47331412778912 | 3.35161863567752 | 14.77180408090817 |
| H | 5.84701249094931 | 2.14513294440518 | 13.88663058282593 |
| C | 7.56755727852830 | 3.49682665740457 | 17.90259147253245 |
| H | 7.50581997709945 | 3.50558152375932 | 18.99295673288195 |
| H | 5.69494744807121 | 2.45353056338758 | 17.67232262224302 |
| O | 3.56776465705774 | 5.18476762090868 | 13.57892151708724 |
| C | 4.34207820509926 | 4.31687894180310 | 13.22739420178948 |
| O | 5.02151024275993 | 3.82257950382907 | 12.38368767026131 |

**InCl – TS2 Cl_open**

| | | | |
|---|---|---|---|
| In | 4.28035798156314 | 5.21725262587189 | 16.70230300624458 |
| Cl | 2.53774693662323 | 1.59389449147214 | 14.15431036416352 |
| O | 6.16945288041534 | 6.07837941049851 | 16.68329064121170 |
| N | 2.05381780754079 | 5.68315009731988 | 16.47823595709268 |
| C | 2.56025374496914 | 7.50914932442355 | 18.00517033372766 |
| N | 3.76797211061045 | 6.78372595448243 | 18.42144840055522 |
| C | 5.50022875713536 | 8.28703478209473 | 17.36793695685251 |
| C | 6.86727391308898 | 7.95682156703017 | 15.37354141672928 |
| C | 1.55925273624978 | 6.63453382600962 | 17.28649284779567 |
| C | 6.84331144170892 | 9.33874527195384 | 15.17998903048512 |
| C | 5.48979361270016 | 9.66690199924571 | 17.12618625595321 |
| C | 6.16523295371592 | 7.41375629962449 | 16.47604974644290 |
| C | 4.92528007355722 | 7.71249326502933 | 18.62350342868821 |
| C | 6.15053223925333 | 10.21830436293524 | 16.02704981867745 |
| C | 3.55977645314518 | 6.00530517173371 | 19.68181098593145 |
| C | 1.33066958823947 | 5.08715714602128 | 20.38913075858366 |
| C | 2.49294803943215 | 4.95777828324389 | 19.61818372829907 |
| C | -0.67818125828623 | 6.02576470312263 | 16.65488505822836 |
| C | 1.23495098191880 | 4.89490501036058 | 15.76209407364304 |
| C | 2.69637451173830 | 3.80698635862912 | 18.81799745734113 |
| C | 0.18191380412932 | 6.83142966832872 | 17.39573111664085 |
| C | 0.34438002236499 | 4.10173012938464 | 20.40430386650904 |
| C | 7.60081691363118 | 7.03818484689692 | 14.44081722392418 |

| | | | |
|---|---|---|---|
| C | -0.14568492719452 | 5.04134986966892 | 15.82029093107756 |
| C | 6.13031410916239 | 11.69979041716493 | 15.75645354675228 |
| O | 3.79205468898631 | 3.65914721237381 | 18.05301038037278 |
| C | 1.70958876074495 | 2.78602887980009 | 18.83750715027612 |
| C | 0.56937651026799 | 2.95748831244065 | 19.62306444701955 |
| C | 1.90041174519220 | 1.55292324360181 | 18.00442448621486 |
| C | -0.90444982151876 | 4.24351922548551 | 21.23392585961305 |
| H | -0.97541988934229 | 3.45347099446483 | 21.99778820017986 |
| H | -1.81241913170160 | 4.17188956587821 | 20.61466464988477 |
| H | -0.92856661950849 | 5.21121207019945 | 21.75367723021295 |
| H | 5.57835644841767 | 11.93319593986760 | 14.83200689707116 |
| H | 7.14727297533417 | 12.10251025549363 | 15.63350792737585 |
| H | 5.64912308988172 | 12.24804933584357 | 16.57819110912723 |
| H | 8.28009434000430 | 7.60222535798033 | 13.78763345140426 |
| H | 6.89973768791841 | 6.48154150149711 | 13.79934761418393 |
| H | 8.17387929374007 | 6.28354627006628 | 14.99714168054390 |
| H | 4.97495649112705 | 10.32549932398498 | 17.83360341714150 |
| H | 7.38242545286431 | 9.74888865757072 | 14.32009214693114 |
| H | 1.20357944563523 | 5.98625441431620 | 21.00101103922693 |
| H | 1.83132765761483 | 1.77850762566759 | 16.92816829601041 |
| H | 1.13754717473969 | 0.79867554111345 | 18.23840984245354 |
| H | 2.89368881904198 | 1.11073375769979 | 18.17205357684907 |
| H | -0.18218132496687 | 2.16148645572329 | 19.62153468323788 |
| H | 5.69562132436513 | 7.10687696272083 | 19.12456638450539 |
| H | 4.62025305152883 | 8.51474908317276 | 19.32085273340801 |
| H | 2.88887973384212 | 8.30084601023017 | 17.30914053953121 |
| H | 2.07434406664292 | 7.99958012111140 | 18.86857025401731 |
| H | 3.34078809509992 | 6.71973423730473 | 20.49759736344645 |
| H | 4.53060552928765 | 5.53269060966301 | 19.89799646873843 |
| H | -0.20205430833631 | 7.59554050806005 | 18.07216004681614 |
| H | -1.75802770890950 | 6.15721524497674 | 16.73621907952511 |
| H | -0.78538904595984 | 4.38579004124278 | 15.23088650813583 |
| H | 1.71869754171245 | 4.13245646931336 | 15.14683505851776 |
| C | 6.53616345692576 | 2.68941720710263 | 16.51683503361145 |
| C | 6.34073597707217 | 2.86334439059798 | 15.13787651397362 |
| C | 7.45053367358424 | 3.09622089961292 | 14.31724467781213 |
| H | 7.29845595409802 | 3.24927670132131 | 13.24669763350530 |
| C | 8.73410584035799 | 3.15946536335939 | 14.86077046294161 |
| H | 9.58967572317540 | 3.35077739163952 | 14.21086932742848 |
| C | 8.92091301089736 | 2.99221078199956 | 16.23445590037785 |
| H | 9.92327970985520 | 3.04953555881220 | 16.66176458940076 |
| C | 4.94835141601432 | 2.87846007385621 | 14.53652362032163 |
| C | 4.15089866469989 | 1.65329557158157 | 14.96266213435141 |
| H | 3.95595683887346 | 1.65882673525403 | 16.04122060002448 |
| H | 4.67890628560014 | 0.73399788171585 | 14.68330254281280 |
| O | 4.24363616260730 | 4.06750211570568 | 14.88011020372471 |
| H | 5.03467260635803 | 2.88911934822006 | 13.43644304129572 |
| C | 7.81953246731542 | 2.75848365704713 | 17.06005642679563 |
| H | 7.95775911011298 | 2.63206526262133 | 18.13505901757366 |
| H | 5.68392084531862 | 2.51707704553384 | 17.17988736963859 |
| O | 4.04988601713918 | 6.38656803818057 | 14.43974545105377 |
| C | 4.35601402671956 | 5.44342183001354 | 13.73095154054053 |

| | | | |
|---|---|---|---|
| O | 4.67554971271586 | 5.05473203501297 | 12.64852543889273 |

**InCl – Int2**

| | | | |
|---|---|---|---|
| In | 3.90665126772848 | 5.06100505321378 | 16.65360197983822 |
| I | 4.62375275001654 | 0.16577881646318 | 17.35329527955109 |
| O | 5.74398594486308 | 6.00806186839338 | 16.57327529961739 |
| N | 1.91551390824539 | 6.14701410202718 | 16.28189836756233 |
| C | 2.44082744473693 | 7.43702444500525 | 18.27822660394247 |
| N | 3.51739205088774 | 6.49477045210952 | 18.61559425782266 |
| C | 5.32122121230815 | 8.02392735624398 | 17.81890191232390 |
| C | 6.28430552052518 | 8.10778441336323 | 15.57489888649029 |
| C | 1.43270589226150 | 6.87215705073893 | 17.31001639001318 |
| C | 6.34473354445300 | 9.49724443656246 | 15.69535439354302 |
| C | 5.39705419644962 | 9.42131197078717 | 17.89332450107361 |
| C | 5.77001082198030 | 7.35526116810071 | 16.65920572060298 |
| C | 4.78485068222387 | 7.20902816337719 | 18.95520443808712 |
| C | 5.91299111696937 | 10.18242326319271 | 16.84232102296652 |
| C | 3.12597649676017 | 5.60312288735187 | 19.74827273334509 |
| C | 0.69312088833479 | 5.06026775227746 | 20.16504131950580 |
| C | 1.88298133500673 | 4.81776449218609 | 19.46730160682081 |
| C | -0.79832453502886 | 6.66803350876598 | 16.43650617580128 |
| C | 1.08285531358472 | 5.68123732787658 | 15.33002468885035 |
| C | 1.92554467945172 | 3.82772313426021 | 18.45905434075566 |
| C | 0.06789854060166 | 7.14581697361771 | 17.41462977915220 |
| C | -0.47821787877962 | 4.35798711887717 | 19.87767894680477 |
| C | 6.73352774391580 | 7.39405782152635 | 14.33302887715265 |
| C | -0.28258663961799 | 5.93301363078235 | 15.36716591584145 |
| C | 6.01656730262033 | 11.68221644742053 | 16.93288259922248 |
| O | 3.05814392283789 | 3.55136271618814 | 17.79310497253455 |
| C | 0.74090160491187 | 3.11485492962821 | 18.14103003227615 |
| C | -0.42422987930926 | 3.39630676786427 | 18.85456449164048 |
| C | 0.77960381568503 | 2.10166929961886 | 17.03587104345729 |
| C | -1.75095419615597 | 4.60048623130595 | 20.64548380746503 |
| H | -1.69650083364432 | 5.53174143940938 | 21.22639312683549 |
| H | -1.95636942063453 | 3.78233114467310 | 21.35441218360658 |
| H | -2.62087022986655 | 4.66850961083868 | 19.97491205999337 |
| H | 5.58252124291249 | 12.17111553630070 | 16.04761848578459 |
| H | 7.06627835501231 | 12.00990920586118 | 16.99853742228883 |
| H | 5.49437722369857 | 12.06526810369279 | 17.82032432317428 |
| H | 7.03504164874094 | 8.10806783347617 | 13.55561400838909 |
| H | 5.93415629695741 | 6.75100384065783 | 13.93367141195935 |
| H | 7.58103018146637 | 6.72305271722262 | 14.53844939334549 |
| H | 5.05191478545415 | 9.92169265947425 | 18.80384721718213 |
| H | 6.73886168287866 | 10.07324677451570 | 14.85223086072811 |
| H | 0.68642340136888 | 5.82262970480235 | 20.95097468374602 |
| H | 1.06076218934476 | 2.57049004561781 | 16.07867212300498 |
| H | -0.19396660457272 | 1.60932486625508 | 16.91320475713655 |
| H | 1.54976370284403 | 1.33963222534745 | 17.22492512331169 |
| H | -1.33420336908996 | 2.84359430959751 | 18.59996774743152 |
| H | 5.50637451729521 | 6.42574417774053 | 19.23409107845298 |
| H | 4.61660223025565 | 7.84526756325512 | 19.84462885322623 |

| | | | |
|---|---|---|---|
| H | 2.91585853902969 | 8.30913303109562 | 17.79695625417732 |
| H | 1.92313945997378 | 7.79322382062299 | 19.18685472546534 |
| H | 2.99427438295158 | 6.22234163784606 | 20.65590004932943 |
| H | 3.97766325192589 | 4.92620881398989 | 19.91683043851587 |
| H | -0.30200066408607 | 7.70681731975930 | 18.27275829441340 |
| H | -1.86967342219934 | 6.85856773459550 | 16.51214353902071 |
| H | -0.92691508825860 | 5.54121525179626 | 14.58123372658857 |
| H | 1.55384792281337 | 5.08664537312742 | 14.54552033978878 |
| C | 8.44548275958897 | 1.85548652974952 | 14.31110359260906 |
| C | 7.59782683391241 | 2.62243068890252 | 15.11959409509349 |
| C | 8.03897568319695 | 3.86222389721243 | 15.59750303509277 |
| H | 7.37042018331496 | 4.48771317529402 | 16.19165057002144 |
| C | 9.31572119485253 | 4.32211987145582 | 15.26911404368798 |
| H | 9.64950461358615 | 5.29347040328680 | 15.63951894128394 |
| C | 10.16193154639338 | 3.55060656897960 | 14.46949479759207 |
| H | 11.15807216205472 | 3.91512381119804 | 14.21305152062244 |
| C | 6.24055904102259 | 2.06459781242249 | 15.50363011432150 |
| C | 6.34548882242210 | 1.39040060256411 | 16.86016068790774 |
| H | 6.43948625760501 | 2.12418147621111 | 17.66875834546282 |
| H | 7.19708903910041 | 0.70414821525054 | 16.88160858847687 |
| O | 5.24010520431129 | 3.09659533952964 | 15.59354196302284 |
| H | 5.91159578219389 | 1.35304635414740 | 14.73203130433983 |
| C | 9.72230001862272 | 2.31520820500368 | 13.98772255207823 |
| H | 10.37062316340489 | 1.71356348938107 | 13.34854057270381 |
| H | 8.09354889735313 | 0.89824037814996 | 13.91913858713215 |
| O | 3.73021068649720 | 4.39138280433099 | 14.62656744161198 |
| C | 4.53587012231410 | 3.39568806260886 | 14.39858016388958 |
| O | 4.70982067381245 | 2.76887902322329 | 13.37504915069077 |

**IntCl – Int2 Cl_open**

| | | | |
|---|---|---|---|
| In | 3.94005698809870 | 5.19392701989045 | 16.39168600507156 |
| Cl | 3.99148563602784 | 0.40750458103346 | 15.57985057981893 |
| O | 5.71979101831967 | 6.23375135039115 | 16.19133128044047 |
| N | 1.87973138775687 | 6.20346463062070 | 16.27099886596355 |
| C | 2.54686488211288 | 7.40974741687565 | 18.27630231903733 |
| N | 3.68923347351257 | 6.50143707021132 | 18.45563582937815 |
| C | 5.34442048123508 | 8.15713713403849 | 17.58656396491454 |
| C | 6.11816097518504 | 8.40149809793533 | 15.28182365606966 |
| C | 1.47124170433443 | 6.85208148810272 | 17.37924371555813 |
| C | 6.14015698096868 | 9.78320659046437 | 15.47649820433604 |
| C | 5.37688228478567 | 9.55052603060327 | 17.73506899676132 |
| C | 5.71310400431275 | 7.57194597276385 | 16.35619346547472 |
| C | 4.95138147780499 | 7.25761143745246 | 18.71684403897786 |
| C | 5.77561111167506 | 10.38742147797967 | 16.69089531187678 |
| C | 3.44904554774099 | 5.53369153579771 | 19.56732933247184 |
| C | 1.09210703807724 | 4.88946673488689 | 20.19826026496998 |
| C | 2.20815329373589 | 4.72098662566696 | 19.36897998624710 |
| C | -0.82340247810277 | 6.59732795699992 | 16.70805294356525 |
| C | 0.97909577258855 | 5.75272919448068 | 15.37630827754922 |
| C | 2.17008811698549 | 3.79066395006450 | 18.30514705397898 |
| C | 0.11331566708109 | 7.06063921865763 | 17.62649500668437 |

| | | | |
|---|---|---|---|
| C | -0.08638021731957 | 4.17144962747822 | 19.99244455185092 |
| C | 6.50285742410944 | 7.76846543452451 | 13.97639052769987 |
| C | -0.38521867346297 | 5.94255416290440 | 15.55222282839108 |
| C | 5.82689871349512 | 11.88361899787935 | 16.85625648232554 |
| O | 3.23618032896283 | 3.58547747111555 | 17.51152068009492 |
| C | 0.97347189821200 | 3.06539172607909 | 18.06792459418428 |
| C | -0.11709511749056 | 3.27302227392314 | 18.91305525510906 |
| C | 0.91195832849372 | 2.11713355617675 | 16.90865319960738 |
| C | -1.27749934407005 | 4.33359945303898 | 20.89958085499453 |
| H | -1.17746474178581 | 5.22367842316835 | 21.53620512472928 |
| H | -1.39749671388264 | 3.46429985449593 | 21.56588106636521 |
| H | -2.21234298504953 | 4.42842005826546 | 20.32672362687187 |
| H | 5.26169180812955 | 12.39724073313751 | 16.06358057490504 |
| H | 6.86120298566979 | 12.25927245318210 | 16.80769993780869 |
| H | 5.40700102809953 | 12.19232417872425 | 17.82336968661302 |
| H | 6.74866478333855 | 8.53009812073033 | 13.22504162598176 |
| H | 5.69039531711538 | 7.13506278629970 | 13.58813322297467 |
| H | 7.37123489296543 | 7.10310973948812 | 14.09832759342515 |
| H | 5.09397399777748 | 9.98652746254806 | 18.69863633905502 |
| H | 6.44923604396130 | 10.41925353910169 | 14.64111024286957 |
| H | 1.15059321496243 | 5.60668793489214 | 21.02346957683808 |
| H | 1.12880781641420 | 2.63498401679343 | 15.96052748248776 |
| H | -0.07685595450492 | 1.64575770012235 | 16.83485095273895 |
| H | 1.67886212966233 | 1.33365630446434 | 16.99111023638607 |
| H | -1.03599411916377 | 2.71070880459038 | 18.71894883967676 |
| H | 5.72870945113474 | 6.49567974221180 | 18.88278266699317 |
| H | 4.83985503239702 | 7.83545052768556 | 19.65381111743383 |
| H | 2.93822609129336 | 8.32510599407215 | 17.79979018832054 |
| H | 2.10892131933726 | 7.69615655506888 | 19.24912544246210 |
| H | 3.39332807673388 | 6.09538892185505 | 20.51906896509690 |
| H | 4.33592176298063 | 4.88251000303583 | 19.60751024942555 |
| H | -0.19445831569768 | 7.55909069105880 | 18.54557506677132 |
| H | -1.88910039196977 | 6.73712065489136 | 16.89399082478175 |
| H | -1.08669274036107 | 5.56534406418986 | 14.81227562069520 |
| H | 1.39555564190619 | 5.22382955975081 | 14.51730185266108 |
| C | 8.44078912670999 | 1.55405223118780 | 15.17406942547048 |
| C | 7.47670481311151 | 2.55529682228833 | 15.36586450925529 |
| C | 7.88842910108560 | 3.82428535350581 | 15.78440680315896 |
| H | 7.16000936312448 | 4.62609052710882 | 15.90800574172492 |
| C | 9.24314447854392 | 4.07997229240583 | 16.01701855600853 |
| H | 9.55019548678945 | 5.07688279279581 | 16.33912109571667 |
| C | 10.19694222126908 | 3.07846129620360 | 15.83534980497618 |
| H | 11.25339807414215 | 3.28359939087238 | 16.01596338860080 |
| C | 6.02036125664224 | 2.21890766610395 | 15.10366367350259 |
| C | 5.50442427697824 | 1.21318168799507 | 16.12923416620875 |
| H | 5.27128334432052 | 1.71824929432052 | 17.07534867328724 |
| H | 6.24142967027969 | 0.42114403091245 | 16.29925202313477 |
| O | 5.18759991080302 | 3.38704997585993 | 15.16409649897960 |
| H | 5.90925299175693 | 1.81047851556287 | 14.08563904982027 |
| C | 9.79069027255509 | 1.81148102828250 | 15.40753251952549 |
| H | 10.52826668695070 | 1.02325694991092 | 15.24742347830643 |
| H | 8.13403269927253 | 0.56574479106351 | 14.82203465146952 |

| | | | |
|---|---|---|---|
| O | 3.64394331504332 | 4.71538812323506 | 14.31282975025704 |
| C | 4.43763910905275 | 3.73759435897427 | 13.99900493791565 |
| O | 4.58981860153756 | 3.16524087914817 | 12.94252773350627 |

**InCl – TS3**

| | | | |
|---|---|---|---|
| In | 3.28207347086869 | 4.89873212102224 | 10.65898614966904 |
| I | 4.58576968527674 | -0.54455341365165 | 15.35789509110938 |
| O | 2.70047799854299 | 5.80217626032863 | 12.40734589552998 |
| N | 4.21294798589740 | 6.10118306546701 | 8.95453985185775 |
| C | 1.96569678693086 | 7.00340863328329 | 8.68539765087388 |
| N | 1.43269474452719 | 5.96701812174585 | 9.58502687085685 |
| C | 1.26504261228060 | 7.53710373841692 | 11.52335225009902 |
| C | 2.96044795530492 | 8.02631851909093 | 13.21315346851443 |
| C | 3.31724809841981 | 6.65797310742014 | 8.11290102712764 |
| C | 2.53724803838160 | 9.35560380269139 | 13.20045564969383 |
| C | 0.87904736642292 | 8.88544869928501 | 11.54018145240379 |
| C | 2.31440899444918 | 7.09736220730833 | 12.36069721700563 |
| C | 0.55512883205164 | 6.54811529523804 | 10.64962199535080 |
| C | 1.49954626633573 | 9.81640930196165 | 12.37435827289092 |
| C | 0.69347399806988 | 4.90930973530202 | 8.82853287818128 |
| C | 1.26454915328651 | 4.42718451318182 | 6.41748026491003 |
| C | 1.53761969685806 | 4.25132957264409 | 7.78046994940849 |
| C | 4.95919389684404 | 6.69273175981242 | 6.35617613176467 |
| C | 5.46529221350317 | 5.83513904016358 | 8.53372808092166 |
| C | 2.63071414859777 | 3.45152725885838 | 8.18590857160412 |
| C | 3.66767715414287 | 6.96259145510994 | 6.79640386163553 |
| C | 2.06008516931332 | 3.84429143007975 | 5.43080489144745 |
| C | 4.07287466233938 | 7.55217299564627 | 14.10212014872270 |
| C | 5.88061252456718 | 6.13012010218936 | 7.24224536793739 |
| C | 1.07509495511331 | 11.26094365041799 | 12.39813786739606 |
| O | 2.90946497061376 | 3.23695260354241 | 9.48428201090990 |
| C | 3.45558548138657 | 2.85562925187093 | 7.19751643685783 |
| C | 3.15142926574971 | 3.06591401030141 | 5.85285702204936 |
| C | 4.62753602383124 | 2.02799724299507 | 7.63380401112826 |
| C | 1.77048488532273 | 4.04014968537627 | 3.96593376897341 |
| H | 0.83182596791018 | 4.59047926910163 | 3.81538071051875 |
| H | 1.68273656775748 | 3.07752562850521 | 3.44008199233931 |
| H | 2.57373526784374 | 4.60495693534208 | 3.46600495087249 |
| H | 1.90695909050225 | 11.93067389410794 | 12.13019249894356 |
| H | 0.73096451006861 | 11.56192183401866 | 13.39956913007657 |
| H | 0.25370833034744 | 11.44692197511108 | 11.69300200910968 |
| H | 4.49289655487682 | 8.38181398474725 | 14.68512114598020 |
| H | 4.88053176355200 | 7.08826583716050 | 13.51489949619425 |
| H | 3.71981864026721 | 6.77601440407658 | 14.79740764949991 |
| H | 0.05798214349561 | 9.20639439900752 | 10.89130080141476 |
| H | 3.04246895050791 | 10.06577809034255 | 13.86226492483987 |
| H | 0.40324606631446 | 5.03669581292073 | 6.12573633282525 |
| H | 5.31099506810862 | 2.61449667394105 | 8.26913574563369 |
| H | 5.18838420715579 | 1.64729909377569 | 6.77024216562193 |
| H | 4.30162878575801 | 1.17904057631991 | 8.25286692816937 |
| H | 3.79378978697457 | 2.60202825315465 | 5.09776363068194 |

| | | | |
|---|---|---|---|
| H | 0.20052189776378 | 5.69328744045831 | 11.24645656739542 |
| H | -0.32714370096562 | 7.01543037329594 | 10.17504388036585 |
| H | 2.07745573711290 | 7.92207837232482 | 9.28668237404526 |
| H | 1.25876315461318 | 7.21755321763530 | 7.86538382484802 |
| H | -0.20308697682186 | 5.36665173544652 | 8.37097274648152 |
| H | 0.35818462549814 | 4.17211635444777 | 9.57444474268964 |
| H | 2.91798382920247 | 7.38181314901011 | 6.12563874364398 |
| H | 5.24368943135489 | 6.90921450856347 | 5.32574554622080 |
| H | 6.90072300620551 | 5.90309596787808 | 6.93564676120425 |
| H | 6.12123555424477 | 5.36127297816103 | 9.26518963688522 |
| C | 2.29191596241088 | 0.11140711470679 | 12.19132084294332 |
| C | 3.61382484818508 | 0.56414481937696 | 12.12025960952027 |
| C | 4.44420507184679 | 0.09137728230736 | 11.09605522371260 |
| H | 5.47699628388442 | 0.43791272672012 | 11.02257751918184 |
| C | 3.96156890518245 | -0.82914806749857 | 10.16682577011587 |
| H | 4.62120010015559 | -1.20300919724918 | 9.38175317231436 |
| C | 2.64150639808592 | -1.27838491766986 | 10.24500359271016 |
| H | 2.26671552143615 | -2.00395249652296 | 9.52134165637811 |
| C | 4.09223607130394 | 1.59744892225329 | 13.09347511467170 |
| C | 5.53288417249794 | 1.51031099664566 | 13.55310466294134 |
| H | 5.77961837673860 | 1.93315124157722 | 14.52282985309199 |
| H | 6.13305019702793 | 0.66091733356671 | 13.24552439074357 |
| O | 4.01979773361047 | 2.96205760129678 | 12.35943316588446 |
| H | 3.40035724964851 | 1.73406858557014 | 13.92638851059740 |
| C | 1.80703317410928 | -0.80490575007948 | 11.26029815069669 |
| H | 0.77919858872877 | -1.16288557716223 | 11.33570562225976 |
| H | 1.65397535651059 | 0.45837046456769 | 13.00618257477259 |
| O | 5.34558097188382 | 4.39026476374079 | 11.28591423621615 |
| C | 5.24772050608900 | 3.37588579173869 | 12.04315815161967 |
| O | 6.23440655978456 | 2.72084403315910 | 12.52049086949313 |

**IntCl – TS3 no_Cl**

| | | | |
|---|---|---|---|
| In | 3.27300978065834 | 4.87571669401974 | 10.63460799228908 |
| Cl | 4.56719632041519 | -0.20450860537197 | 15.09152361583691 |
| O | 2.67944219548855 | 5.76110757910040 | 12.38866135680239 |
| N | 4.21355750777211 | 6.09268447737855 | 8.94189839623781 |
| C | 1.97299838708287 | 7.01132358239276 | 8.67696885129831 |
| N | 1.43219503721212 | 5.96734444809318 | 9.56264765151622 |
| C | 1.26213723880071 | 7.51612976762700 | 11.51749537711780 |
| C | 2.95242482694371 | 7.97403429691646 | 13.22118040320933 |
| C | 3.32477164941656 | 6.66670743738623 | 8.10492824946801 |
| C | 2.53906299124700 | 9.30647741301427 | 13.22108546959754 |
| C | 0.88579907691054 | 8.86690234574009 | 11.54730364407904 |
| C | 2.30381614777592 | 7.05936721849093 | 12.35534157510394 |
| C | 0.55167635922402 | 6.54114623493552 | 10.62861546379603 |
| C | 1.50863218124995 | 9.78385735018717 | 12.39528327249114 |
| C | 0.69138498907237 | 4.92236534509418 | 8.79050888091566 |
| C | 1.27478393196971 | 4.45903151701353 | 6.37842931424298 |
| C | 1.53715345182730 | 4.26792845610303 | 7.74131132866333 |
| C | 4.97532143550862 | 6.72271704166399 | 6.35688519153338 |
| C | 5.46696510571039 | 5.82878500867240 | 8.52315836249390 |

| | | | |
|---|---|---|---|
| C | 2.62135003729721 | 3.45560887578466 | 8.14603955913268 |
| C | 3.68256746281829 | 6.99019845074156 | 6.79494086284961 |
| C | 2.07136878985646 | 3.87775805597557 | 5.39138031699223 |
| C | 4.05763575263873 | 7.48167727642517 | 14.10926048322889 |
| C | 5.89001609271613 | 6.14287675442410 | 7.23862795162413 |
| C | 1.09078408760558 | 11.22991776835553 | 12.43722491388704 |
| O | 2.89193078356366 | 3.22826784057677 | 9.44378136499459 |
| C | 3.44799850933606 | 2.86261079905384 | 7.15762883153815 |
| C | 3.15476046028096 | 3.08816681731921 | 5.81296822958990 |
| C | 4.61165345943991 | 2.02382081986786 | 7.59528870943562 |
| C | 1.77580555284129 | 4.06685603413493 | 3.92694112746945 |
| H | 1.01035578000513 | 4.83925343294341 | 3.77031852275780 |
| H | 1.40517485774640 | 3.13658436861765 | 3.46814668790075 |
| H | 2.67615562759962 | 4.36240652146826 | 3.36706181580952 |
| H | 1.94063970299493 | 11.90166735049782 | 12.24138809057531 |
| H | 0.68714942163192 | 11.50276589110535 | 13.42467595874703 |
| H | 0.31435995546761 | 11.44266211194264 | 11.68994418522439 |
| H | 4.48080630651018 | 8.30133251015711 | 14.70401798043791 |
| H | 4.86462485457987 | 7.01925510466487 | 13.51991530995928 |
| H | 3.69615619819037 | 6.69966949600941 | 14.79361606111938 |
| H | 0.07091385631398 | 9.20128830747738 | 10.89738113551398 |
| H | 3.04660836678727 | 10.00585675744887 | 13.89256600638616 |
| H | 0.42202789274295 | 5.08053580904432 | 6.08681119704903 |
| H | 5.29982939302093 | 2.60354976136247 | 8.23187631792323 |
| H | 5.17022466452200 | 1.63799512655519 | 6.73254542304361 |
| H | 4.27644702958854 | 1.17814357403541 | 8.21389854814266 |
| H | 3.79989412797370 | 2.62869965181760 | 5.05752675243659 |
| H | 0.18945383160675 | 5.68167617259631 | 11.21400559610637 |
| H | -0.32540632428996 | 7.01844869793266 | 10.15423888162708 |
| H | 2.08734002376503 | 7.92249071920071 | 9.28912509441376 |
| H | 1.26951634699165 | 7.23877206301748 | 7.85745936029051 |
| H | -0.19909770264754 | 5.39030731791783 | 8.33161706075981 |
| H | 0.34618628677490 | 4.17979472533895 | 9.52646745221470 |
| H | 2.93783335974477 | 7.42297251798810 | 6.12726368505932 |
| H | 5.26602227312564 | 6.95499737690893 | 5.33165168964935 |
| H | 6.91106393656490 | 5.91742044818511 | 6.93394544441984 |
| H | 6.11736932791661 | 5.34201467892954 | 9.25092397223121 |
| C | 2.31231147229386 | 0.06579198240602 | 12.29429437149404 |
| C | 3.61270811454360 | 0.56579534806099 | 12.16995802794509 |
| C | 4.42459093555921 | 0.10533945067195 | 11.12590187268317 |
| H | 5.44126857322809 | 0.48775081929225 | 11.01248957875067 |
| C | 3.94526951885638 | -0.84727991604761 | 10.22746041899910 |
| H | 4.59121748347884 | -1.21063758441740 | 9.42608902747636 |
| C | 2.64586894814339 | -1.34226352403722 | 10.35760472765774 |
| H | 2.27304902154458 | -2.09187728687456 | 9.65769576030287 |
| C | 4.08765165039436 | 1.63141780824484 | 13.10716226412477 |
| C | 5.53237901180614 | 1.57888800310589 | 13.55993489251827 |
| H | 5.76128774563872 | 1.99372683850714 | 14.53674916978448 |
| H | 6.12908546125537 | 0.72011215062035 | 13.27182691312998 |
| O | 4.01181845101776 | 2.97116383540575 | 12.30589438599872 |
| H | 3.40090963187671 | 1.80278039522273 | 13.93585634666710 |
| C | 1.83021163421624 | -0.88297662081033 | 11.39467684777767 |

| | | | |
|---|---|---|---|
| H | 0.81924906183205 | -1.27668986752505 | 11.51094695898268 |
| H | 1.69254994180870 | 0.40111331316646 | 13.12748699445120 |
| O | 5.35337734365604 | 4.40286736624301 | 11.26181465567210 |
| C | 5.24005333786065 | 3.39900185602713 | 12.03008806959017 |
| O | 6.21855100688063 | 2.76351823525720 | 12.55589547165928 |

**InCl – FC**

| | | | |
|---|---|---|---|
| In | 3.26513802623059 | 5.08118019879928 | 10.48724933967417 |
| O | 2.69451733817607 | 5.82204975611661 | 12.28772933176341 |
| N | 4.26187338536265 | 6.35003932568531 | 8.91684839713315 |
| C | 1.97864183816182 | 7.16705506762925 | 8.57768805945337 |
| N | 1.46914224088453 | 6.05485520398247 | 9.40041140175642 |
| C | 1.15721025442071 | 7.50211190802660 | 11.42373862911072 |
| C | 2.75520076030288 | 8.02081940577487 | 13.19920578722407 |
| C | 3.39179224531606 | 6.96467401773767 | 8.08639621659935 |
| C | 2.24067384046080 | 9.31809560309586 | 13.23419917033817 |
| C | 0.68017471991375 | 8.82029400398333 | 11.49120249108927 |
| C | 2.20522938422127 | 7.09806463285681 | 12.28065092604737 |
| C | 0.52697439935517 | 6.52689327734192 | 10.47337250870697 |
| C | 1.20342571452050 | 9.74843511712088 | 12.39292159458179 |
| C | 0.83186094854232 | 4.98365834980341 | 8.56661255892484 |
| C | 1.62977339036207 | 4.53422252023313 | 6.21715246047847 |
| C | 1.78367611382907 | 4.34698233308609 | 7.59663463704653 |
| C | 5.15770613850298 | 7.34162181244041 | 6.49536240861577 |
| C | 5.55894540159757 | 6.21825761006700 | 8.57027361512931 |
| C | 2.84015304529041 | 3.54304693748831 | 8.08069656770011 |
| C | 3.81965571569744 | 7.46607284160005 | 6.85683838826536 |
| C | 2.49844721530906 | 3.94733095981822 | 5.29447700157317 |
| C | 3.86546839634130 | 7.58555378706521 | 14.11096892652119 |
| C | 6.04763429651017 | 6.71502511533482 | 7.37064700967911 |
| C | 0.66713345505704 | 11.15164103550839 | 12.47996846777892 |
| O | 3.01973251981043 | 3.33220330942041 | 9.41411966268507 |
| C | 3.73827178567906 | 2.94625761021187 | 7.16639871763158 |
| C | 3.54539648638703 | 3.16117281107835 | 5.79996023762227 |
| C | 4.87758896583191 | 2.11268493974827 | 7.67989283459130 |
| C | 2.30670831475062 | 4.12210503891941 | 3.81209621813873 |
| H | 1.64749084338550 | 4.97152351399179 | 3.58862904271775 |
| H | 1.85114349302530 | 3.22578284193983 | 3.36275791911409 |
| H | 3.26452296111884 | 4.28667138563690 | 3.29806682945635 |
| H | 1.47824140412312 | 11.89400159663262 | 12.45743469441567 |
| H | 0.11476151623246 | 11.30760431926594 | 13.41946916547719 |
| H | -0.01883527912849 | 11.37170291240262 | 11.65145028545207 |
| H | 4.21125824892951 | 8.41916735691330 | 14.73460902951182 |
| H | 4.72196672917716 | 7.19584215512289 | 13.53931667437014 |
| H | 3.53839983839964 | 6.76789279176707 | 14.77035263407249 |
| H | -0.13926421276781 | 9.11718166031858 | 10.82976659948756 |
| H | 2.67224693539221 | 10.02615716787162 | 13.94745759043666 |
| H | 0.79842147103953 | 5.14805843230101 | 5.85737194783541 |
| H | 5.50779474311388 | 2.68376489368943 | 8.38053173209000 |
| H | 5.50983354811066 | 1.75931302456108 | 6.85581449410353 |
| H | 4.51209482966200 | 1.23287240249692 | 8.23167666365067 |

| | | | |
|---|---|---|---|
| H | 4.24458471317305 | 2.69731278571126 | 5.09809729806211 |
| H | 0.20070012135969 | 5.61905584989470 | 11.00429647931920 |
| H | -0.36306971417110 | 6.97417984184370 | 9.99838993290485 |
| H | 1.97409181813337 | 8.06699220029304 | 9.21754215152307 |
| H | 1.31213034189324 | 7.35930792688042 | 7.72090439393895 |
| H | -0.02938458255615 | 5.42170639797636 | 8.03276119362370 |
| H | 0.44590942881197 | 4.23437868303158 | 9.27471209078634 |
| H | 3.09790807668252 | 7.93872238035671 | 6.19066910760134 |
| H | 5.50411380875679 | 7.72410279136896 | 5.53466102946096 |
| H | 7.10214314361580 | 6.59889902148748 | 7.12473401207630 |
| H | 6.19509170632183 | 5.69173399703177 | 9.28210922172038 |
| C | 3.22210700555620 | -0.71337896620927 | 13.26320181068957 |
| C | 3.55392490266277 | 0.26837532846553 | 12.32327182530518 |
| C | 2.89479516844629 | 0.30627085246308 | 11.08585595551283 |
| H | 3.11554945300659 | 1.09906210004959 | 10.36667180491935 |
| C | 1.91329007333612 | -0.64093862211459 | 10.79854758534838 |
| H | 1.39045540945415 | -0.60252102821219 | 9.84193219178072 |
| C | 1.59667989572558 | -1.63411844916560 | 11.73031195191344 |
| H | 0.83161260881662 | -2.37600924367634 | 11.49823742622140 |
| C | 4.65161545267396 | 1.23666417449301 | 12.63577502097191 |
| C | 5.97009529739690 | 1.00065293282092 | 11.87329471720810 |
| H | 6.76627466882713 | 0.57793096744431 | 12.49450966910922 |
| H | 5.82247145429404 | 0.39550818107270 | 10.96900089170642 |
| O | 4.30121912734345 | 2.60552763217152 | 12.19291300151913 |
| H | 4.81732848219786 | 1.30712276893765 | 13.72020354046714 |
| C | 2.25066471052546 | -1.66966902941181 | 12.96263975520444 |
| H | 1.99767288706314 | -2.43535373969315 | 13.69662234000647 |
| H | 3.72293215937041 | -0.73066910616176 | 14.23393387402519 |
| O | 5.33143452758028 | 4.29768742602544 | 11.11936617835647 |
| C | 5.33870398345004 | 3.14535206011111 | 11.56459459194467 |
| O | 6.38431064261277 | 2.33497356592729 | 11.44704511061989 |

**InCl – FC-Cl**

| | | | |
|---|---|---|---|
| In | 1.74753964180544 | 4.83427392600863 | 10.98584736986796 |
| O | 1.73856878830010 | 6.30919196144814 | 12.45505625525893 |
| N | 3.64803174659188 | 5.24698929931589 | 9.73067577644517 |
| C | 2.34185491832918 | 7.09118264798327 | 8.83042693403046 |
| N | 1.12126082314261 | 6.53130560321574 | 9.43639122957721 |
| C | 1.18402815681519 | 8.30766802042477 | 11.19993083011809 |
| C | 2.60391181968732 | 8.38149684551370 | 13.18468120553933 |
| C | 3.44979608806679 | 6.07934695121736 | 8.68876823498873 |
| C | 2.69509123079493 | 9.76272689737229 | 13.03046991369365 |
| C | 1.31347826596983 | 9.70024832505622 | 11.08185223839695 |
| C | 1.83306806597698 | 7.62485391169985 | 12.25766352298098 |
| C | 0.34764592967679 | 7.55098726880413 | 10.21294920399764 |
| C | 2.05886202775964 | 10.45512151569373 | 11.98646599700236 |
| C | 0.24126102433095 | 5.89777312681846 | 8.40830312087428 |
| C | 1.21237033350952 | 5.12940382407397 | 6.21004089832222 |
| C | 0.96859287585186 | 4.89304616070382 | 7.56895450450816 |
| C | 5.33910021504545 | 5.13036841261282 | 7.54319180685238 |
| C | 4.65608195313421 | 4.35775263925869 | 9.70416439566353 |

| | | | |
|---|---|---|---|
| C | 1.42756326308753 | 3.69920586142459 | 8.18051514087136 |
| C | 4.28785906313553 | 6.03968516534821 | 7.57085937923311 |
| C | 1.91940035938602 | 4.22141611531185 | 5.42113752933811 |
| C | 3.28934941547027 | 7.65829113795019 | 14.30662496915041 |
| C | 5.53597025078096 | 4.27729224366084 | 8.63266834254373 |
| C | 2.17772821383227 | 11.95128496972832 | 11.86021586373206 |
| O | 1.21883814519292 | 3.43759547588779 | 9.46600896431247 |
| C | 2.14650259573285 | 2.76187836215417 | 7.38575931531832 |
| C | 2.37691411509244 | 3.04557994316234 | 6.04154503765212 |
| C | 2.63810140982580 | 1.49979715782736 | 8.03111606341717 |
| C | 2.16275006770155 | 4.47029959232591 | 3.95571489282910 |
| H | 1.95324526333488 | 5.51547281997880 | 3.68807163019489 |
| H | 1.52071904289404 | 3.83466790657445 | 3.32433484113547 |
| H | 3.20420365320162 | 4.25122025657037 | 3.67457148722312 |
| H | 3.23011244852484 | 12.27265023071879 | 11.81439158769513 |
| H | 1.72226201032155 | 12.46795410695623 | 12.72006585369870 |
| H | 1.67674437439509 | 12.31430937770600 | 10.95211082302166 |
| H | 3.87544899488943 | 8.35182181476103 | 14.92437341382521 |
| H | 3.95521817607394 | 6.87090228817245 | 13.92131890382868 |
| H | 2.55894396921831 | 7.14171469886915 | 14.94786237879813 |
| H | 0.80348846700606 | 10.20387106364904 | 10.25400331258289 |
| H | 3.29313172388126 | 10.32661457827632 | 13.75376919534926 |
| H | 0.84045540207256 | 6.05677484678477 | 5.76158463957746 |
| H | 3.18969490492990 | 1.72053525931705 | 8.95796646555943 |
| H | 3.28626914052458 | 0.93179620945168 | 7.34999569067073 |
| H | 1.79476945691340 | 0.85695373608629 | 8.32809416012673 |
| H | 2.94137541968196 | 2.31974390366056 | 5.44675080973726 |
| H | -0.45749695837507 | 6.99449466137064 | 10.71795487371680 |
| H | -0.12538574766557 | 8.25658623150558 | 9.50430522827095 |
| H | 2.69274941474386 | 7.89237849682605 | 9.50315461672260 |
| H | 2.12427837400521 | 7.54563914712652 | 7.84840256621954 |
| H | -0.17968120760143 | 6.69705766267772 | 7.76936455368501 |
| H | -0.58557074787445 | 5.42284455463805 | 8.95872936687777 |
| H | 4.08599233301486 | 6.69645369935966 | 6.72496963436043 |
| H | 5.99226305512168 | 5.07393769213218 | 6.67139286185148 |
| H | 6.34421814562531 | 3.54705886918773 | 8.64505335363820 |
| H | 4.72191286348741 | 3.69273136991333 | 10.56855477625381 |
| C | -1.43795298245308 | -1.22861487840769 | 11.61150999106659 |
| C | -0.75442449202729 | -0.00812100638693 | 11.61251133862733 |
| C | 0.54552727993312 | 0.06330885355495 | 11.09124797923656 |
| H | 1.07907270043414 | 1.01658687841449 | 11.07031125427320 |
| C | 1.14773949273182 | -1.08551810379421 | 10.58067987823368 |
| H | 2.15668719986276 | -1.02398578415663 | 10.17008600615322 |
| C | 0.46817468389266 | -2.30735158379234 | 10.59287948927007 |
| H | 0.94664001384418 | -3.20298167049682 | 10.19332148689294 |
| C | -1.40425583756755 | 1.20754207723733 | 12.20531675877777 |
| C | -0.75234687790017 | 1.72383561756156 | 13.50173955904146 |
| H | -1.31755081621355 | 1.46989300756441 | 14.40515238028304 |
| H | 0.29515108681334 | 1.40300271373468 | 13.59184388857324 |
| O | -1.26143241039885 | 2.36203164392723 | 11.31718156491449 |
| H | -2.48493625185558 | 1.04078642999983 | 12.33514681999901 |
| C | -0.82640476646151 | -2.37887864489190 | 11.10925780227501 |

| | | | |
|---|---|---|---|
| H | -1.36509120887816 | -3.32757321654892 | 11.11354980062574 |
| H | -2.45674604709188 | -1.27805233118170 | 12.00378095620240 |
| O | -0.67621212948480 | 4.53415855730795 | 11.54450150941086 |
| C | -0.88356458685924 | 3.43472433596677 | 12.03095082821035 |
| O | -0.75853733861374 | 3.16146467220153 | 13.34188522177148 |
| Cl | 2.55669918932095 | 3.03842779611180 | 12.40748725762195 |

## [7] References


(1) Fu, L.-Z.; Zhou, L.-L.; Liang, Q.-N.; Fang, C.; Zhan, S.-Z. Synthesis and electro- and photo-catalytic properties of a dinuclear cobalt(III) complex supported by 2-pyridylamino-N,N-bis(2-methylene-4,6-bimethyl)phenol. *Polyhedron* **2016**, *107*, 83-88.

(2) CrysAlisPro: Data Collection, Integration Software, version 1.171.37.35; Agilent Technologies UK Ltd.: Oxford, U.K., **2011**.

(3) (a) Sheldrick, G. M. A Short History of SHELX. *Acta Crystallogr. Sect. A* **2008**, *A64*, 112-122. (b) Sheldrick, G. M. Crystal Structure Refinement with SHELXL. *Acta Crystallogr*. **2015**, C71, 3-8.

(4) Dolomanov, O. V.; Bourhis, L. J.; Gildea, R. J.; Howard, J. A.K.; Puschmann, H. J. OLEX2: A Complete Structure Solution, Refinement and Analysis Program. *J. Appl. Cryst*. **2009**, *42*, 339-341.

(5) MERCURY: (a) Bruno, I. J.; Cole, J. C.; Edgington, P. R.; Kessler, M. K.; Macrae, C. F.; McCabe, P.; Pearson, J.; Taylor, R. New Software for Searching the Cambridge Structural Database and Visualizing Crystal Structures. *Acta Crystallogr., Sect. B: Struct. Sci*. **2002**, *B58*, 389-397. (b) Macrae, C. F.; Edgington, P.R.; McCabe, P.; Pidcock, E.; Shields, G. P.; Taylor, R.; Towler, M.; van de Streek, J. Mercury: Visualization and Analysis of Crystal Structures. *J. Appl. Cryst*. **2006**, *39*, 453-457.

(6) Neese, F. Software Update: The ORCA Program System, Version 4.0. *WIREs Comput. Mol. Sci.* **2017**, *8*, e1327.

(7) Neese, F. An Improvement of the Resolution of the Identity Approximation for the Formation of the Coulomb Matrix. *J. Comp. Chem*. **2003**, *24*, 1740-1747.

(8) (a) Grimme, S.; Ehrlich, S.; Goerigk, L. Effect of the Damping Function in Dispersion Corrected Density Functional Theory. *J. Comp. Chem*. **2011**, *32*, 1456-1465; (b) Grimme, S. Semiempirical GGA-type Density Functional Constructed with a Long-range Dispersion Correction. *J. Comput. Chem*. **2006**, *27*, 1787-1799.

(9) (a) Weingrad, F.; Aldrichs, R. Balanced Basis Sets of Split Valence, Triple Zeta Valence and Quadruple Zeta Valence Quality for H to Rn: Design and Assessment of Accuracy. *Phys. Chem. Chem. Phys*. **2005**, *7*, 3297-3305; (b) Schaefer, A.; Horn, H.; Aldrichs, R. Fully Optimized Contracted Gaussian Basis Sets for Atoms Li to Kr. *J. Chem. Phys*. **1992**, *97*, 2571-2577.

(10) (a) Helmich-Paris, B.; de Souza, B.; Neese, F.; Izsák, R. An Improved Chain of Spheres for Exchange Algorithm. *J. Chem. Phys*. **2021**, *155*, 104109; (b) Mardirossian, N.; Head-Gordon, M. ωB97M-V: A Combinatorially Optimized, Range-separated Hybrid, Meta-GGA Density Functional with VV10 Nonlocal Correlation. *J. Chem. Phys*. **2016**, *144*, 214110.

(11) Marenich, A. V.; Cramer, C. J.; Truhlar, D. G. Universal Solvation Model Based on Solute Electron Density and on a Continuum Model of the Solvent Defined by the Bulk Dielectric Constant and Atomic Surface Tensions. *Phys. Chem. B* **2009**, *113*, 6378-6396.

(12) González Fabra, J.; Castro-Gómez, F.; Sameera, W. M. C.; Nyman, G.; Kleij, A. W.; Bo, C. Entropic Corrections for the Evaluation of the Catalytic Activity in the Al(III) Catalysed Formation of Cyclic Carbonates from $CO_2$ and Epoxides. *Catal. Sci. Technol*. **2019**, *9*, 5433-5440.

(13) (a) Harvey, J. N.; Himo, F.; Maseras, F.; Perrin, L. Scope and Challenge of Computational Methods for Studying Mechanism and Reactivity in Homogeneous Catalysis. *ACS Catal*. **2019**, *9*, 6803-6813; (b) Ryu, H.; Park, J.; Kim, H. K.; Park, J. Y.; Kim, S.-T.; Baik, M.-H. Pitfalls in

Computational Modeling of Chemical Reactions and How to Avoid Them. *Organometallics* **2018**, *37*, 3228-3239.

(14) Hanwell, M. D.; Curtis, D. E.; Lonie, D. C.; Vandermeersch, T.; Zurek, E.; Hutchison, G. R. Avogadro: An Advanced Semantic Chemical Editor, Visualization, and Analysis Platform. *J. Cheminform*. **2012**, *4*, 17.
